# Deletion of *Cd44* Inhibits Metastasis Formation of Liver Cancer in *Nf2*-Mutant Mice

**DOI:** 10.3390/cells12091257

**Published:** 2023-04-26

**Authors:** Monserrat Gerardo-Ramírez, Vanessa Giam, Diana Becker, Marco Groth, Nils Hartmann, Helen Morrison, Helen L. May-Simera, Markus P. Radsak, Jens U. Marquardt, Peter R. Galle, Peter Herrlich, Beate K. Straub, Monika Hartmann

**Affiliations:** 1 Department of Medicine I, University Medical Center of the Johannes Gutenberg University, 55131 Mainz, Germany; 2 Leibniz Institute on Aging-Fritz Lipmann Institute (FLI), 07745 Jena, Germany; 3 Institute of Pathology, University Medical Center of the Johannes Gutenberg University, 55131 Mainz, Germany; 4 Faculty of Biological Sciences, Friedrich-Schiller University, 07745 Jena, Germany; 5 Cilia Cell Biology, Institute of Molecular Physiology, Johannes Gutenberg University, 55128 Mainz, Germany; 6 Department of Medicine III, University Medical Center of the Johannes Gutenberg University, 55131 Mainz, Germany; 7 Department of Medicine I, University Medical Center Schleswig-Holstein, Campus Lübeck, 23558 Lübeck, Germany

**Keywords:** CD44, Merlin, NF2, liver cancer, HCC, ICAM-1, metastasis, Hippo signaling

## Abstract

Primary liver cancer is the third leading cause of cancer-related death worldwide. An increasing body of evidence suggests that the Hippo tumor suppressor pathway plays a critical role in restricting cell proliferation and determining cell fate during physiological and pathological processes in the liver. Merlin (Moesin-Ezrin-Radixin-like protein) encoded by the *NF2* (neurofibromatosis type 2) gene is an upstream regulator of the Hippo signaling pathway. Targeting of Merlin to the plasma membrane seems to be crucial for its major tumor-suppressive functions; this is facilitated by interactions with membrane-associated proteins, including CD44 (cluster of differentiation 44). Mutations within the CD44-binding domain of Merlin have been reported in many human cancers. This study evaluated the relative contribution of CD44- and Merlin-dependent processes to the development and progression of liver tumors. To this end, mice with a liver-specific deletion of the *Nf2* gene were crossed with *Cd44*-knockout mice and subjected to extensive histological, biochemical and molecular analyses. In addition, cells were isolated from mutant livers and analyzed by in vitro assays. Deletion of *Nf2* in the liver led to substantial liver enlargement and generation of hepatocellular carcinomas (HCCs), intrahepatic cholangiocarcinomas (iCCAs), as well as mixed hepatocellular cholangiocarcinomas. Whilst deletion of *Cd44* had no influence on liver size or primary liver tumor development, it significantly inhibited metastasis formation in *Nf2*-mutant mice. CD44 upregulates expression of integrin β2 and promotes transendothelial migration of liver cancer cells, which may facilitate metastatic spreading. Overall, our results suggest that CD44 may be a promising target for intervening with metastatic spreading of liver cancer.

## 1. Introduction

As the third leading cause of cancer-related mortality (830,000 deaths worldwide in 2020), primary liver cancer represents a major public health challenge [1]. The most common histologic type of primary liver cancer, hepatocellular carcinoma (HCC), constitutes approximately 75–85% of primary liver cancer worldwide [1]. Intrahepatic cholangiocarcinoma (iCCA) is the second most common, comprising 10–15% of primary liver cancer cases [1]. Mixed hepatocellular cholangiocarcinoma, presenting histologic features of both HCC and iCCA, can also be observed in some cases [2]. Despite several therapeutic improvements over the past decade [3], survival rates of liver cancer patients remain stubbornly low [4]. Most challenging is metastatic liver cancer, which is associated with a very short five-year survival rate (3.1%, NCI 2022) and limited treatment options [3,4]. Therefore, a better understanding of processes implicated in liver tumor development and progression is essential to develop effective treatment strategies.

The Hippo tumor suppressor pathway plays a critical role in restricting cell proliferation and determining cell fate during physiological and pathological processes in the liver [5,6,7,8,9,10,11,12,13,14,15,16]. The pathway consists of a cascade of kinases that inactivate its primary effector, Yes-associated protein (YAP), by promoting its phosphorylation and cytoplasmic localization with concomitant proteolytic degradation [10]. The core of the mammalian Hippo pathway involves a kinase cascade, including the mammalian STE20-like protein kinase 1 and 2 (MST1/2) and the large tumor suppressor kinase 1 and 2 (LATS1/2) [17]. MST1/2 binds to the Salvador homolog 1 (SAV1) adaptor protein and phosphorylates and activates LATS1/2, which is bound to an adapter protein MOB1A/1B (Mps one binder homolog A and B) [17]. Subsequently, active LATS1/2 phosphorylates YAP and its transcriptional co-activator with PDZ-binding motif (TAZ), leading to retention of YAP in the cytoplasm, upon activation of the Hippo pathway [15]. In contrast, repression of the Hippo pathway causes YAP/TAZ dephosphorylation and their translocation into the nucleus, wherein YAP/TAZ interact with other transcription factors—such as TEA domain (TEAD)—to induce the expression of genes involved in cell migration, proliferation and survival (reviewed in [17]). Interestingly, more than 50% of human liver tumors show increased expression and nuclear activation of YAP, implying that dysregulation of the Hippo signaling pathway might be a frequent event during malignant transformation of liver cells [15,18,19].

Dysregulation of the Hippo pathway signaling components prompts liver overgrowth and promotes development of metastatic liver tumors [5,6,7,8,9,10,11,12,13,14,15,16]. This demonstrates a need for precise regulation. One of the upstream regulators of the Hippo signaling pathway is Merlin (Moesin-Ezrin-Radixin-like protein), encoded by the *NF2* (neurofibromatosis type 2) gene [17]. Germline mutations in this gene cause the hereditary tumor syndrome neurofibromatosis type 2 (NF2), which is primarily characterized by an enhanced predisposition to developing tumors of the central and peripheral nervous system [20]. Deactivating *NF2* mutations have been identified in human liver cancers, including 1.9% of HCCs and 5.3% of iCCAs [21]. Additionally, suppression of Merlin function in the liver may occur on an epigenetic or post-transcriptional level [22]. Accordingly, it has been demonstrated that expression of Merlin is decreased in a high proportion of human liver tumors when compared to normal tissues [22]. Studies in mice emphasize the role of Merlin in the pathogenesis of liver tumors. A liver-specific deletion of the *Nf2* gene leads to liver enlargement and promotes tumorigenesis [5,6].

The tumor suppressor function of Merlin is not restricted to the regulation of Hippo signaling. Merlin has been shown to restrict cell proliferation and migration by inhibiting expression and activation of receptor tyrosine kinases and downstream signaling pathways, including RAS/RAF/MEK/ERK, RAC/PAK/JNK, PI3K/AKT/JNK, FAK/SRC, mTORC1 and WNT/β-catenin [23,24]. It has also been shown to attenuate oncogenic gene expression via inhibition of the CRL4/DCAF1 (Cullin-RING ubiquitin ligase 4 / DDB1- and Cul4-associated factor 1) ubiquitin ligase complex [20,24,25]. Of interest, the majority of mutations, both in the germline of NF2 patients and in sporadically occurring human tumors, have been identified in the *NF2* gene region encoding the so-called FERM (4.1 protein, ezrin, radixin, moesin) domain, which mediates interactions with membrane-bound proteins [26]. These observations suggest that the interactions mediated by the FERM domain might be critical to Merlin’s tumor suppressor function.

CD44 (cluster of differentiation 44) is a type I transmembrane glycoprotein and an important interaction partner of Merlin, binding to its FERM domain [20,27,28,29]. Expression of CD44 is upregulated in numerous human cancers, positing a pro-tumorigenic role [30,31]. CD44 is consistently identified as an important diagnostic and prognostic marker in primary liver tumors, correlating with TNM classification and poor prognosis in HCC and CCA patients [32,33,34,35,36]. CD44 exerts multiple molecular functions and may influence tumor cell behavior in several ways. First, it modulates cell adhesion by interacting with hyaluronic acid (HA) and other components of the extracellular matrix [30,37]. Second, certain CD44 isoforms containing amino acid sequences that are encoded by the variant exon v3 or v6 regulate activation of several receptor tyrosine kinases (RTKs) and downstream signaling [27,37,38]. In particular, the CD44v6-bearing isoforms were shown to be required for activation of receptor tyrosine kinase MET [39]. This coreceptor function is twofold; on one hand, the extracellular region of CD44v6 binds hepatocyte growth factor (HGF) and is necessary for MET activation. On the other hand, the cytoplasmic domain of CD44 promotes downstream signaling [38,39]. Lastly, CD44 undergoes sequential proteolytic cleavages and the intracellular domain of CD44, which is cleaved by γ-secretase, plays a role in transcriptional regulation [40].

The aim of the current study was to evaluate in detail the collaboration between CD44 and Merlin in primary liver tumor development and progression, as well as to investigate molecular mechanisms underlying CD44 and Merlin function in the pathogenesis of liver tumors. Several studies indicate that Merlin exerts its tumor suppressor activity in part by negatively regulating CD44 function [20,27,28,29]. Binding of Merlin to CD44 occurs in growth inhibitory conditions [27]. In growth promoting conditions, CD44 is released from the inhibitory interaction with Merlin to promote oncogenic signaling [27]. This led us to hypothesize that disruption of the Merlin-CD44 complex, through loss of Merlin, releases putative tumor- or metastasis-promoting functions of CD44. To evaluate the relevance of this interaction in vivo, mice with a liver-specific deletion of the *Nf2* gene were crossed to *Cd44*-knockout mice. Our results suggest that CD44 promotes transendothelial migration of *Nf2*-deficient liver tumor cells, thus facilitating metastasis formation. We also suggest that intercellular adhesion molecule 1 (ICAM-1)—and potentially other molecules—may compensate for loss of CD44 in other tumor relevant processes. 

## 2. Materials and Methods

### 2.1. Experiments Involving Animals

In total, 122 mice aged between 2 and 48 weeks were analyzed to investigate the influence of NF2 and CD44 on different stages of liver tumor development and progression. All animals used in this study were housed under constant temperature and humidity conditions, followed a 12-h light/dark cycle, and had ad libitum access to food and water. All experiments involving mice were approved by the local authorities (AZ: 23 177-07/G 16-1-028). *Alb-Cre* (B6.Cg-Tg(Alb-cre)21Mgn/J) mice were from The Jackson Laboratory (Sacramento, CA, USA). Generation and genotyping of *Nf2^flox/flox^* transgenic mice (with targeted exon 2) was described by Giovannini et al. [41]. Generation of *Cd44^flox/flox^* mice (with targeted exon 3) was described by Dhar et al. (2018) [42]. Generation and genotyping of *Cd44^−/−^* mice was described by Ma et al. (2020) [43]. All mice were inbred onto C57BL/6 genetic background. *Nf2^flox/flox^*;*Alb-Cre*; and *Cd44*-deficient mice were crossed to obtain the desired phenotypes. *Nf2^flox/flox^*;*Alb-Cre*; mice were monitored weekly for occurrence of tumors or decline of health, then euthanized after two years at the latest and analyzed.

### 2.2. Detection of α-Fetoprotein (AFP), Alanine Aminotransferase (ALT), and Bilirubin in Mouse Serum

Serum was isolated using Microvette 500 Z-GEL Gel/clotting activator tubes (Sarstedt AG & Co. KG, Nümbrecht, Germany) per manufacturer’s instructions. Levels of α-fetoprotein (AFP), alanine aminotransferase (ALT), and bilirubin were detected in serum of 32- and 48-week-old mice using commercially available kits (AFP, catalog number MAFP00, R&D Systems, Inc., Minneapolis, Minnesota, USA; ALT, catalog number ab282882, Abcam, Berlin, Germany; bilirubin, catalog number NBP2-69939, Bio-Techne GmbH, Wiesbaden Nordenstadt, Germany) per manufacturer’s protocols.

### 2.3. Histochemistry and Immunohistochemistry

Tissue samples were fixed in 4% paraformaldehyde (PFA) and embedded in paraffin. Next, 4 µm thick sections were deparaffinized, re-hydrated, and stained by hematoxylin and eosin (H&E) or were used for antigen detection by immunohistochemistry or immunofluorescence microscopy. Conventional H&E and Gomori silver staining were performed, following standard procedures, at the Institute of Pathology, Mainz. Detection of antigens was conducted on tissue sections after antigen-retrieval in the target retrieval solution, citrate pH 6 (DakoCytomation GmbH, Hamburg, Germany). Tissue sections were permeabilized in 0.1% Triton X-100 (Carl Roth GmbH, Karlsruhe, Germany) in phosphate-buffered saline (PBS, Life Technologies GmbH, Darmstadt, Germany) at room temperature for 7 min. Afterwards, tissue sections were washed with PBS. Endogenous peroxidases were quenched using 3% H_2_O_2_ (Carl Roth GmbH, Karlsruhe, Germany) in methanol at room temperature for 10 min (this step was performed only for subsequent chemiluminescent detection). Tissue sections were again washed with PBS. Unspecific binding was blocked by pre-incubation with blocking solution consisting of 2% normal horse serum (Vector Laboratories, Newark, CA, USA), 1% bovine serum albumin (BSA, Sigma-Aldrich, Taufkirchen, Germany), and 0.01% Triton X-100 in PBS. Primary antibodies were diluted in blocking solution and applied on sections at 4 °C overnight. Primary antibodies used for immunohistochemistry are listed in Table 1. After vigorous washings with 0.01% Triton X-100 and 0.01% Tween20 in PBS, sections were incubated either with the fluorophore-conjugated secondary antibody diluted in blocking solution for 1 h, or with ZytoChem HRP One-Step Polymer anti-Mouse/Rabbit/Rat solution (Zytomed Systems GmbH, Berlin, Germany) at room temperature for 30 min. Afterwards, sections were again washed with 0.01% Triton X-100 and 0.01% Tween20 in PBS. For fluorescent detection, the sections were embedded in ProLong Diamond Antifade Mountant with DAPI (Thermo Fisher Scientific GmbH, Darmstadt, Germany). Alternatively, chemiluminescent detection was performed using Liquid DAB+Substrate Chromogen System (DakoCytomation GmbH, Hamburg, Germany), following manufacturer’s instructions. Afterwards, the nuclei were counterstained with hematoxylin (Merck KGaA, Darmstadt, Germany) for 20 sec, and the samples were then dehydrated and embedded in xylene-based mounting medium (Kindler GmbH & Co., Freiburg, Germany). Paraffin-embedded lung, liver, and tumor sections were evaluated by an expert pathologist (B.K.S).

### 2.4. TUNEL Assay

Click-iT^®^ Plus TUNEL Assay (catalog number C10618, Life Technologies GmbH, Darmstadt, Germany) was applied for in situ apoptosis detection, per manufacturer’s protocol. To induce DNA strand breaks for positive control, fixed and permeabilized *Alb-Cre* liver sections were incubated with 1 unit of Dnase I (Qiagen, Hilden, Germany) diluted in 1xDNase I Reaction Buffer (20 mM Tris-HCl, pH 8.4, 2 mM MgCl2, 50 mM KCl), at room temperature for 30 min.

### 2.5. Isolation of Primary Liver Cells

Primary liver tumors were isolated from *Cd44^+/+^,Nf2^flox/flox^*;*Alb-Cre* and *Cd44^-/-^,Nf2^flox/flox^*;*Alb-Cre* mice, washed with PBS, and minced into 1–3 mm^3^ pieces in PBS with use of a scalpel. Tissue fragments were dissociated in 1 mg/mL of Collagenase IV (Life Technologies GmbH, Darmstadt, Germany) in Dulbecco’s Modified Eagle’s Medium (DMEM, Life Technologies GmbH, Darmstadt, Germany) at 37 °C for 60 min. Cell aggregates and larger particles were removed by filtering the suspension through 70 µm MACS Smart cell strainer (Miltenyi Biotec B.V. & Co. KG, Bergisch Gladbach, Germany). Afterwards, dissociated cells were recovered by centrifugation at 350–400× *g* for 5 min. Cells were cultured in DMEM/F12 (Thermo Fisher Scientific GmbH, Darmstadt, Germany) supplemented with 10% fetal bovine serum (FBS, PAN-Biotech GmbH, Aidenbach, Germany), 1% penicillin-streptomycin (Sigma-Aldrich, Taufkirchen, Germany) and 1% HEPES (Life Technologies GmbH, Darmstadt, Germany). Outgrowing cells were replated on fresh plates. After approximately 10 passages, the cultures presented a macroscopically homogenous population of liver cells and were subsequently cultured in DMEM (Life Technologies GmbH, Darmstadt, Germany) supplemented with 10% FBS and 1% penicillin-streptomycin.

### 2.6. Sources of Other Cells and Cell Culture Conditions

NIH3T3 cells (immortalized Swiss mouse embryonic fibroblasts) were from the European Collection of Animal Cell Cultures (Salisbury, UK). Hepa1-6 mouse hepatoma cell line was from Sigma-Aldrich (Taufkirchen, Germany). C57BL/6 mouse primary lung microvascular endothelial cells were from Biotrend Chemikalien GmbH (Köln, Germany). The latter were cultured in complete mouse endothelial cell medium (Biotrend Chemikalien GmbH, Köln, Germany) on plates coated with gelatin (Sigma-Aldrich, Taufkirchen, Germany). Established liver cell lines, NIH3T3 and Hepa1-6 cells were cultured in DMEM supplemented with 10% FBS and 1% penicillin-streptomycin. All cells were maintained in a humidified atmosphere with 5% CO2 at 37 °C. All experiments were performed with mycoplasma-free cells.

### 2.7. Clonogenic Assay under Cell Attachment Conditions

Liver cells were seeded in triplicates at a density of 100 and 1,000 cells per well in a 6-well plate, in DMEM supplemented with 10% FBS. Medium was changed every 3 days. After 1 week, the colonies were fixed in 4% PFA for 5 min and stained with 0.05% crystal violet in PBS for 30 min. Photographs of colonies were taken on a ChemiDoc MP Imaging System (Bio-Rad Laboratories GmbH, Feldkirchen, Germany), and the number of colonies was counted using Cell Counter software v0.2.1 (by Nghia Ho). 

### 2.8. Colony Growth under Nonadherent Conditions/Anoikis Assay 

First, 96-well plates were made anchorage-resistant by coating them with a 1% agar layer. Liver cells were seeded in triplicates at a density of 0.2 × 10^5^ cells per well on an anchorage-resistant 96-well plate in DMEM supplemented either with 10% FBS or without FBS. After 7 days of culture, the colonies were photographed using a Leica DFC290 microscope, and the colony surface area was measured using ImageJ software (National Institutes of Health (NIH), Bethesda, MD, USA).

### 2.9. Scratch Migration Assay 

Liver cell lines were grown on 6-well plates in DMEM, supplemented with 10% FBS. Scratches were generated in confluent cell monolayers with a 200 µL pipette tip, and cell debris was removed by washing with PBS. Afterwards, the cells were allowed to migrate into the generated gap in serum-free medium for 72 h. To inhibit proliferation, cytosine b-D-arabinofuranoside (AraC, Sigma-Aldrich, Taufkirchen, Germany) was added at a concentration of 10 µM prior to migration assay. The cells were photographed using a Leica DFC290 microscope (Leica Microsystems CMS GmbH, Mannheim, Germany), and the cell-free gap was measured using ImageJ software. 

### 2.10. Tumor Cell Transendothelial Migration Assay

The ability of tumor cells to migrate through a confluent monolayer of endothelial cells towards a chemoattractant was tested using commercially available QCM tumor cell transendothelial migration assay (catalog number ECM558, Merck KGaA, Darmstadt, Germany), per manufacturer’s protocol. Briefly, tumor cells were allowed to migrate at 37 °C for 23 h through a confluent monolayer of tumor necrosis factor-*α* (TNF*α*, Thermo Fisher Scientific GmbH, Darmstadt, Germany) -stimulated C57BL/6 mouse primary lung microvascular endothelial cells, from medium without serum towards medium supplemented with 10% FBS. 

### 2.11. Western Blot

Cells and liver tissues were harvested in RIPA (50 mM Tris HCl pH 8.0; 150 mM NaCl; 1% NP40; 0.1% SDS; 1 mM EDTA) and M-PER (Thermo Fisher Scientific GmbH, Darmstadt, Germany) lysis buffers, respectively, containing a halt protease and phosphatase inhibitor cocktail. Protein concentration was determined using the bicinchoninic acid (BCA) protein assay kit (Thermo Fisher Scientific GmbH, Darmstadt, Germany). Lysates were mixed with SDS-PAGE sample buffer (final concentration: 50 mM Tris HCl, pH 6.8; 2% SDS; 10% glycerol; 0.1% bromphenol; 100mM dithiotreitol) and denatured by boiling at 97 °C for 7 min. Proteins were separated on 8% acrylamide gels using SDS-PAGE and transferred to nitrocellulose membranes. Blocking was performed at room temperature for 1 h, using 5% low-fat milk powder in Tris-buffered saline (TBS) containing 0.1% Tween20 solution (TBS-T). Membranes were incubated with primary antibody at 4 °C overnight, followed by HRP-labeled secondary antibody at room temperature for 1 h. Primary and secondary antibodies used for immunoblot analysis are listed in Table 1 and Table 2. A total of 5% BSA in TBS-T was used as diluent for CD44 antibody, clone E7K2Y. All other primary and secondary antibodies were diluted in 5% low-fat milk powder in TBS-T. Blots were developed using Clarity Western ECL Substrate (Bio-Rad Laboratories GmbH, Feldkirchen, Germany) and visualized with Chemi-Doc MP Imaging System (Bio-Rad Laboratories GmbH, Feldkirchen, Germany). The intensity of immunoblot bands was quantified with ImageJ software.

### 2.12. Immunocytochemistry

Liver cells grown on coverslips were fixed with 4% PFA in PBS for 20 min, washed in PBS, and permeabilized with 0.1% Triton X-100 in PBS for 7 min. Non-specific epitopes were blocked with blocking solution (1% BSA in PBS) for 45 min. Cells were probed in primary antibody solution at room temperature for 1 h, washed extensively with PBS, and incubated with fluorophore-conjugated secondary antibody solution at room temperature for 1 h. After additional washing with PBS, the cells were mounted on cover slides with a ProLong™ Diamond Antifade Mountant with DAPI (Thermo Fisher Scientific GmbH, Darmstadt, Germany). Primary and secondary antibodies used for immunofluorescence (IF) detection are listed in Table 1. A total of 1% BSA in PBS was used as diluent for all primary and secondary antibodies. For negative controls, primary antibody was omitted.

### 2.13. Antibodies

Table 1 and Table 2 list antibodies used in the study.

### 2.14. RNA Sequencing and Gene Expression Analysis

Liver tissues were derived from *Cd44^+/+^*;*Nf2^Δ2/+^* and *Cd44^-/-^*;*Nf2^Δ2/+^* mice described previously [43]. Three samples of each group were analyzed. Total RNA was isolated using RNeasy mini kit (Qiagen), per manufacturer’s instructions. Sequencing of RNA samples was achieved using Illumina’s next-generation sequencing methodology [44]. The quality check and quantification of total RNA was completed using the Agilent Bioanalyzer 2100 in combination with the RNA 6000 nano kit (Agilent Technologies). Library preparation required Illumina’s TruSeq stranded mRNA library preparation kit, per manufacturer’s description. Agilent Bioanalyzer 2100 in combination with the DNA 7500 kit enabled quantification and quality check of libraries. Said libraries were sequenced in two lanes on a HiSeq2500 running in 51 cycle/single-end/high-output mode. Sequence information was extracted in FastQ format using Illumina’s bcl2fastq v.1.8.4 and resulted in an average of around 24 million reads per sample. Reads were aligned to the mouse reference genome sequence (ENSEMBL Mus musculus GRCm38.84) using HISAT2 (hisat2-2.0.2-beta), with read counts determined by read summarization with feature Counts (subread-2.0.0). R programming language and related packages were engaged to further analyze RNA sequencing data. Differentially regulated genes were determined using the rlog function of the DESeq2 package. All genes with Benjamin Hochberg adjusted *p*-value (padj) < 0.05 were considered significant. 

### 2.15. Real-Time Quantitative Reverse Transcription PCR (Real-Time qRT-PCR)

Total RNA was isolated from mouse tissues or cell lines using PeqGOLD total RNA kit (VWR International GmbH, Darmstadt, Germany), per manufacturer’s instructions. cDNA was prepared from 1 µg of total RNA using iScript cDNA synthesis kit (Bio-Rad Laboratories GmbH, Feldkirchen, Germany) and used for qRT-PCR reaction at a final dilution of 1:75. We used iQ^TM^SYBR green supermix (Bio-Rad Laboratories GmbH, Feldkirchen, Germany) on CFX Connect™ real-time PCR detection system (Bio-Rad Laboratories GmbH, Feldkirchen, Germany) for PCR amplification. Oligonucleotides were used at a final concentration of 157 nM. *Tbp* housekeeping gene was used as the reference for testing efficiency of *Icam1* knockdown, with *Hprt1* and *Actin* housekeeping genes used as references for all other real-time qRT-PCRs. Total *Cd44* was detected using primers that recognized constant regions of all isoforms (Cd44all Fwd1 and Cd44all Rev1). CD44 isoforms containing variant exon sequences were amplified using forward oligonucleotides specific for stable exon 5 (stable Ex5 Fwd 1) and reverse oligonucleotides specific for appropriate variant exon. Oligonucleotides used for real-time qRT-PCR are listed in Table 3. Relative expression values were calculated using REST-MCS software [45].

### 2.16. Small Interfering RNA (siRNA) Transfections

siRNAs targeting mouse *Icam1* (ID:s67995 and s67994), mouse *Cd44* (ID:s63660 and s63661), and negative control siRNA No.1 were acquired from Thermo Fisher Scientific GmbH (Darmstadt, Germany). All siRNA transfections were performed in 6-well plates using the liposomal transfection reagent Lipofectamine 2000 (Thermo Fisher Scientific GmbH, Darmstadt, Germany), per manufacturer’s instructions.

### 2.17. Statistical Analysis

The Chi-Square test of independence determined whether there is a significant association between the presence or absence of metastases in *Cd44*-positive and *Cd44*-negative livers; and presence or absence of variant *Cd44* isoforms in liver tumors and control livers. For all quantitative analyses, comparisons between groups were made with unpaired Student’s *t*-test unless otherwise stated. Two-tailed *p*-value ≤ 0.05 was considered as significant (ns: *p* > 0.05, *: *p* ≤ 0.05, **: *p* ≤ 0.01, ***: *p* ≤ 0.001, ****: *p* ≤ 0.0001). 

## 3. Results

### 3.1. Deletion of Cd44 Does Not Influence Progressive Liver Enlargement in Nf2^flox/flox^;Alb-Cre Mice

We used *Nf2*-mutant mice to model liver tumor development and progression. Deficiency of *Nf2* was confined to the liver by crossing conditional *Nf2*-mutant (*Nf2^flox/flox^*) mice with albumin-Cre (*Alb-Cre*) transgenic mice. To investigate the role of CD44 in liver tumor development and progression, *Nf2^flox/flox^*;*Alb-Cre* mice were crossed with *Cd44*-mutant mice, either *Cd44^flox/flox^*, to obtain deletion of *Cd44* exclusively in the liver, or *Cd44^−/−^*, resulting in deletion of *Cd44* in the entire body. A total of 122 mice aged between 2 and 48 weeks were analyzed to investigate the influence of NF2 and CD44 on different stages of liver tumor development and progression. *Alb-Cre* mice were the control group. Table 4 lists the number of mice analyzed at different ages. 

**Figure 1:**
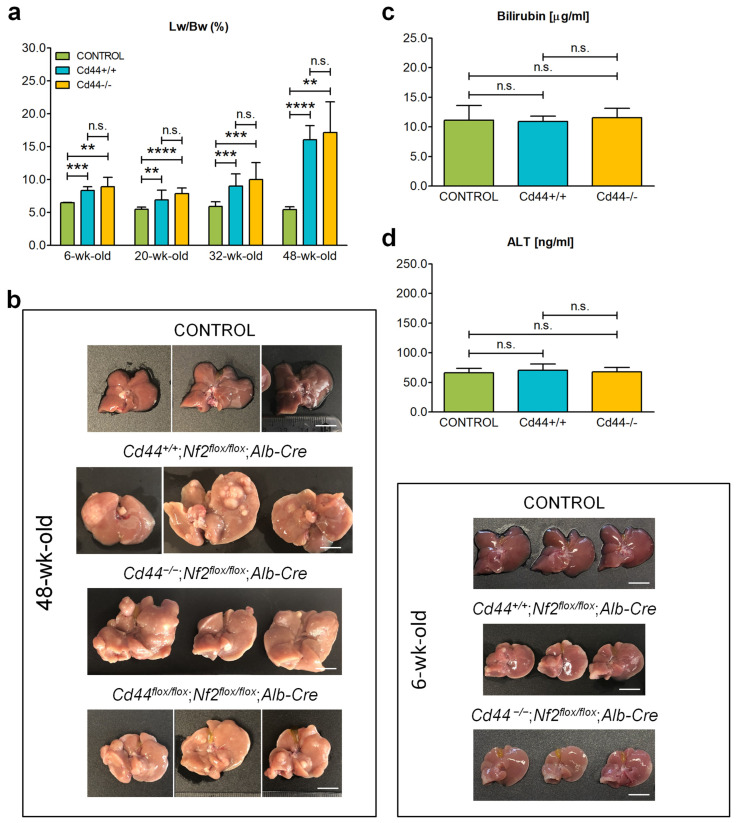
Influence of *Cd44* deletion on progressive liver enlargement in *Nf2^flox/flox^;Alb-Cre* mice. (**a**) Distribution of liver weight/body weight (Lw/Bw) ratios in *Cd44^+/+^;Nf2^flox/flox^;Alb-Cre* (turquoise), *Cd44^−/−^;Nf2^flox/flox^;Alb-Cre* (yellow) versus control *Alb-Cre* (green) mice between 6 and 48 weeks old. The bar chart shows mean percent ratio of liver weight/body weight (Lw/Bw) ±SD. The number of mice analyzed at different ages is as indicated in Table 4. “wk-old” stands for week-old. (**b**) Example pictures of livers isolated from *Cd44^+/+^;Nf2^flox/flox^;Alb-Cre*, *Cd44^−/−^; Nf2^flox/flox^;Alb-Cre*, *Cd44^flox/flox^;Nf2^flox/flox^;Alb-Cre* and control (*Alb-Cre*) mice at 6- and 48-weeks-old. Scale bar: 1 cm. (**c**) Serum level of bilirubin in 32-week-old mice. Bilirubin level was measured by immunoassay in serum of eight control, nine *Cd44^+/+^;Nf2^flox/flox^;Alb-Cre* and eight *Cd44^−/−^;Nf2^flox/flox^;Alb-Cre* mice. The bar chart shows mean level of bilirubin (µg/mL) ±SD. (**d**) Serum level of ALT in 32-week-old mice. ALT level was measured by immunoassay in serum of four control, six *Cd44^+/+^;Nf2^flox/flox^;Alb-Cre* and six *Cd44^−/−^;Nf2^flox/flox^;Alb-Cre* mice. The bar chart shows mean level of ALT (ng/mL) ± SD. Student’s *t*-test two-tailed values: n.s.: *p* > 0.05, **: *p* ≤ 0.01, ***: *p* ≤ 0.001, ****: *p* ≤ 0.0001.

The presence of the desired genetic modification was confirmed by PCR-based genotyping (Figure S1). As expected, recombinant deleted alleles of *Cd44* and *Nf2* were only detected in livers of conditional knockout mice (Figure S1). In contrast, *Cd44*^−/−^ mice exhibited allele deletion in all analyzed tissues (Figure S1). Presence of a faint floxed band of *Cd44* and *Nf2* gene in the liver of conditional knockout mice may indicate non-complete recombination of floxed alleles or infiltration by non-hepatic cells, which is consistent with previous reports on *Alb-Cre*-mediated excision [6,46,47]. The *Nf2^flox/flox^*;*Alb-Cre* mice were born at expected Mendelian rations and appeared healthy. The most prominent difference from control animals was progressive liver enlargement observed in all analyzed *Nf2^flox/flox^;Alb-Cre* mice, independent of the *Cd44* gene status (Figure 1a,b). It should be mentioned that deletion of the *Cd44* gene alone has no influence on normal liver phenotype [43,48,49]. The liver weight/body weight ratio of *Cd44^+/+^;Nf2^flox/flox^;Alb-Cre* animals increased from 6.94% at 20 weeks to 16.06% at 48 weeks, whereas the percent ratio of liver weight/body weight of control animals remained relatively constant (5.52% at 20 weeks and 5.46% at 48 weeks) during the analysis period. In addition, livers isolated from *Cd44^+/+^*;*Nf2^flox/flox^*;*Alb-Cre*, *Cd44^−/−^*;*Nf2^flox/flox^*;*Alb-Cre* and *Cd44^flox/flox^*;*Nf2^flox/flox^*;*Alb-Cre* mice appeared paler and more solid than control livers and harbored tumors at later ages (32- and 48-weeks) (Figure 1b).

We tested liver biomarkers in the serum to analyze the function of enlarged livers (Figure 1c,d), but found no significant difference in serum levels of bilirubin (Figure 1c) or alanine aminotransferase (ALT, Figure 1d) between control and *Nf2^flox/flox^;Alb-Cre* mice, which indicates normal function of biliary system and no significant hepatocyte injury, respectively. Thus, deletion of the *Nf2* gene in the liver and concomitant liver enlargement is not associated with impairment of liver function. Consistently, during this analysis (maximally 48 weeks) we observed no increased mortality in *Nf2^flox/flox^;Alb-Cre* mice compared to control group. Nor did we observe any significant differences in the appearance or size of the liver between *Cd44*-positive and *Cd44*-negative *Nf2^flox/flox^*;*Alb-Cre* mice. Thus, loss of the *Cd44* gene does not influence the liver overgrowth observed in *Nf2*-mutant mice. 

### 3.2. Ductular Proliferation Observed in Nf2^flox/flox^;Alb-Cre Mice Is Not Influenced by Loss of Cd44

**Figure 2:**
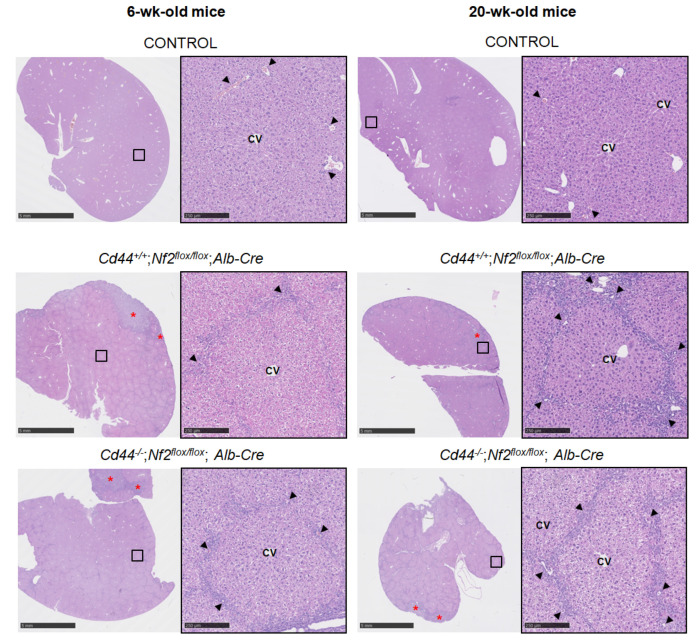

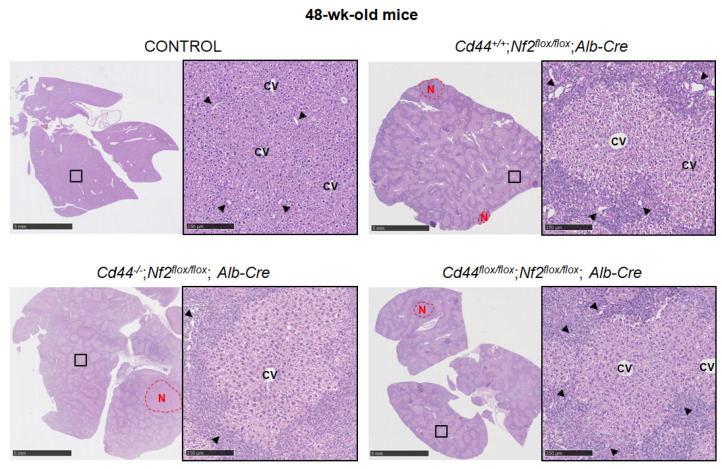
H&E-stains of livers of 6-, 20- and 48-week-old mice. Representative photographs were taken on NanoZoomer 2 OHT (Hamamatsu Photonics GmbH, Herrsching am Ammersee, Germany). Overview photographs depicting general liver morphology (scale bar: 5 mm) are shown on the left. Framed pictures on the right side represent higher magnifications (scale bar: 250 µm). Portal tracts (arrowheads), central veins (CV), and dysplastic nodules (N) are denoted. Asterisks indicate areas with subcapsular fibrosis and ductular reaction with atrophy of liver parenchyma.

To investigate the *Nf2*-dependent histopathological changes, livers isolated from *Nf2^flox/flox^*;*Alb-Cre* and control animals were subjected to conventional histology as well as immunohistochemistry. The histopathological analyses revealed extensive ductular proliferation of bile ducts in the portal tracts of *Nf2^flox/flox^*;*Alb-Cre* animals, possibly due to proliferation of the putative liver progenitor cells, so-called oval cells (Figure 2, Figures S2 and S3). Morphology of hepatocytes remained unchanged (Figure 2, Figures S2 and S3). 

Moderate ductular reaction in the portal tract areas was already found in 2- and 6-week-old *Nf2*-mutant mice and progressed in the 20- to 32-week-old cohort, ultimately leading to a massive tumor-like ductular process radiating from and bridging portal areas in livers of 48-week-old *Nf2*-mutant mice (Figure 2 and Figure S2). As revealed by Gomori silver staining, livers of 48-week-old mice exhibited progressive fibrosis, visible as bridges of collagen that connected individual portal tracts and entrapped islands of hepatocytes, with pseudolobule formation and distortion of liver architecture, indicative of cirrhosis (Figure S3). Comparable pathological changes were observed in livers isolated from *Cd44^+/+^*;*Nf2^flox/flox^*;*Alb-Cre*, *Cd44^−/−^*;*Nf2^flox/flox^*;*Alb-Cre,* and *Cd44^flox/flox^*;*Nf2^flox/flox^*;*Alb-Cre* mice (Figure 2, Figures S2 and S3), suggesting that the status of the *Cd44* gene has no influence on ductular proliferation or liver fibrosis.

**Figure 3:**
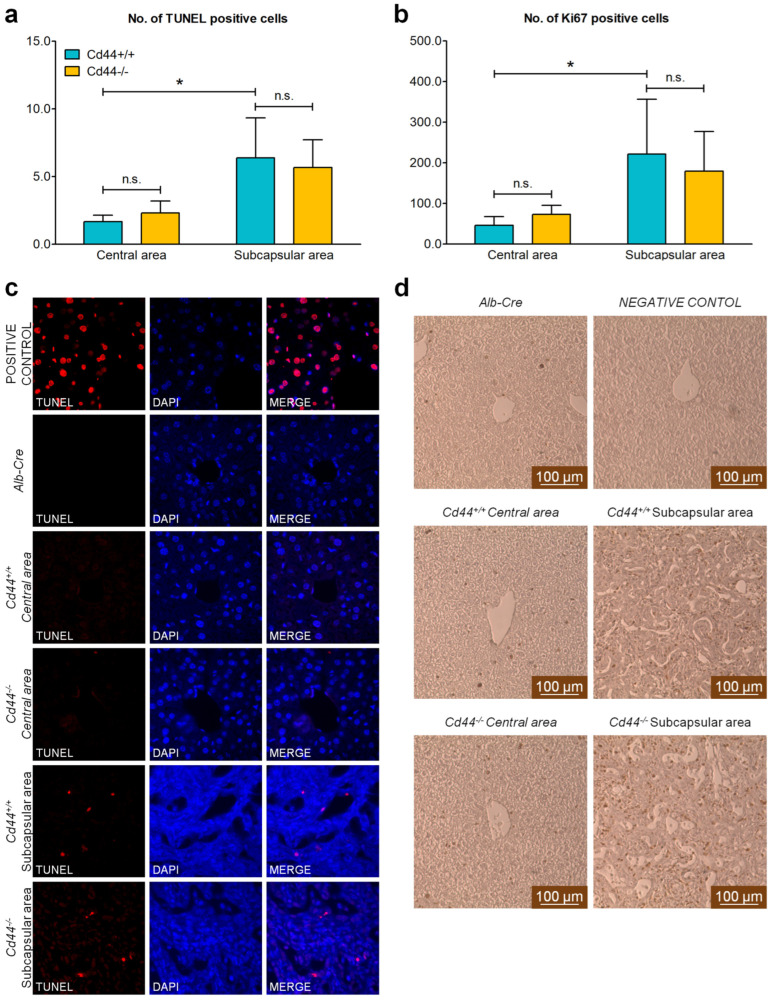
In situ detection of apoptosis and proliferation in livers from 6-week-old *Nf2*-mutant mice. (**a**,**c**) Apoptosis was detected in livers isolated from 6-week-old *Alb-Cre*, *Cd44^+/+^*;*Nf2^flox/flox^*;*Alb-Cre* and *Cd44^−/−^*;*Nf2^flox/flox^*;*Alb-Cre* mice using TUNEL assay. *Alb-Cre* livers treated with DNase I served as a positive control. Fluorescent photographs were generated with an ApoTome Axiovert 200 microscope (Carl Zeiss Meditec AG, Jena, Germany) with 40× magnification. The number of TUNEL-positive cells (Alexa Fluor 594—red) was calculated in six magnification fields (40×) for four independent *Cd44^+/+^*;*Nf2^flox/flox^*;*Alb-Cre* (turquoise) and three independent *Cd44^−/−^*;*Nf2^flox/flox^*;*Alb-Cre* (yellow) livers. The bar chart in (**a**) shows mean number of TUNEL-positive cells ±SD per one magnification filed (40×). Representative pictures taken for central and subcapsular areas of the livers are shown in (**c**). All three analyzed *Alb-Cre* control livers were negative for TUNEL staining. (**b**,**d**) Ki67 was detected as a marker of proliferation in livers isolated from 6-week-old *Alb-Cre*, *Cd44^+/+^*;*Nf2^flox/flox^*;*Alb-Cre* and *Cd44^−/−^*;*Nf2^flox/flox^*;*Alb-Cre* mice by immunohistochemistry. For negative control, primary antibodies were omitted. Photographs were taken using a Leica DFC290 microscope with 20× magnification. The number of Ki67-positive nuclei was calculated in four magnification fields (20×) for four independent *Cd44^+/+^*;*Nf2^flox/flox^*;*Alb-Cre* (turquoise) and three independent *Cd44^−/−^*;*Nf2^flox/flox^*;*Alb-Cre* (yellow) livers using ImageJ software. Ki67 staining was nearly undetectable in the three analyzed control livers *(Alb-Cre*). The bar chart in (**b**) shows mean number of Ki67-positive cells ±SD per one magnification filed (20×). Representative pictures taken for central and subcapsular areas of livers are shown in (**d**). Scale bar: 100 µm. Student’s *t*-test two-tailed values: n.s.: *p* > 0.05, *: *p* ≤ 0.05.

In younger mice, ductular reaction and fibrosis were most pronounced in subcapsular areas (Figure 2 and Figure S2, asterisks). These regions showed increased cell turnover when compared to central regions containing hepatocytes (Figure 3). Both the rate of cell death (Figure 3a,c, measured by TUNEL assay) and cell proliferation (Figure 3b,d, indicated by positive Ki67 staining) were increased three- to four-fold in subcapsular areas when compared to central areas. 

There were no significant differences in cell proliferation or apoptosis between *Cd44*-positive and *Cd44*-negative *Nf2*-mutant livers (Figure 3).

### 3.3. CD44 Exhibits Similar Expression Pattern to Ductular/Liver Progenitor Cell Markers, but Is Not Required for Ductular Process in Nf2^flox/flox^;Alb-Cre Mice

**Figure 4:**
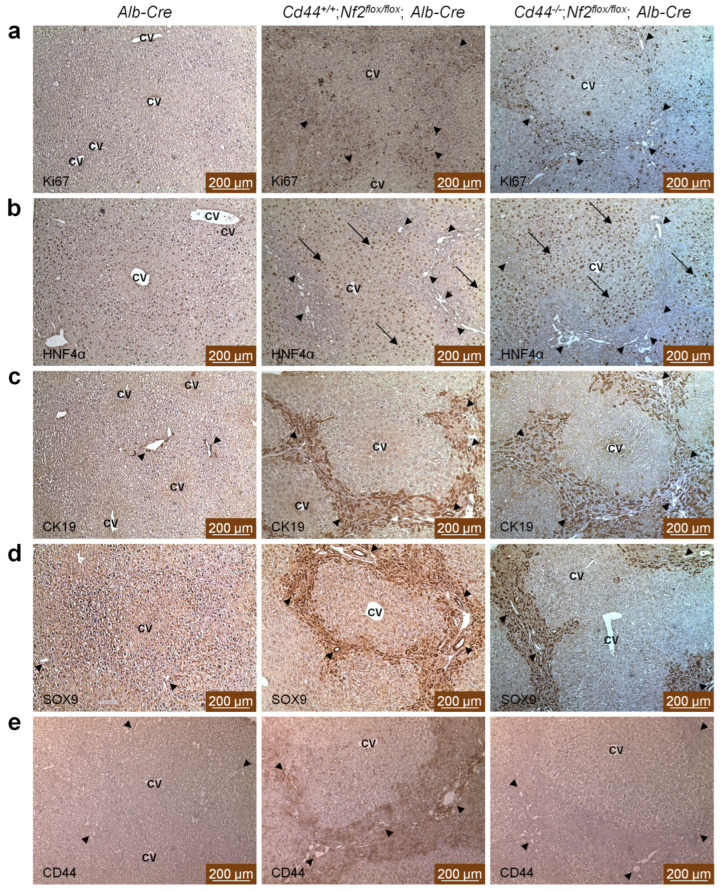
Immunohistochemical staining of livers from 32-week-old *Nf2*-mutant mice. (**a**) Ki67 staining revealed the presence of ductular reactions in portal tracts of *Nf2^flox/flox^*;*Alb-Cre* livers. Ki67 staining was almost undetectable in control livers *(Alb-Cre*). (**b**) Immunohistochemical detection of HNF4α as a marker of hepatocytes. (**c**,**d**) Immunohistochemical analysis using an anti-CK19 and anti-SOX9 antibody revealed prominent and progressive staining in the tumor-like ductular proliferations of livers isolated from *Cd44^+/+^*;*Nf2^flox/flox^*;*Alb-Crem* and *Cd44^−/−^*;*Nf2^flox/flox^*;*Alb-Cre* mice. In contrast, only a subset of portal bile duct cells expressed CK19 and SOX9 antigens in the control *Alb-Cre* livers. (**e**) Immunohistochemical analysis demonstrated prominent CD44 staining in bile ducts and ductular proliferations of livers isolated from *Cd44^+/+^*;*Nf2^flox/flox^*;*Alb-Cre* mice. CD44 staining was undetectable in *Cd44^−/−^*;*Nf2^flox/flox^*;*Alb-Cre* mice. Central veins (CV), portal tracts (arrowheads) and hepatocytes (arrows) are denoted. Photographs were taken using a Leica DFC290 microscope (Leica Microsystems CMS GmbH, Mannheim, Germany). Scale bar: 200 µm.

To further characterize ductular proliferations observed in *Nf2*-mutant mice, ductular/liver progenitor cell markers cytokeratin 19 (CK19) and transcription factor SOX9 were analyzed. In addition, hepatocyte nuclear factor 4 alpha (HNF4α) was detected to distinguish hepatocytes, and Ki67 staining was performed to identify proliferating cells. At later ages, the ductular process was distributed throughout the portal and periportal areas of *Nf2*-mutant livers. Ki67-staining highlighted an increased rate of proliferation in ductular reactions from *Cd44^+/+^*;*Nf2^flox/flox^*;*Alb-Cre* and *Cd44^−/−^*;*Nf2^flox/flox^*;*Alb-Cre* livers (Figure 4a and Figure S4a). Patches of proliferating ductular reactions radiated from portal tracts, whereas hepatocytes were largely Ki67-negative (Figure 4a and Figure S4a). HNF4α showed positive nuclear staining in hepatocytes but was absent in ductular reactions (Figure 4b and Figure S4b). Instead, tumor-like ductular proliferations were positive for CK19 and SOX9 staining (Figure 4c,d and Figure S4c,d). Figure 4c,d and Figure S4c,d demonstrate the comparably marked CK19 and SOX9 staining of cholangiocytes of ductular reaction/oval cell proliferation highlighting fibrotic septae between portal tracts and the formation of pseudolobuli in *Nf2*-mutant livers; in the *Alb-Cre* control livers, only the originary bile ducts expressed CK19 and SOX9 antigens and no ductular proliferation was noted. Despite some regional differences in intensity and thickness of CK19 and SOX9 -positive cell layer, we did not observe any significant differences between *Cd44^+/+^*;*Nf2^flox/flox^*;*Alb-Cre* and *Cd44^−/−^*;*Nf2^flox/flox^*;*Alb-Cre* livers (Figure 4c,d and Figure S4c,d). 

Furthermore, we investigated localization of CD44 in mutant livers. Immunohistochemical analysis (Figure 4e and Figure S4e) and immunofluorescent staining (Figure S5) revealed that CD44 is expressed in bile ducts and ductular proliferations, but not in hepatocytes (Figure 4e). As expected, *Cd44*^−/−^-livers were negative for CD44 staining (Figure 4e, Figures S4e and S5). Together, our results suggest that despite a similar expression pattern to ductular/liver progenitor cell markers, *Cd44* is not required for ductular proliferation in *Nf2^flox/flox^*;*Alb-Cre* mice.

### 3.4. YAP Signaling Is Elevated in Nf2-Deficient Livers

**Figure 5:**
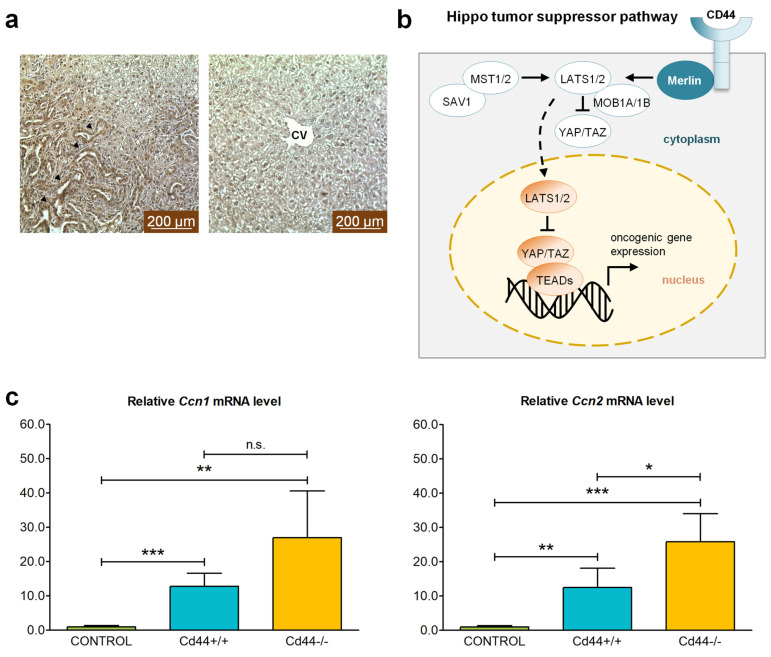
Effect of *Nf2* knockout on YAP signaling in the liver. (**a**) Immunohistochemical localization of YAP in livers from 6-week-old *Nf2*-mutant mice. Prominent YAP staining is detected in ductular reactions in portal tracts of *Nf2^flox/flox^*;*Alb-Cre* livers. In contrast, hepatocytes are largely negative for YAP staining. Central veins (CV) and portal tracts (arrowheads) are denoted. Photographs were taken using a Leica DFC290 microscope. Scale bar: 200 µm. (**b**) Hippo tumor suppressor signaling pathway. Modified from Sato and Sekido, 2018 [24]; Cooper and Giancotti, 2014 [25]; Ma et. al. 2020 [43]. CD44 provides a link between Merlin and the plasma membrane. Merlin directly binds and recruits the LATS1/2 kinase to the plasma membrane, prompting its phosphorylation by the MST1/2 kinase bound to adapter protein SAV1 [50]. In turn, LATS1/2, in a complex with small regulator protein MOB1A/1B, phosphorylates and inactivates YAP/TAZ, blocking their role as TEAD/MEAD transcription factor co-activators [50]. (**c**) Expression of YAP target genes *Ccn1* and *Ccn2* was measured in livers isolated from 48-week-old animals by real-time qRT-PCR. The bar charts show mean mRNA level ±SD in four independent *Cd44^+/+^;Nf2^flox/flox^;Alb-Cre* (turquoise) and *Cd44^−/−^;Nf2^flox/flox^;Alb-Cre* (yellow) liver tumors, relative to the level in control livers (*Alb-Cre*, green). Student’s *t*-test two-tailed values: n.s.: *p* > 0.05, *: *p* ≤ 0.05, **: *p* ≤ 0.01, ***: *p* ≤ 0.001.

NF2/Merlin acts as an upstream regulator of the Hippo signaling tumor suppressor pathway that inhibits oncogenic YAP activity (Figure 5b). Accordingly, *Nf2*-deficient livers exhibited enhanced staining of YAP and increased transcriptional activity of YAP target genes when compared to control mice (Figure 5). YAP was predominantly localized in the ductular reactions of *Nf2^flox/flox^*;*Alb-Cre* mice, whereas hepatocytes were largely negative for YAP staining (Figure 5a). Expression of YAP target genes *Ccn1* and *Ccn2* increased more than tenfold in livers isolated from *Cd44*^+/+^;*Nf2^flox/flox^*;*Alb-Cre* mice compared to those isolated from *Alb-Cre* mice (Figure 5c). Transcriptional activity of YAP was even higher in *Cd44*-negative *Nf2*-mutant livers compared to *Cd44*-positive livers. However, these effects were only significant for the *Ccn2* gene. Increased activity of YAP oncogene is compatible with its role in mediating tumorigenic progression in *Nf2*-mutant livers. The effects of *Cd44* deletion on expression of YAP target genes are difficult to interpret given the high variations in *Ccn1* and *Ccn2* mRNA levels between single *Cd44*-individuals.

### 3.5. Deletion of Cd44 Does Not Influence Primary Liver Tumor Generation but Virtually Aborts Metastasis Formation in Nf2^flox/flox^;Alb-Cre Mice

Furthermore, we investigated the influence of CD44 on liver tumor development and progression in *Nf2*-mutant mice. We observed that nearly all *Nf2^flox/flox^*;*Alb-Cre* mice older than 32 weeks, whether *Cd44*-positive or *Cd44*-negative, developed frank liver tumors, and primarily HCCs. However, transitional cellular and histological features that were intermediate between HCCs and iCCAs were also frequently observed. The former exhibited a striking variety of histopathological types, including macrotrabecular and pseudoglandular morphologies (Figure S6). However, we did not observe any significant differences in the spectrum of tumors detected in *Cd44^+/+^*;*Nf2^flox/flox^*;*Alb-Cre*, *Cd44^−/−^*;*Nf2^flox/flox^*;*Alb-Cre,* and *Cd44^flox/flox^*;*Nf2^flox/flox^*;*Alb-Cre* mice.

**Figure 6:**
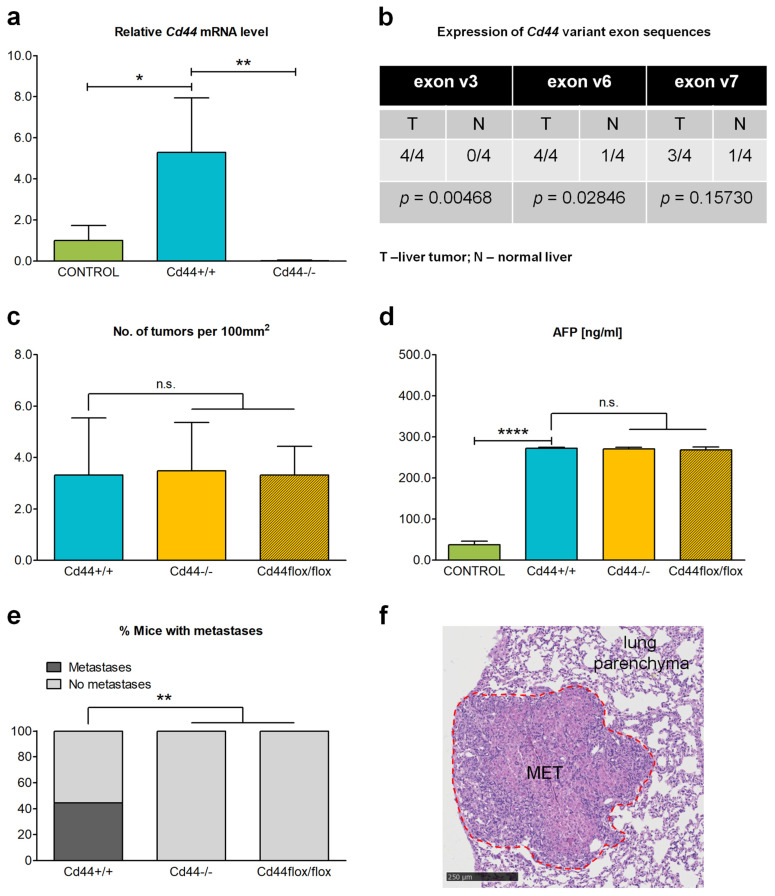
Influence of *Cd44* expression on primary liver tumor development and metastasis formation. (**a**) Total *Cd44* mRNA expression was measured in livers isolated from 48-week-old animals by real-time qRT-PCR using primers to detect all potential *Cd44* isoforms. The bar chart shows mean *Cd44* mRNA level ±SD in four independent *Cd44^+/+^;Nf2^flox/flox^;Alb-Cre* (turquoise) and *Cd44^−/−^;Nf2^flox/flox^;Alb-Cre* (yellow) liver tumors relative to the level in normal livers from four independent *Alb-Cre* (green) mice. Student’s *t*-test two-tailed values: *: *p* ≤ 0.05, **: *p* ≤ 0.01. (**b**) Expression of *Cd44* variant exons v3, v6, and v7 was detected by real-time qRT-PCR. Four liver tumor samples (T) isolated from independent *Cd44^+/+^*;*Nf2^flox/flox^*;*Alb-Cre* mice and four normal livers (N) isolated from *Alb-Cre* control mice were analyzed. Sequences encoded by variant exon v3 were present in all four tumor samples and absent in normal livers. Sequences encoded by exon v6 were present in all four tumor samples and in one out of four normal livers. Sequences encoded by exon v7 were present ©n three out of four tumor samples and in one out of four normal livers. Chi-Square test of independence *p* values for tumor versus normal tissue are indicated. (**c**) Quantification of primary liver tumors in 48-week-old *Nf2^flox/flox^*;*Alb-Cre* mice. The bar chart shows mean number of tumors per 100 mm^2^ of liver specimen ±SD. Nine *Cd44^+/+^*;*Nf2^flox/flox^*;*Alb-Cre* (turquoise), nine *Cd44^−/−^*;*Nf2^flox/flox^*;*Alb-Cre* (yellow) and five *Cd44^flox/flox^*;*Nf2^flox/flox^*;*Alb-Cre* (yellow dashed) mice were analyzed. Student’s *t*-test two-tailed value: n.s.: *p* > 0.05. (**d**) Serum level of AFP in 48-week-old mice. AFP level was measured by immunoassay in serum of four *Alb-Cre* control, seven *Cd44^+/+^*;*Nf2^flox/flox^*;*Alb-Cre*, nine *Cd44^−/−^*;*Nf2^flox/flox^*;*Alb-Cre,* and five *Cd44^flox/flox^*;*Nf2^flox/flox^*;*Alb-Cre* mice. The bar chart shows mean level of AFP (ng/mL) ±SD. Student’s *t*-test two-tailed values: n.s.: *p* > 0.05, ****: *p* ≤ 0.0001. (**e**) Quantification of lung metastases in 48-week-old *Nf2^flox/flox^*;*Alb-Cre* mice. The bar chart shows percentage of mice with (dark grey) or without (light grey) lung metastases. Nine *Cd44^+/+^*;*Nf2^flox/flox^*;*Alb-Cre*, nine *Cd44^−/−^*;*Nf2^flox/flox^*;*Alb-Cre,* and five *Cd44^flox/flox^*;*Nf2^flox/flox^*;*Alb-Cre* mice were analyzed. The significance was determined by a Chi-Square test of independence. **: *p* ≤ 0.01 (**f**) Hematoxylin and eosin (H&E) -stained paraffin lung section with visible mixed HCC-iCCA lung metastasis (MET). The photograph was taken on NanoZoomer 2 OHT. Scale bar 250 µm.

The level of total *Cd44* mRNA was significantly increased in liver tumors isolated from 48-week-old *Cd44^+/+^*;*Nf2^flox/flox^*;*Alb-Cre* mice compared to age-matched control livers isolated from *Alb-Cre* mice (Figure 6a). As expected, *Cd44^−/−^* tumors did not express relevant *Cd44* mRNA levels (Figure 6a). Moreover, nearly all livers isolated from 48-week-old *Cd44^+/+^*;*Nf2^flox/flox^*;*Alb-Cre* mice were positive for expression of exon v3-, v6-, and v7-bearing CD44 variant isoforms that have previously been implicated in cancer (Figure 6b). Livers isolated from *Alb-Cre* mice were applied as controls, as it was not possible to identify normal liver areas in 48-week-old *Nf2^flox/flox^;Alb-Cre* animals given the extensive tumor-like ductular process. The increased expression of total *Cd44* mRNA and presence of *Cd44* variant isoforms, including exon v3, v6, and v7 sequences in liver tumors from *Cd44^+/+^*;*Nf2^flox/flox^*;*Alb-Cre* mice, is compatible with the function of CD44 and its isoforms in the formation of primary liver tumors and/or metastases. However, the *Cd44* gene status had no influence on either the number of primary liver tumors detected or on the level of the hepatocellular carcinoma biomarker AFP (α-fetoprotein) in the serum of *Nf2*-mutant mice (Figure 6c,d). In sum, our results suggest that CD44 does not influence primary liver tumor generation in *Nf2*-mutant mice.

Next, we were interested in the role of CD44 in liver tumor progression. Liver metastases were detected in the lungs of four of the nine analyzed 48-week-old *Cd44^+/+^*;*Nf2^flox/flox^*;*Alb-Cre* mice (Figure 6e). These included two HCCs, one iCCA, and one mixed HCC-iCCA metastasis. In contrast, no metastases were found in nine of the analyzed *Cd44^−/−^*;*Nf2^flox/flox^*;*Alb-Cre* or five of the analyzed *Cd44^flox/flox^*;*Nf2^flox/flox^*;*Alb-Cre* mice. No extrahepatic lesions other than lung metastases were detected. Together, our results indicate that CD44 is not required either for ductular proliferation or for generation of HCCs or the other primary liver tumors observed in *Nf2^flox/flox^*;*Alb-Cre* mice. Instead, we identified a critical role of CD44 in the generation of liver tumor metastases in the lung.

### 3.6. Deletion of the Cd44 Gene Does Not Influence Colony-Forming Ability or Migration into Cell-Free Gap of Nf2-Negative Liver Cells 

**Figure 7:**
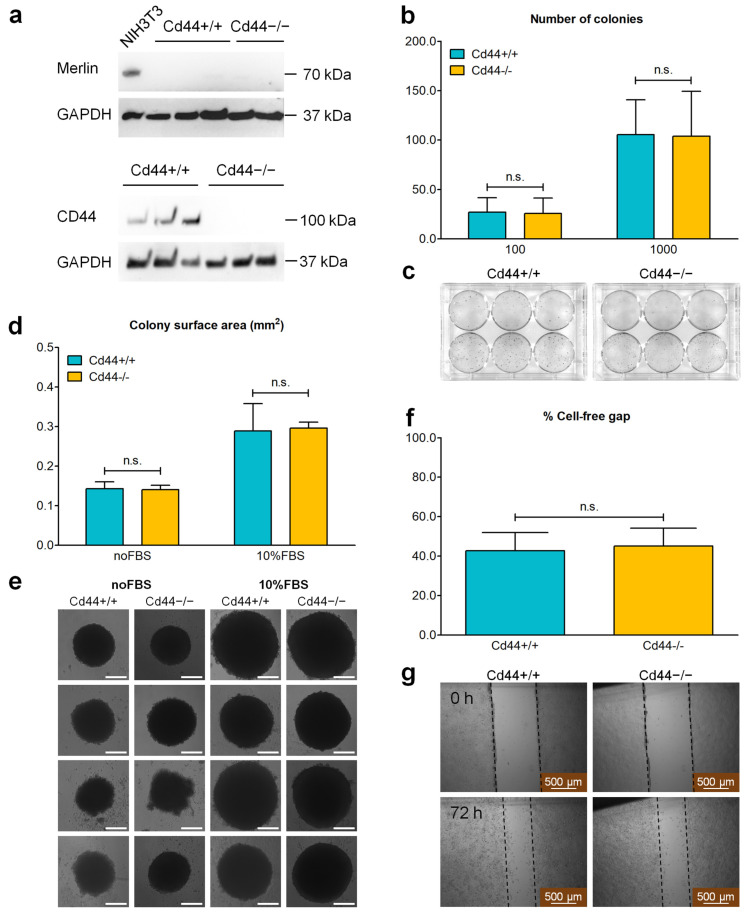
Testing colony-forming ability and migration into cell-free gap of *Nf2*-negative liver cells. (**a**) Detection of Merlin and CD44 in liver cell lines by immunoblot. *Cd44*-positive and *Cd44*-negative liver cell lines were generated from *Cd44^+/+^*;*Nf2^flox/flox^*;*Alb-Cre,* and *Cd44^−/−^*;*Nf2^flox/flox^*;*Alb-Cre* mice. Three independent *Cd44*^+/+^ and two-three independent *Cd44*^−/−^ cell lines were tested. NIH3T3 cells were applied as a positive control for detection of Merlin. The cells were seeded at 50% confluency in DMEM medium supplemented with 10% FBS and left overnight to attach, then subjected to immunoblot. Merlin was detected using anti-Merlin antibody, clone EPR2573(2). CD44 was detected using anti-CD44 antibody, clone E7K2Y. GAPDH was detected to control equal loading of samples. (**b**,**c**) Testing colony-forming ability of *Nf2*-deficient liver cells. Four independent *Cd44*-positive and *Cd44*-negative liver cell lines were seeded in triplicates in 6-well plates at a density of 100 (upper wells) or 1000 (lower wells) cells per well. The bar chart in **(b)** shows mean colony number ±SD formed after 7 days. The initial number of cells seeded (100 or 1000) is indicated on x-axis. Student’s *t*-test two-tailed values: n.s.: *p* > 0.05. (**c**) Representative pictures of colonies. (**d,e**) Testing anchorage-independent growth of liver cells. The bar chart in (**d**) represents mean value of colony surface area ±SD measured for four independent *Cd44*-positive and *Cd44*-negative liver cell lines. At least three technical replicates were completed for each cell line. **(e)** Representative pictures of colonies formed either upon serum starvation (noFBS) or in medium supplemented with 10% FBS. The photographs were taken using a Leica DFC290 microscope. Scale bar: 200 µm. Student’s *t*-test two-tailed value: n.s.: *p* > 0.05. (**f**,**g**) Testing migration of liver cells. Migration into cell-free gap was measured using conventional scratch assay. (**f**) The bar chart shows mean values of % cell-free gap ±SD measured after 72 h of migration for four independent *Cd44*-positive and three independent *Cd44*-negative liver cell lines. At least three technical replicates were completed for each cell line. (**g**) Representative pictures of gaps taken directly after introducing a gap (time 0h) and after 72 h of migration. Photographs were taken using a Leica DFC290 microscope. Scale bar: 500 µm.

Liver tumor cells were isolated from *Nf2*-mutant mice to further investigate the role of CD44 in liver tumor development and progression. Subsequently, the cells isolated from *Cd44^+/+^*;*Nf2^flox/flox^*;*Alb-Cre,* and *Cd44^−/−^*;*Nf2^flox/flox^*;*Alb-Cre* livers will be identified as *Cd44^+/+^* and *Cd44^−/−^*, respectively. As expected, all cell lines generated from *Nf2^flox/flox^*;*Alb-Cre* livers were negative for NF2/Merlin expression (Figure 7a). Because *Alb-Cre*-dependent recombination of *Nf2 flox* alleles is restricted to liver cells, these results indicate a lack of contamination from other cell types. Through immunoblot analysis, we also confirmed that only *Cd44^+/+^* cells, and not *Cd44^−/−^* cells, were positive for CD44 (Figure 7a). The isolated cells also did not express hepatocyte marker HNF4α, but they were positive for ductular/liver progenitor cell markers CK19 and SOX9 (Figure S7). Although there were cell line-dependent variations in CK19 and SOX9 expression levels, we observed no significant differences in the expression and subcellular localization of CK19 and SOX9 between *Cd44*-positive and *Cd44*-negative cell lines (Figure S7b–d). Similarly, levels of phosphorylated LATS and YAP were highly variable between analyzed cell lines; however, these differences were independent of the *Cd44* gene status, suggesting that, in the absence of Merlin, CD44 has no influence on Hippo signaling (Figure S8).

After confirming that the isolated cells exhibit expected expression pattern of *Nf2*, *Cd44,* and characteristic liver markers, further functional assays were conducted. At first, the self-renewing capability of *Cd44*-positive and *Cd44*-negative cells derived from *Nf2^flox/flox^;Alb-Cre* livers was compared in a clonogenic assay. Deletion of the *Cd44* gene did not influence the capability of *Nf2*-negative liver cells to form colonies under adherent (Figure 7b,c) or nonadherent conditions (Figure 7d,e). Liver cells grown on attachment-resistant plates formed one single spherical colony per well of a 96-well plate (Figure 7d,e). The size of generated spherical colonies grown in medium enriched with 10% FBS was larger compared to those generated in serum-deprived medium (Figure 7d,e). However, we did not observe any significant differences between *Cd44*-positive and *Cd44*-negative colonies (Figure 7d,e). Colony-forming assays are indicative of tumor-initiating potential of investigated cells [51]. Thus, our in vitro results suggest that *Cd44^+/+^* and *Cd44^−/−^* cells do not differ in their tumor-initiating capability. This observation is compatible with our in vivo results demonstrating no difference in tumor development or growth between *Cd44^+/+^*;*Nf2^flox/flox^*;*Alb-Cre* and *Cd44^−/−^*;*Nf2^flox/flox^*;*Alb-Cre* mice (Figure 6c,d). 

Attachment-independent growth is also a prerequisite for metastatic spread of tumor cells. A large percentage of tumor cells that enter the bloodstream or the lymph during metastatic dissemination die due to loss of cell–cell and cell–matrix contacts, undergoing a specific type of apoptosis known as “anoikis” [52]. Our results suggest that CD44 does not influence the ability of liver cells to evade anoikis (Figure 7d,e). Migration is another prerequisite for metastasis formation. To test the influence of CD44 on cell migration in vitro, a conventional scratch migration assay was conducted. CD44 had no influence on the ability of isolated liver cells on migration into an introduced cell-free gap (Figure 7f,g). Thus, the inhibitory effect of the *Cd44* gene deletion on metastasis formation in vivo (Figure 6e,f) cannot be explained by the influence on anoikis or simple migration.

### 3.7. CD44 Contributes to Transendothelial Migration of Nf2-Negative Liver Cells by Upregulating Levels of Integrin Subunit Beta 2

**Figure 8:**
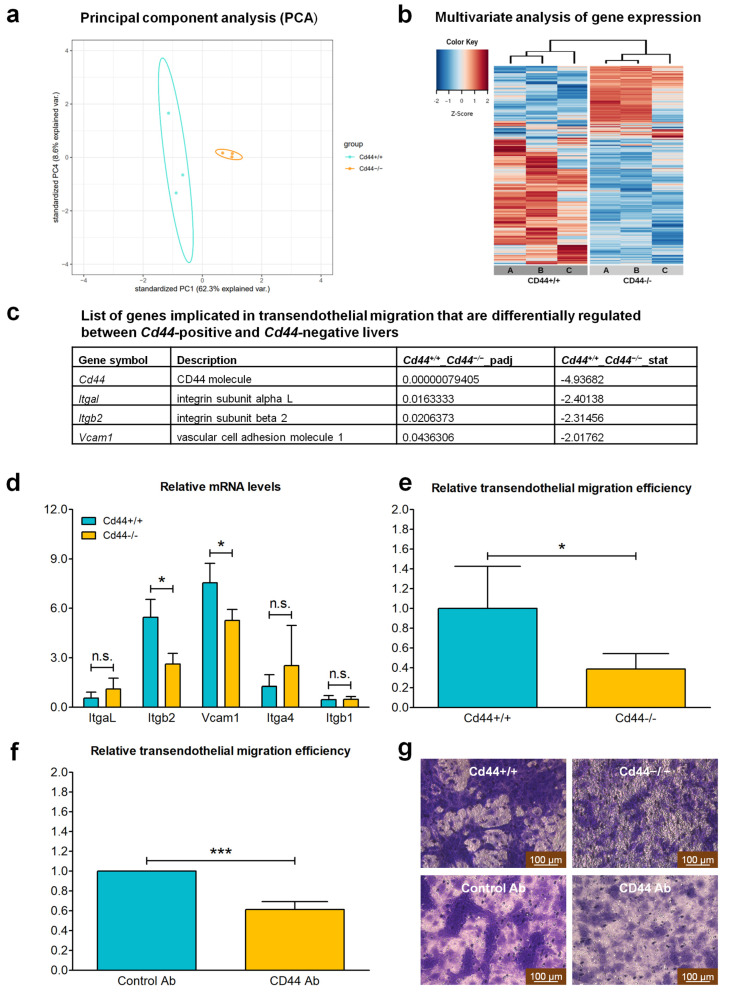
Influence of CD44 on integrin expression and transendothelial migration. (**a**,**b**) RNA-sequencing analysis. RNA-sequencing was performed on *Cd44*-positive and *Cd44*-negative livers isolated from *Nf2*-mutant mice described previously [43]. (**a**) Principal component analysis (PCA) of RNA sequencing data. Data were plotted along the first and fourth principal components. Each dot in PCA plot indicates a single sample. (**b**) Multivariate analysis of gene expression between *Cd44*-positive and *Cd44*-negative livers. Unsupervised hierarchical clustering and heatmap showing *Cd44^+/+^* and *Cd44^−/−^* livers clearly segregated from one another based on their RNA expression profiles. Three samples of each group were analyzed. Both downregulated (blue) and upregulated (red) RNAs were identified in *Cd44*^−/−^ livers. The analysis was performed using an MADE4 package of R software. (**c**) List of genes implicated in transendothelial migration that are differentially regulated between *Cd44*-positive and *Cd44*-negative livers. The data are represented as Wald-statistic (stat). Genes with negative Wald-statistic values are downregulated in *Cd44^−/−^* compared to *Cd44^+/+^* livers, and genes with positive values are upregulated. Benjamin Hochberg adjusted *p*-value (padj) is indicated. Gene description source: HGNC (www.genenames.org/; accessed on 21 February 2023). (**d**) Relative *ItgaL*, *Itgb2*, *Vcam1, Itga4* and *Itgb1* mRNA levels in *Cd44*-positive and *Cd44*-negative livers isolated from 48-week-old *Nf2^flox/flox^*;*Alb-Cre* mice. The bar chart shows mean mRNA level ±SD from three independent *Cd44^+/+^*;*Nf2^flox/flox^*;*Alb-Cre* (turquoise) and *Cd44^−/−^*; *Nf2^flox/flox^*;*Alb-Cre* (yellow) livers. Y-axis shows mean mRNA level relative to *Alb-Cre* control livers (three independent samples). X-axis indicates analyzed genes. (**e**–**g**) Testing transendothelial migration of *N2*-deficient liver cells. Liver cells were allowed to migrate through confluent monolayer of endothelial cells for 22 h. Where indicated, liver cells were preincubated with CD44 blocking antibody (Ab), or isotype-matched control antibody at room temperature for 15 min prior to migration and migration performed in the presence of indicated antibodies (**e**) Bar chart shows mean value of relative transmigration ±SD, measured in triplicates for four independent *Cd44*-positive and *Cd44*-negative liver cell lines. (**f**) Bar chart shows mean value of relative transmigration ±SD, measured in triplicates and in three independent experiments. (**g**) Representative pictures of transmigrating liver cells stained blue. Photographs were taken using a Leica DFC290 microscope. Scale bar: 100 µm. Student’s *t*-test two-tailed values: n.s.: *p* > 0.05, *: *p* ≤ 0.05, ***: *p* ≤ 0.001.

An important hint about the potential mechanism underlying CD44-dependent metastasis formation was provided by our previous study [43]. We performed RNA-sequencing analysis on *Cd44*-positive and *Cd44*-negative livers isolated from *Nf2*-heterozygous mice (Figure 8a,b). We identified that several genes involved in transendothelial migration were among the top-ranked genes differentially regulated between *Cd44*-positive and *Cd44*-negative livers (Figure 8c). The full list of differentially regulated genes can be found in Table S1. Adhesion of tumor cells to endothelial cells and extravasation are important rate-limiting steps in the metastatic cascade. Integrin αLβ2 (LFA-1, lymphocyte function-associated antigen 1) and integrin α4β1 (VLA-4, very late antigen-4) are two major integrins implicated in transendothelial migration of leukocytes and tumor cells [53,54]. Interestingly, expression of genes encoding integrin subunit alpha L (ItgaL) and integrin subunit beta 2 (Itgb2), as well as the substrate of integrin α4β1, VCAM–1 (vascular cell adhesion molecule-1), were significantly downregulated in *Cd44*-negative livers compared to *Cd44*-positive counterparts (Figure 8c). These results strongly indicate a role of CD44 in regulating the major effectors of transendothelial migration of tumor cells.

Real-time qRT-PCR facilitated confirmation that the livers isolated from *Cd44^+/+^*;*Nf2^flox/flox^*;*Alb-Cre* animals express significantly higher levels of *Itgb2* and *Vcam1*, compared to *Cd44^−/−^*; *Nf2^flox/flox^*;*Alb-Cre* livers, whereas the levels of mRNAs of *ItgaL*, *Itga4* and *Itgb1* were not significantly different (Figure 8d). These results are consistent with the RNA sequencing data, although there is a lack of differences in *ItgaL* expression levels between *Cd44^+/+^*;*Nf2^flox/flox^*;*Alb-Cre* and *Cd44^−/−^*; *Nf2^flox/flox^*;*Alb-Cre* animals. The observed discrepancy could depend upon the presence of frank liver tumors in *Nf2^flox/flox^*;*Alb-Cre* mice, but not in the analyzed *Nf2*-heterozygous mice. For instance, it is possible that both *Cd44*-positive and *Cd44*-negative tumors generate high levels of *ItgaL,* and in this case, the level of *Itgb2* would be the limiting factor in the generation of functional integrin αLβ2 (LFA-1). 

Liver cells isolated from *Cd44^−/−^*;*Nf2^flox/flox^*;*Alb-Cre* animals showed significantly reduced efficiency of transendothelial migration in vitro compared with those isolated from *Cd44^+/+^*;*Nf2^flox/flox^*;*Alb-Cre* mice (Figure 8e,g). Moreover, CD44-specific antibody significantly blocked transendothelial migration of *Cd44*-positive liver cells (Figure 8f,g). These results indicate that CD44 may promote metastatic dissemination of liver tumor cells by facilitating transendothelial migration through upregulation of *Itgb2* and *Vcam1* expression.

### 3.8. ICAM-1 May Overtake CD44 Function as a Coreceptor of Receptor Tyrosine Kinase MET in Cd44-Negative Liver Cells 

Considering that Merlin inhibits putative tumor-promoting functions of CD44 (as discussed in the introduction), we expected that deletion of *Nf2* would lead to more a severe phenotype in *Cd44*-positive mice when compared to *Cd44*-negative equivalents. This proved not to be the case; the *Cd44* gene status had no influence on the development of primary liver tumors in *Nf2^flox/flox^*;*Alb-Cre* mice, suggesting that deletion of *Nf2* leads to tumor-promoting mechanisms independent of *Cd44*. Alternatively, we considered that compensatory molecules may overtake CD44 function in *Cd44*-knockout mice. It has been demonstrated that ICAM-1 overtakes CD44 function as a coreceptor of receptor tyrosine kinase MET during liver regeneration [55]. 

**Figure 9:**
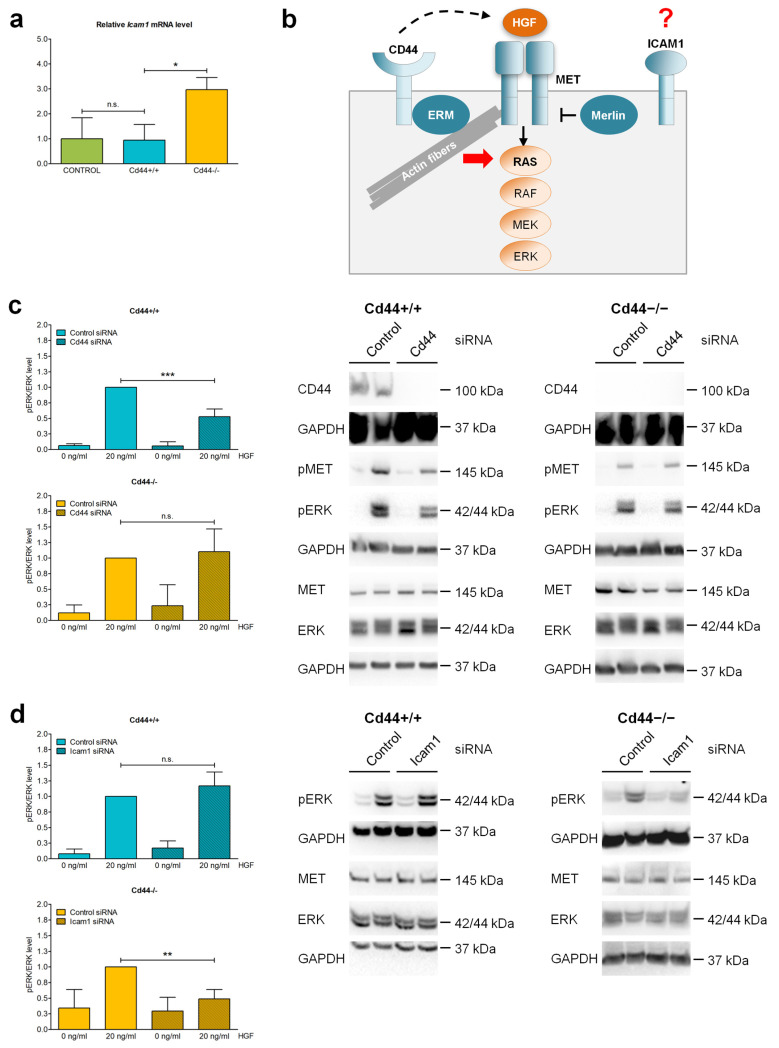
Testing the effect of *Icam1* knockdown on MET-ERK signaling in *Cd44*-negative liver cells. (**a**) *Icam1* mRNA levels in livers isolated from 48-week-old mice. The bar chart shows mean *Icam1* mRNA level ±SD from three independent *Cd44^+/+^*;*Nf2^flox/flox^*;*Alb-Cre* (turquoise) and *Cd44^−/−^*;*Nf2^flox/flox^*;*Alb-Cre* (yellow) livers relative to three independent *Alb-Cre* control (green) livers. (**b**) *Cd44v6*-bearing isoforms act as coreceptor for receptor tyrosine kinase MET, based on Orian-Rousseau et al., 2002 [38] and Morrison et al., 2001 [27]. CD44 participates in presenting HGF to receptor tyrosine kinase MET and is required for its activation. In addition, signal transduction from activated MET to MEK and ERK requires binding of CD44 to ERM proteins. In growth-inhibitory conditions, Merlin replaces ERM proteins bound to CD44, disrupts the link to the actin cytoskeleton, and blocks RAS signaling. (**c**,**d**) Influence of *Cd44* and *Icam1* downregulation on MET-ERK signaling in *Cd44*-positive and *Cd44*-negative liver cell lines. *Cd44*-positive and *Cd44*-negative liver cell lines were generated from *Cd44^+/+^*;*Nf2^flox/flox^*;*Alb-Cre* and *Cd44^−/−^*;*Nf2^flox/flox^*;*Alb-Cre* mice. Cells were seeded at 20–30% confluency in DMEM medium supplemented with 10% FBS. siRNA transfections were performed as described in the Materials and Methods section. Next, 48 h after transfection, the cells were serum starved for 16 h, then incubated with 20 ng/mL HGF (R&D Systems, Inc., Minneapolis, Minnesota, USA) in DMEM without serum and at 37 °C for 5 min. Immunoblots in (**c**,**d**) show detection of total and phosphorylated ERK1/2 and MET proteins. The efficiency of *Cd44* knockdown was controlled by immunoblotting using antibody, clone E7K2Y. The average efficiency of *Cd44* knockdown measured in *Cd44*-positive cells was 97.3% (SD: ± 0.9). Because the ICAM-1 antibody did not work for Western Blot, the efficiency of *Icam1* knockdown was calculated by real-time qRT-PCR. The average efficiency of *Icam1* knockdown was 81.22% (SD: ± 12.08) in *Cd44+/+* cell lines and 79.23% (SD: ± 11.21) in *Cd44−/−* cell lines. GAPDH was detected to control equal loading of samples. The bar charts in (**c**,**d**) show relative pERK/ERK levels normalized to GAPDH ±SD from four (**c**)**,** or three (**d**)**,** independent *Cd44^+/+^* and three independent *Cd44^−/−^* cell lines. Student’s *t*-test two-tailed values: n.s.: *p* > 0.05, *: *p* ≤ 0.05, **: *p* ≤ 0.01, ***: *p* ≤ 0.001.

To test the possibility that ICAM-1 may compensate for CD44 loss during tumorigenesis in *Nf2^flox/flox^*;*Alb-Cre* mice, we first investigated *Icam1* expression in the livers of *Nf2^flox/flox^*;*Alb-Cre* mice. Interestingly, *Cd44^−/−^* livers showed significantly increased levels of *Icam1* mRNA compared to *Cd44^+/+^* livers, suggesting a compensatory mechanism (Figure 9a). Next, we tested the effect of *Cd44* and *Icam1* downregulation on HGF/MET signaling in liver cell lines isolated from *Nf2*-mutant mice. *Cd44* and *Icam1* expression was downregulated using small interfering RNA (siRNA). As readout for HGF/MET signaling, phosphorylated ERK was detected (see also diagram in Figure 9b). The levels of phosphorylated MET and ERK were strongly elevated in liver cells treated with 20 ng/mL of HGF (Figure 9c,d). In *Cd44^+/+^* liver cells, the activation of MET was strictly dependent on CD44 as the levels of phosphorylated ERK were inhibited by *Cd44*-knockdown (Figure 9c). As expected, siRNA targeting *Cd44* had no influence on MET-ERK signaling in *Cd44*-negative liver cells (Figure 9c). Interestingly, *Icam1* knockdown inhibited ERK phosphorylation only in *Cd44*-negative liver cells, whereas it had no effect on MET/ERK signaling in *Cd44*-positive liver cells (Figure 9d). These results suggest that CD44 is sufficient for MET activation in the absence of ICAM-1. Nevertheless, ICAM-1 can overtake CD44 function as a coreceptor of MET in *Cd44*-knockout cells. This could explain why *Cd44* knockout in the liver of *Nf2^flox/flox^*;*Alb-Cre* mice led to milder effects on tumor development and growth than expected based on previous reports [32,33,34,35,36].

## 4. Discussion

### 4.1. Deletion of Nf2 in the Liver Leads to CD44-Independent Liver Enlargement

This study analyzed the influence of CD44 on the phenotype of mice with a *Nf2* deletion in the liver. To study the relative contribution of CD44- and Merlin-dependent signaling pathways on liver tumor development and progression, *Cd44*-deficient mice were crossed with *Nf2^flox/flox^*;*Alb-Cre* mice. Our results demonstrate that deletion of the *Cd44* gene had no effect on liver size or primary tumor development in *Nf2*-mutant mice. Instead, CD44 was required for metastatic spreading of *Nf2*-deficient liver tumors. CD44 facilitates transendothelial migration of liver cancer cells by promoting expression of integrin subunit beta 2 (*Itgb2*). Moreover, our results indicate that ICAM-1 may substitute for CD44 function as a coreceptor of receptor tyrosine kinase MET, and thus may compensate for CD44 loss during other tumor-relevant processes in *Nf2*-deficient livers.

*Nf2^flox/flox^;Alb-Cre* mice showed progressive liver enlargement and developed HCCs, iCCAs, and tumors of mixed histology, often within the same liver (Figure 1). These results are consistent with previous reports demonstrating that deletion of the *Nf2* gene in the liver [5,6]—or genetic inactivation of other members of the Hippo signaling pathway *Mst1/2* [7,8,9], *Lats1/2* [10,11], *Mob1a/b* [12], or *Sav1* [9,13]—leads to liver overgrowth and promotes tumorigenesis. Nevertheless, the phenotype of our *Nf2^flox/flox^;Alb-Cre* mice was milder overall than previously reported [5,6]. In contrast to Benhamouche et al. [6], we did not observe any significant effects of *Nf2*-deletion on liver function or animal survival. The penetrance of *Nf2*-deficient liver phenotype could possibly be influenced by the genetic background of applied mice; C57BL/6 in our study, FVB/N in the study of Benhamouche et al. [6], and not specified in the study of Zhang et al. [5]. Association between genetic background and penetrance of a phenotype of genetically engineered mice is very well documented [56,57]. Other factors, including the strategy to impair Merlin function, were exactly the same in our study and in those of Benhamouche et al. [6] and Zhang et al. [5]; thus, the recombination efficiency of *Nf2* flox alleles should also be comparable.

### 4.2. Origin of Active Cells during Ductular Reaction Observed in Nf2^flox/flox^;Alb-Cre Mice

The liver overgrowth observed in *Nf2^flox/flox^;Alb-Cre* mice was due to ductular proliferation of bile ducts in the portal tracts. We observed the appearance of small overproliferating cells that were positive for ductular/liver progenitor cell markers CK19 and SOX9 (Figure 4c,d and Figure S4c,d); however, the origin of these cells is unclear. The origin of active cells during ductular reaction could be cholangiocytes, hepatocytes, or hepatic progenitor cells [58]. The albumin promoter becomes active in embryonic hepatoblasts that give rise to both hepatocytes and cholangiocytes, and thus, recombination of floxed alleles in mice carrying the *Alb-Cre* transgene is expected to affect all major types of liver cells [6,46,47]. Previous studies reached contradictory conclusions about the origin of proliferating cells appearing in the livers of *Nf2^flox/flox^;Alb-Cre* mice. Based on the positive anti-pan-cytokeratin, A6, and anti-CD34 staining and morphological features characteristic for oval cells, Benhamouche et al. [6] concluded that overproliferating cells appearing in *Nf2*-deficient livers originated from putative liver progenitor cells. To support this hypothesis, they generated *Nf2*-deficient embryonic liver progenitor cells (hepatoblasts) and showed that, upon injection into the livers of immunocompromised mice, multiple foci of proliferating undifferentiated cells were generated. These cells progressed within 3–4 weeks to form larger neoplasia that exhibited both cholangiocytic and hepatocytic features [6]. In contrast, the injected control *Nf2*-positive hepatoblasts were scattered uniformly throughout the liver and did not form neoplasms [6]. Benhamouche et al. [6] concluded that disruption of the *Nf2* gene is sufficient to affect a primary expansion of both embryonic and adult liver progenitor cells in vivo and to reproducibly yield both major forms of primary liver cancer. By employing a combination of lineage tracing and clonal analysis, Yimlamai et al. [59] reached a different conclusion. They discovered that deletion of *Nf2* or overexpression of active YAP form (S127A), specifically in adult hepatocytes, using AAV-Cre, results in their dedifferentiation, driving liver overgrowth and appearance of ductal cells bearing characteristics of hepatic progenitors [59]. Overexpression of active YAP form in the biliary/progenitor compartment, on the other hand, led to hyperplasia, but did not result in an oval-cell-like appearance [59]. While Benhamouche et al. [6] interpreted their findings as liver progenitor-cell expansion and transformation driven by loss of Hippo signaling, those of Yimlamai et al. [59] suggest that hepatocytes might be the source of active cells during the ductular reaction found in *Nf2^flox/flox^*;*Alb-Cre* mice. Considering the findings of Yimlamai et al. [59] and the fact that hepatocytes continue to express *Alb-Cre* postnatally, it is plausible to assume that the overproliferating cells observed in *Nf2*-deficient livers originate from dedifferentiated hepatocytes [6,46,47]. Yet the question remains regarding how *Nf2* deficiency induces dedifferentiation of some hepatocytes, leaving remaining hepatocytes unchanged. We observed that, despite *Nf2* deficiency, the livers of *Nf2^flox/flox^*;*Alb-Cre* mice also contained differentiated hepatocytes that did not exhibit gross morphological alterations or aberrant Ki67 expression in vivo. A slight increase in apoptosis was observed in subcapsular areas, consistent with atrophy of liver parenchyma in these regions (Figure 3a,c). Moreover, Benhamouche et al. [6] observed that *Nf2* deletion in adult mouse liver leads to very mild periporal hyperplasia, compared to dramatic and widespread ductular proliferation in *Alb-Cre*-mediated *Nf2* deletion [6]. Therefore, deletion of *Nf2* alone is not sufficient to reprogram liver cells. They showed that additional proliferative stimuli in the form of partial hepatectomy were required to induce emergence of overproliferating progenitor-like cells and tumor formation in mice with postnatal *Nf2* deletion [6]. Importantly, liver regeneration occurred normally in the absence of Merlin, indicating that, even after receiving a proliferative stimulus and undergoing cell division, *Nf2*^−/−^ hepatocytes can appropriately re-enter a quiescent state [6]. Thus, the exact interplay between signaling components that determine hepatocyte dedifferentiation and acquisition of mature phenotype remains to be determined.

### 4.3. CD44 Does Not Influence Primary Liver Tumor Development in Nf2^flox/flox^;Alb-Cre Mice

To mediate its major tumor suppressor functions, Merlin must be bound to the plasma membrane. This association occurs indirectly through interactions with plasma membrane proteins, including CD44, as the best characterized example [27,60]. Accordingly, numerous *NF2* mutations in cancer patients are predicted to perturb the interaction of Merlin with CD44 [26] and anticipated to interfere with the tumor suppressor activity of Merlin [23]. We hypothesized that disruption of the Merlin-CD44 complex, through loss of Merlin, may unleash putative tumor- or metastasis-promoting functions of CD44. Accordingly, *Cd44* expression was elevated in liver tumors of *Nf2^flox/flox^*;*Alb-Cre* mice when compared to control livers (Figure 6a), which is compatible with CD44 function in liver tumorigenesis. However, the status of *Cd44* gene had no effect on primary liver tumor development in *Nf2^flox/flox^*;*Alb-Cre* mice (Figure 6c,d). Likewise, the ductular proliferation (Figure 2, Figure 3 and Figure 4) and liver size (Figure 1a) were unaffected by *Cd44* loss. In contrast to our study, previous reports demonstrated that deletion of the *Cd44* gene inhibits initiation of colon carcinoma in Apc(Min/+) mice [61] and oncogenic progression in the testis of RHAMM-deficient mice [62]. Thus, our results suggest that deletion of *Nf2* may specifically release tumor-promoting mechanisms that are independent of CD44; or alternatively, CD44 function is compensated by other molecules in *Cd44*-deficient *Nf2*-mutant mice.

As reported elsewhere, these alternative signaling pathways which mediate the phenotype of *Nf2^flox/flox^*;*Alb-Cre* mice may include epidermal growth factor receptor (EGFR), RAC1 and YAP signaling. Benhamouche et al. [6] demonstrated that the phenotype of *Nf2*-mutant livers was effectively suppressed by treating the *Nf2*-deficient mice with erlotinib, an inhibitor of EGFR kinases [6], whereas Zhang et. al. [5] demonstrated that *Nf2*-mutant phenotype was suppressed by heterozygous inactivation of *Yap*. The suppression of *Nf2*-mutant phenotype by loss of *Yap* was specific, since *Yap* deficiency did not suppress liver overgrowth and tumorigenesis induced by the expression of an oncogenic KRAS mutant (G12D) [5]. Moreover, deletion of *Rac1* was shown to block tumor initiation but paradoxically exacerbate hepatomegaly induced by *Nf2* loss [63]. Liver-specific knockout of angiomotin (Amot), which functions upstream of both RAC1 and YAP [64], also effectively suppressed the *Nf2*- mutant phenotype [64]. Despite previous indications that CD44 promotes EGFR [65,66,67] and RAC1 [68,69] signaling and attenuates activation of the Hippo pathway [70,71,72,73,74] in vitro, *Cd44* knockout had no effect on the phenotype of *Nf2*-mutant livers. As such, our results suggest that CD44 does not regulate the above-mentioned pathways in *Nf2*-deficient livers. Consistently, knockout of *Cd44* did not prevent YAP activation in *Nf2*-deficient livers and cells (Figure 5 and Figure S8). Paradoxically, transcriptional activity of YAP was even higher in livers isolated from *Cd44*^−/−^;*Nf2^flox/flox^*;*Alb-Cre* compared to those isolated from *Cd44*^+/+^;*Nf2^flox/flox^*;*Alb-Cre* animals (Figure 5). However, this effect is difficult to interpret because of high variations in mRNA levels of YAP target genes between single *Cd44^−/−^* individuals (Figure 5c). 

### 4.4. Compensatory Molecules May Substitute for CD44 Function during Primary Tumor Development in Cd44-Knockout Nf2^flox/flox^;Alb-Cre Mice

The unexpectedly mild effect of *Cd44* deletion on the phenotype of *Nf2*-deficient livers led us to hypothesize that compensatory molecules may overtake CD44 function during liver tumor development in *Cd44*^−/−^;*Nf2^flox/flox^*;*Alb-Cre* mice. Existence of compensatory molecules was previously proposed to explain the striking discrepancy between in vitro studies, or interfering with CD44 function later during development, and the phenotype of *Cd44^−/−^* mice [48,49]. *Cd44* knockout mice exhibit no overt phenotype during development and only have mild abnormalities in the adult [48,49]. In contrast, interfering with CD44 function later in development, using antisense oligonucleotides or specific CD44 antibodies, led to marked deficits in organ development, skin homeostasis, neuronal axon guidance, numerous immune functions, and haematopoiesis ([75,76]; as reviewed in [37]). These data strongly suggest that CD44 functions can be substituted during early embryogenesis (in knockout mice), whereas at later stages, this is not the case. 

Interestingly, a previous study demonstrated that ICAM-1 can overtake the function of CD44 as a coreceptor of the receptor tyrosine kinase MET during liver regeneration in *Cd44*-null mice [55]. In a number of cellular systems [38,39], or in mice with haploinsufficiency of MET or HGF [77], coactivation of MET was shown to be strictly dependent on the expression of CD44, and specifically, on its isoforms which bear sequences encoded by variant exon v6. Indeed, all tested *Cd44*^+/+^ livers were positive for expression of CD44v6-bearing isoforms (Figure 6b), which is compatible with the role of CD44 as MET coreceptor. Interestingly, livers isolated from *Cd44*^−/−^;*Nf2^flox/flox^*;*Alb-Cre* mice showed elevated levels of mRNA encoding *Icam1* (Figure 9a), suggesting potential compensatory effect. Most strikingly, siRNA-mediated downregulation of *Cd44* inhibited MET activation in *Cd44*^+/+^ liver cells, whereas *Icam1*-specific knockdown interfered with MET-ERK signaling only in *Cd44*^−/−^ liver cells (Figure 9c,d). Our results suggest that, in mice with early obliteration of CD44, receptor tyrosine kinase MET may recruit alternative coreceptor molecules, including ICAM-1 [55]. Not only is MET signaling relevant during liver regeneration; it also plays an important role in liver tumorigenesis [21,58]. Thus, due to expression of compensatory molecules, potential effects of CD44 on liver tumor development or growth may be overseen in *Cd44*-knockout mice. It is possible that interfering with CD44 function later in life will enable identification of additional tumor-relevant CD44 properties. 

### 4.5. CD44 Facilitates Metastatic Dissemination of Nf2-Deficient Liver Tumors 

Our results demonstrate that CD44 is essential for metastatic spreading of *Nf2*-deficient liver tumors (Figure 6e,f). Namely, formation of lung metastasis was observed in 44% of *Cd44*^+/+^;*Nf2^flox/flox^*;*Alb-Cre* animals, but in none of the analyzed *Cd44*-deficient *Nf2*-mutant mice (Figure 6e,f). Of note, one of the earliest reports on *Cd44*-knockout mice demonstrated deficits in migration/homing of myeloid-progenitor cells and lymphocytes [48,49]. Our previous data and other studies suggest that tumor and immune cells might use similar mechanisms to pass from the bloodstream into surrounding tissues and generate metastatic colonies [43,54,78,79]. It has been demonstrated that CD44 regulates expression and activation of integrins VLA-4 and LFA-1 on the surface of leukocytes and tumor cells [43,53,54,78,80,81]. Activated LFA-1 and VLA-4 integrins bind to their respective ligands, ICAM-1 and VCAM-1, on endothelial cells, enabling firm adhesion and passage of migratory cells through endothelium [53,81]. Interestingly, the genes encoding integrin LFA-1 (*Itgal* and *Itgb2*), as well as VCAM-1, were among the top-ranking genes differentially regulated between *Cd44*-positive and *Cd44*-negative livers of *Nf2*-heterozygous mice (Figure 8c). Real-time qRT-PCR results confirmed that expression of *Itgb2* and *Vcam1* is positively regulated by CD44 in *Nf2*-deficient livers (Figure 8d). Moreover, migration of liver cells through a monolayer of endothelial cells was effectively inhibited by *Cd44*-knockout, or by blocking antibodies specific for CD44 (Figure 8e–g). These outcomes suggest that CD44 has an important role in transendothelial migration of liver cells by regulating expression of integrins and their ligands.

## 5. Conclusions

Overall, our results suggest that CD44 facilitates metastatic dissemination of liver tumor cells by upregulating expression of integrin subunit beta 2 and VCAM-1, which are required for transendothelial migration of liver tumor cells. The pro-metastatic function documented in our study could apparently not be substituted by other molecules in *Cd44*-knockout mice. CD44 and integrins might represent a suitable target for interfering with metastatic spreading of liver cancer. Additional CD44 tumor-promoting functions might be revealed when interfering with CD44 function later in the development. 

## Figures and Tables

**Table 1:** List of primary antibodies applied in the study.

Name:	Cat. No.:	Company:	Application: B (Blocking) IF (Immunofluorescence) IHC (Immunohistochemistry) WB (Western Blotting)
CD44, clone E7K2Y	37259	Cell Signaling Technology, Leiden, The Netherlands	IHC 1:100 WB 1:1000
CD44, clone IM7	553131	BD Biosciences, Heidelberg, Germany	B 1:50
CD44, clone KM201	MABT78	Merck KGaA, Darmstadt, Germany	IF 1:50
CK19, clone B-1	sc-374192	Santa Cruz Biotechnology, Inc, Heidelberg, Germany	IF 1:100 IHC 1:200 WB 1:500
GAPDH, clone G-9	365062	Santa Cruz Biotechnology, Inc, Heidelberg, Germany	WB 1:1000
HNF4α, clone K9218	MA1-199	Thermo Fisher Scientific GmbH, Darmstadt, Germany	IF 1:200 IHC 1:200 WB 1:1000
Ki67, clone D3B5	12202	Cell Signaling Technology, Leiden, The Netherlands	IHC 1:200
LATS1, clone C66B5	3477	Cell Signaling Technology, Leiden, The Netherlands	WB 1:1000
Phospho-LATS1(Thr1079), clone D57D3	8654	Cell Signaling Technology, Leiden, The Netherlands	WB 1:1000
MET, clone B2	sc-8057	Santa Cruz Biotechnology, Inc, Heidelberg, Germany	WB 1:500
Phospho-MET	3126	Cell Signaling Technology, Leiden, The Netherlands	WB 1:1000
NF2 / Merlin, clone EPR2573(2)	ab109244	Abcam, Berlin, Germany	WB 1:1000
p44/42 MAPK (ERK1/2), clone L34F12	4696	Cell Signaling Technology, Leiden, The Netherlands	WB 1:1000
Phospho-p44/42 MAPK (ERK1/2) (Thr202/Tyr204)	9101	Cell Signaling Technology, Leiden, The Netherlands	WB 1:1000
SOX9 polyclonal	AB5535	Merck KgaA, Darmstadt, Germany	IF 1:500 IHC 1:200 WB 1:2000
YAP polyclonal	4912	Cell Signaling Technology, Leiden, The Netherlands	IF 1:100 IHC 1:200 WB 1:1000
Phospho-YAP (Ser127) polyclonal	4911	Cell Signaling Technology, Leiden, The Netherlands	WB 1:1000

**Table 2:** List of secondary antibodies applied in the study.

Name:	Cat. No.:	Company:	Application: IF (Immunofluorescence) IHC (Immunohistochemistry) WB (Western Blotting)
Goat anti-Mouse IgG (H+L) Secondary Antibody, HRP	31430	Thermo Fisher Scientific GmbH, Darmstadt, Germany	WB 1:1000
Goat anti-Rabbit IgG F(ab’)2 Secondary Antibody, HRP	31461	Thermo Fisher Scientific GmbH, Darmstadt, Germany	WB 1:1000
Goat anti-mouse (H+L) Secondary Antibody, Alexa Fluor 488	A-11001	Thermo Fisher Scientific GmbH, Darmstadt, Germany	IF 1:1000
Goat anti-Rabbit IgG (H+L) Secondary Antibody, Alexa Fluor 488	A-11034	Thermo Fisher Scientific GmbH, Darmstadt, Germany	IF: 1:500
Goat anti-Rat IgG (H+L) Secondary Antibody, Alexa Fluor 555	A-21434	Thermo Fisher Scientific GmbH, Darmstadt, Germany	IF: 1:500

**Table 3:** Oligonucleotides used for real-time qRT-PCR.

Name	Primer Sequence 5′-…….-3′
Actin Fwd4	TGC CCT GAG GCT CTT TTC CA
Actin Rev4	TTG GCA TAG AGG TCT TTA CGG AT
Actin Fwd5	TGT TAC CAA CTG GGA CGA CA
Actin Rev5	ACC AGA GGC ATA CAG GGA CA
Cd44 stable Ex5 Fwd1	AGC ACC CCA GAA AGC TAC ATT
Cd44all Fwd1	TCT GCC AGG CTT TCA ACA GT
Cd44all Rev1	CTG CAC AGA TAG CGT TGG GA
Cd44v3 Rev1	TGA TCC AGA AAA ACT GGG GTA
Cd44v6 Rev1	GTT CTG AAA CCA CGT CTC CTG
Cd44v7 Rev1	TGA TCC AGA AAA ACT GGG GTA
Ccn1 Fwd1	AGA GGC TTC CTG TCT TTG GC
Ccn1 Rev1	CCA AGA CGT GGT CTG AAC GA
Ccn2 Fwd3	GTG CCA GAA CGC ACA CTG
Ccn2 Rev3	CCC CGG TTA CAC TCC AAA
Hes1 Fwd1	CCA GCC AGT GTC AAC ACG A
Hes1 Rev1	AAT GCC GGG AGC TAT CTT TCT
Hprt Fwd5	CCA TCA CAT TGT GGC CCT CT
Hprt Rev5	AAT GTA ATC CAG CAG GTC AGC A
Icam1Fwd2	TCC GTG GGG AGG AGA TAC TG
Icam1Rev2	GGC ATG AGA AAT TGG CTC CG
Itgal Fwd1	CCA CTT CCA CTT CCC GAT CT
Itgal Rev1	AGG TCT CAG GAT AGG CTG CAT
Itga4 Fwd2	TGT AGG ACA CAC CAG GCA TTC
Itga4 Rev2	TGA TGC CCA AGG TGG TAT GTG
Itgb1 Fwd1	TCG ATC CTG TGA CCC ATT GC
Itgb1 Rev1	AGT CTC CAC AAC ATG CAC GA
Itgb2 Fwd1	GCA GAA GGA CGG AAG GAA CA
Itgb2 Rev1	CCA GAT GAC CAG GAG GAG GA
Sox9 Fwd1	CAA GAC TCT GGG CAA GCT CTG
Sox9 Rev1	TCC GCT TGT CCG TTC TTC AC
Tbp Fwd1	GGC CTC TCA GAA GCA TCA CTA
Tbp Rev1	GCC AAG CCC TGA GCA TAA
Vcam1 Fwd1	AAG GGA CGA TTC CGG CAT TT
Vcam1 Rev1	TCG GGC ACA TTT CCA CAA GT

**Table 4:** Number of analyzed mice. “wk-old” stands for week-old.

Genotype	Age	Number of Mice
Control: *Alb-Cre*	2-wk-old	0
	6-wk-old	4
	20-wk-old	11
	32-wk-old	8
	48-wk-old	5
*Cd44^+/+^*;*Nf2^flox/flox^*;*Alb-Cre*	2-wk-old	5
	6-wk-old	5
	20-wk-old	12
	32-wk-old	17
	48-wk-old	9
*Cd44^−/−^*;*Nf2^flox/flox^*;*Alb-Cre*	2-wk-old	3
	6-wk-old	6
	20-wk-old	12
	32-wk-old	10
	48-wk-old	9
*Cd44^flox/flox^*;*Nf2^flox/flox^*;*Alb-Cre*	2-wk-old	0
	6-wk-old	0
	20-wk-old	0
	32-wk-old	1
	48-wk-old	5

## Data Availability

RNA-sequencing data have been deposited in the Gene Expression Omnibus (GEO) public repository under the accession GSE228630. The remaining data is contained within the article or Supplementary Materials.

## Supplementary Materials

The following supporting information can be downloaded at: https://www.mdpi.com/article/10.3390/cells12091257/s1.

## Abbreviations

AFP	α-fetoprotein
AKT	AK mouse plus transforming or thymoma
Alb	albumin
Amot	angiomotin
AraC	cytosine b-D-arabinofuranoside
ARID1A	AT-rich interactive domain-containing protein 1A
BCA	bicinchoninic acid
BIRC5	baculoviral IAP repeat containing 5
BMI1	B lymphoma Mo-MLV insertion region 1 homolog
BSA	bovine serum albumin
CD44	cluster of differentiation 44
CK19	cytokeratin 19
CPI-17	C-kinase potentiated protein phosphatase-1 Inhibitor
Cre	causes recombination
CRL4	Cullin-RING ubiquitin ligase 4
CSC	cancer stem cells
CTGF	connective tissue growth factor
CTLA-4	cytotoxic T-lymphocyte-associated protein 4
CYR61	cysteine-rich angiogenic inducer 61
DAPI	4’,6-diamidino-2-phenylindole
DCAF1	DDB1- and Cul4-associated factor 1
DMEM	Dulbecco’s Modified Eagle’s Medium
EGFR	epidermal growth factor receptor
ELISA	enzyme-linked immunosorbent assay
EMT	epithelial-mesenchymal transition
ERK	extracellular signal-regulated kinase
ERM	Ezrin-Radixin-Moesin
FAK	focal adhesion kinase
FAT1-4	FAT atypical cadherin 1-4
FBS	fetal bovine serum
FERM	4.1 protein, ezrin, radixin, moesin
FGFR	fibroblast growth factor receptor
H&E	hematoxylin and eosin
HA	hyaluronic acid
HBV	hepatitis B virus
HCC	hepatocellular carcinoma
HCV	hepatitis C virus
HEPES	2-(4-(2-hydroxyethyl)piperazin-1-yl)ethanesulfonic acid
HGF	hepatocyte growth factor
HNF4α	hepatocyte nuclear factor 4 alpha
HPRT1	hypoxanthine phosphoribosyltransferase 1
HRP	horseradish peroxidase
ICAM-1	intercellular adhesion molecule 1
iCCA	intrahepatic cholangiocarcinoma
IDH	isocitrate dehydrogenase
IL-6	interleukin-6
JAG1	Jagged 1
JAK	janus kinase
JNK	c-JUN N-terminal kinase
KIBRA	kidney and brain expressed protein
KRAS	Kirsten rat sarcoma virus
LATS1/2 LFA-1	large tumor suppressor kinase 1 and 2 lymphocyte function-associated antigen 1
MAPK	mitogen-activated protein kinase
MEK	mitogen-activated protein kinase kinase
Merlin	Moesin-Ezrin-Radixin-like protein
MET	mesenchymal epithelial transition factor receptor
MOB1A/1B	Mps one binder homolog A and B
MST1/2	mammalian STE20-like protein kinase 1 and 2
mTOR	mammalian target of rapamycin
MYPT1	myosin phosphatase targeting subunit 1
NF2	neurofibromatosis type 2
NIH	National Institutes of Health
PAK	p21-activated kinase
PBS	phosphate-buffered saline
PCR	polymerase chain reaction
PD-1	programmed cell death protein 1
PD-L1	programmed death ligand 1
PFA	paraformaldehyde
PI3K	phosphoinositide 3-kinase
PKA	protein kinase A
PP1δ	Protein Phosphatase 1 δ
PTPN3	tyrosine-protein phosphatase non-receptor type 3
RAC	Ras-related C3 botulinum toxin substrate
RAF	rapidly accelerated fibrosarcoma
RAS	rat sarcoma
RASSF	RAS association domain family
qRT-PCR	quantitative reverse transcription PCR
RT	room temperature
RTK	receptor tyrosine kinase
RUNX	Runt-related transcription factor
SAV1	Salvador homolog 1
SMAD	mothers against ecapentaplegic homolog 2
SOX9	SRY-box transcription factor 9
STAT	signal transducer and activator of transcription
TACE	transarterial chemoembolization
TAZ	transcriptional co-activator with PDZ-binding motif
TBP	TATA box binding protein
TEAD	TEA domain
TERT	telomerase reverse transcriptase
TGFβ	transforming growth factor β
TNFα	tumor necrosis factor-α
TNM	tumor, node, metastasis
TP53	tumor protein 53
TUNEL	terminal deoxynucleotidyl transferase (TdT) dUTP nick-end labeling
VCAM–1	vascular cell adhesion molecule–1
VEGF	vascular endothelial growth factor
WNT	wingless-related integration site
YAP	Yes-associated protein
