# Supplementary material for: Deletion of Cd44 Inhibits Metastasis Formation of Liver Cancer in Nf2-Mutant Mice
Source: Cells. 2023 Apr 26;12(9):1257. doi: 10.3390/cells12091257 (PMC10177437; doi:10.3390/cells12091257)
Supplement: Supplementary file 1 [file cells-12-01257-s001.zip › Figure S1.pdf]

Figure S1

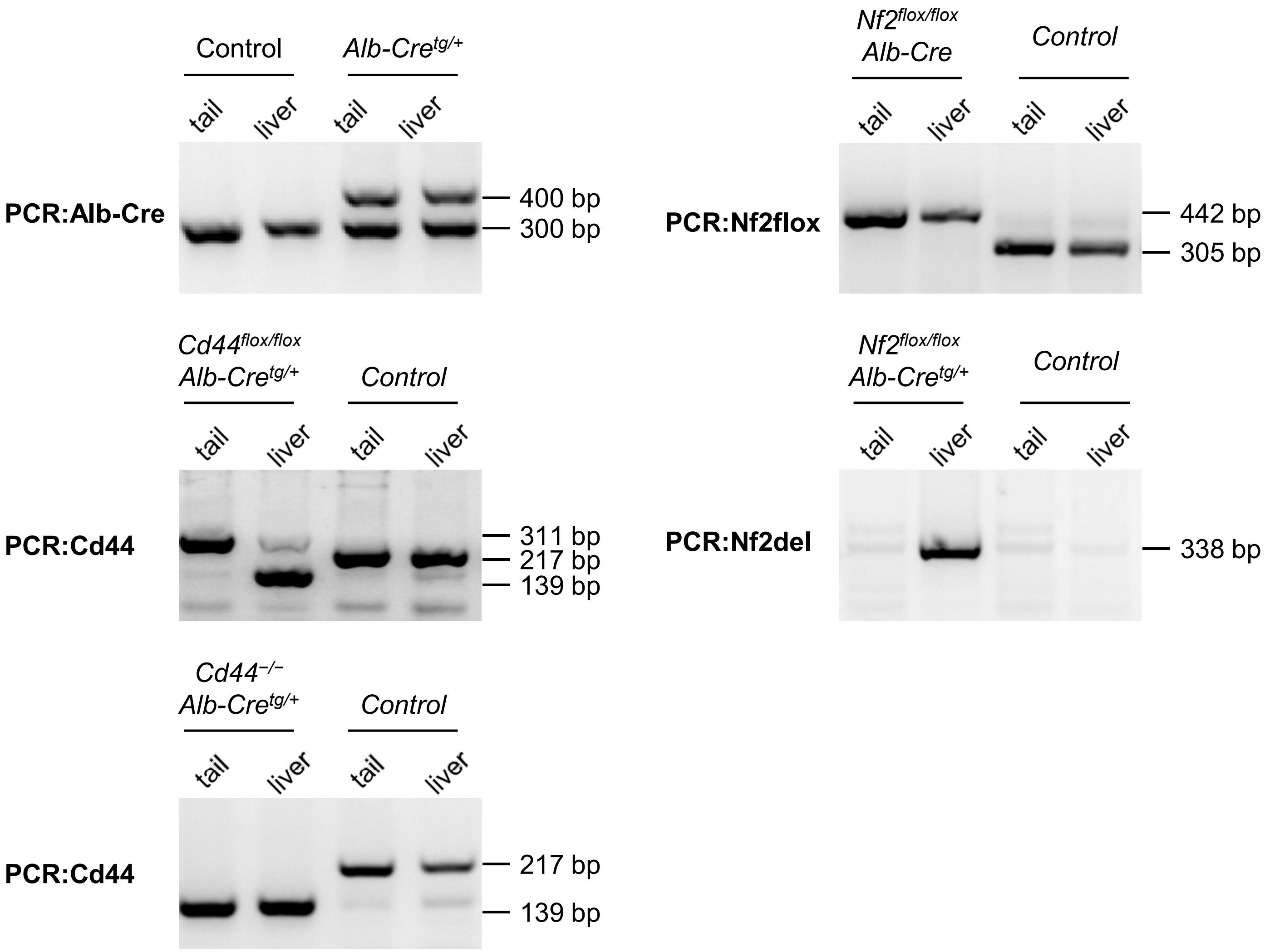
Control
Alb-Cre^tg/+
tail
liver
tail
liver
PCR:Alb-Cre
400 bp
300 bp
Nf2^flox/flox
Alb-Cre
Control
tail
liver
tail
liver
PCR:Nf2flox
442 bp
305 bp
Cd44^flox/flox
Alb-Cre^tg/+
Control
tail
liver
tail
liver
PCR:Cd44
311 bp
217 bp
139 bp
Nf2^flox/flox
Alb-Cre^tg/+
Control
tail
liver
tail
liver
PCR:Nf2del
338 bp
Cd44^−/−
Alb-Cre^tg/+
Control
tail
liver
tail
liver
PCR:Cd44
217 bp
139 bp

Figure S1. Example results of re-genotyping. Liver and tail biopsies were taken for PCR analysis after sacrifising the mice. Genotyping was performed as described in Materials and Methods. PCR products were resolved on 2-3 % agarose gels. Images were obtained using Gel-Doc EZ Imager (Bio-Rad Laboratories GmbH, Feldkirchen, Germany). The results confirmed liver-specific deletion of the *Nf2* and *Cd44* gene in *Nf2*$^{flox/flox}$;*Alb-Cre* and *Cd44*$^{flox/flox}$;*Alb-Cre* mice, respectively. *Nf2 and Cd44 flox* alleles (442 bp and 311 bp, respectively) were detected in both tail biopsies and partly in livers, whereas the deleted recombinant alleles, *Nf2 del* (338 bp) and *Cd44 del* (139 bp) were, as expected, only detected in livers of *Nf2*$^{flox/flox}$;*Alb-Cre* and *Cd44*$^{flox/flox}$;*Alb-Cre* mice, respectively. In *Alb-Cre* control livers and tails only wild type alleles of *Nf2* (305 bp) and *Cd44* (217 bp) were detected.
