# Supplementary material for: Deletion of Cd44 Inhibits Metastasis Formation of Liver Cancer in Nf2-Mutant Mice
Source: Cells. 2023 Apr 26;12(9):1257. doi: 10.3390/cells12091257 (PMC10177437; doi:10.3390/cells12091257)
Supplement: Supplementary file 1 [file cells-12-01257-s001.zip › Figure S2.pdf]

Figure S2

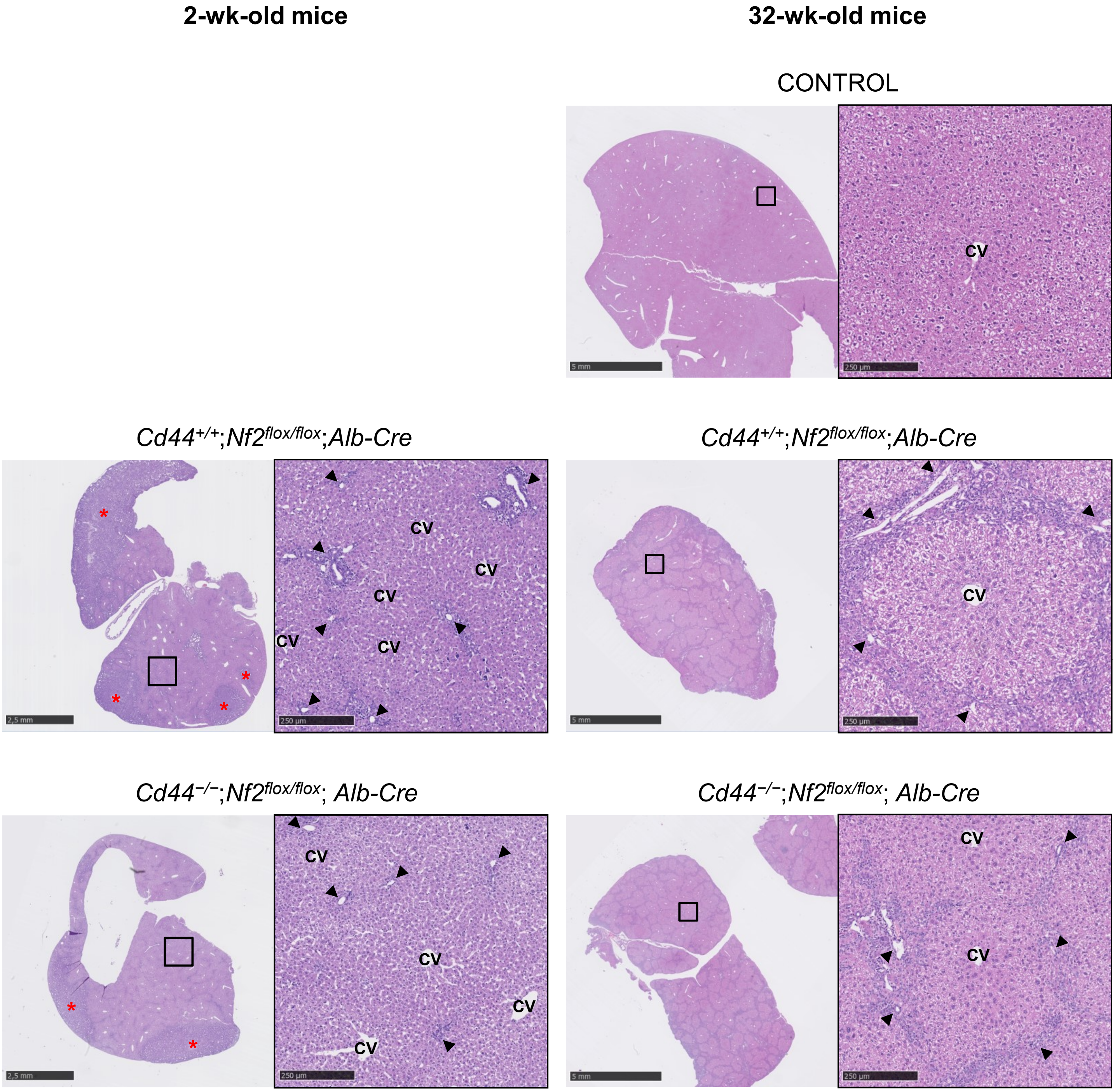
2-wk-old mice
32-wk-old mice
CONTROL
CV
5 mm
250 µm
$Cd44^{+/+}$;$Nf2^{flox/flox}$;Alb-Cre
$Cd44^{+/+}$;$Nf2^{flox/flox}$;Alb-Cre
2,5 mm
250 µm
5 mm
250 µm
$Cd44^{-/-}$;$Nf2^{flox/flox}$; Alb-Cre
$Cd44^{-/-}$;$Nf2^{flox/flox}$; Alb-Cre
2,5 mm
250 µm
5 mm
250 µm

Figure S2. H&E-stains of livers of 2- and 32-week-old (-wk-old) mice. Representative photographs were taken on NanoZoomer 2 OHT (Hamamatsu Photonics GmbH, Herrsching am Ammersee, Germany). Overview photographs depicting general liver morphology (scale bar: 2.5 and 5 mm) are shown on the left. Framed pictures on the right side represent higher magnifications (scale bar: 250 µm). Portal tracts (arrowheads) and central veins (CV) are denoted. Asterisks indicate areas with subcapsular fibrosis and ductular reaction with atrophy of liver parenchyma.
