# Supplementary material for: Deletion of Cd44 Inhibits Metastasis Formation of Liver Cancer in Nf2-Mutant Mice
Source: Cells. 2023 Apr 26;12(9):1257. doi: 10.3390/cells12091257 (PMC10177437; doi:10.3390/cells12091257)
Supplement: Supplementary file 1 [file cells-12-01257-s001.zip › Figure S3.pdf]

Figure S3

48-wk-old mice

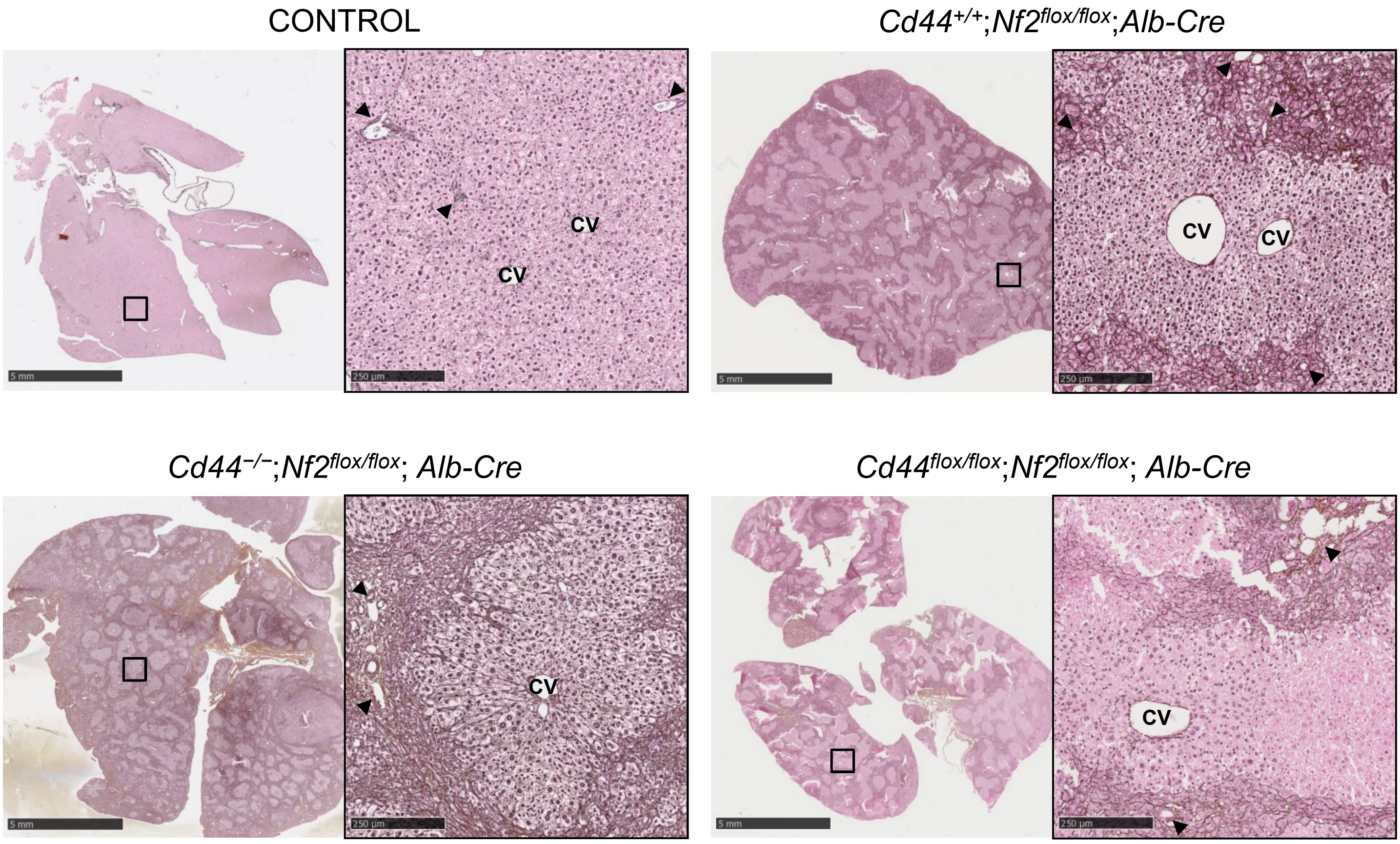

Figure S3. Gomori-silver-stain of livers of 48-week-old (-wk-old) mice. Representative photographs were taken on NanoZoomer 2 OHT (Hamamatsu Photonics GmbH, Herrsching am Ammersee, Germany). Overview photographs depicting general liver morphology (scale bar: 5 mm) are shown on the left. Framed pictures on the right side represent higher magnifications (scale bar: 250 µm). Gomori silver stains revealed massive fibrosis in the portal and periportal areas in livers of 48-wk-old *Cd44*$^{+/+}$;*Nf2*$^{flox/flox}$;*Alb-Cre*, *Cd44*$^{-/-}$;*Nf2*$^{flox/flox}$;*Alb-Cre* and *Cd44*$^{flox/flox}$;*Nf2*$^{flox/flox}$;*Alb-Cre* mice (visible as brown/black areas). Portal tracts (arrowheads) and central veins (CV) are denoted.
