# Supplementary material for: Deletion of Cd44 Inhibits Metastasis Formation of Liver Cancer in Nf2-Mutant Mice
Source: Cells. 2023 Apr 26;12(9):1257. doi: 10.3390/cells12091257 (PMC10177437; doi:10.3390/cells12091257)
Supplement: Supplementary file 1 [file cells-12-01257-s001.zip › Figure S4.pdf]

Figure S4

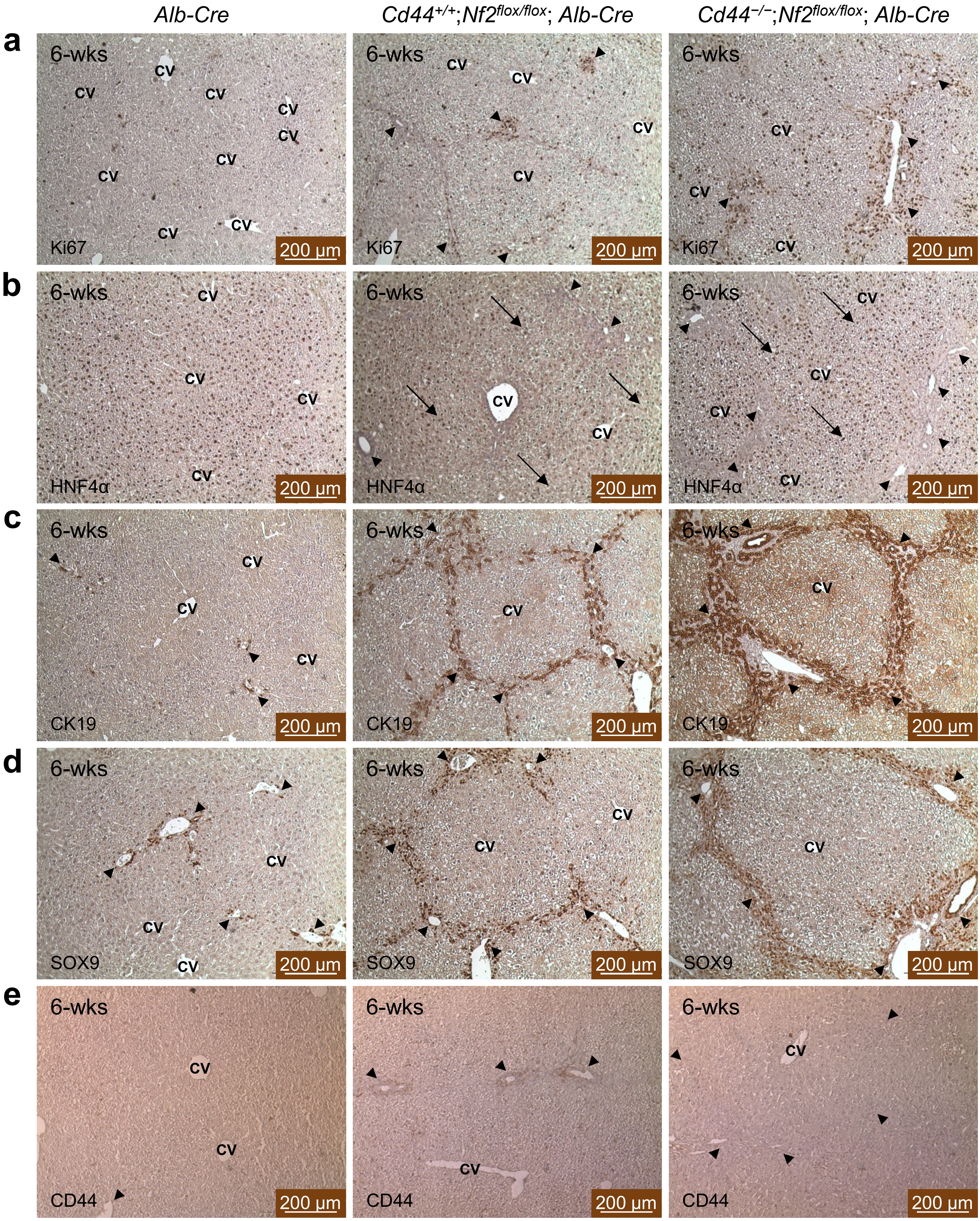
Alb-Cre
$Cd44^{+/+}$;$Nf2^{flox/flox}$; Alb-Cre
$Cd44^{-/-}$;$Nf2^{flox/flox}$; Alb-Cre
a
b
c
d
e
6-wks
cv
Ki67
HNF4α
CK19
SOX9
CD44
200 µm

Figure S4. Immunohistochemical staining of 6-week-old livers from *Nf2*-mutant mice. (a) Ki67 staining revealed presence of ductular reactions in portal tracts of *Cd44*$^{+/+}$;*Nf2*$^{flox/flox}$;*Alb-Cre*, *Cd44*$^{-/}$;*Nf2*$^{flox/flox}$;*Alb-Cre* livers. Ki67 staining was nearly undetectable in control livers *(Alb-Cre*). **(b)** Immunohistochemical detection of HNF4α as marker of hepatocytes. **(c** and **d)** Immunohistochemical analysis using an anti-CK19 and anti-SOX9 antibody revealed prominent and progressive staining in the tumor-like ductular proliferations of livers isolated from *Cd44*$^{+/+}$;*Nf2*$^{flox/flox}$;*Alb-Cre* and *Cd44*$^{-/-}$;*Nf2*$^{flox/flox}$;*Alb-Cre* mice. In contrast, in the *Alb-Cre* control livers, only a subset of portal bile duct cells expressed CK19 and SOX9 antigens. **(e)** Immunohistochemical analysis demonstrated prominent CD44 staining in bile ducts and ductular proliferations of livers isolated from *Cd44*$^{+/+}$;*Nf2*$^{flox/flox}$;*Alb-Cre* mice. CD44 staining was undetectable in *Cd44*$^{-/-}$;*Nf2*$^{flox/flox}$;*Alb-Cre* mice. Central veins (CV), portal tracts (arrowheads) and hepatocytes (arrows) are denoted. Photographs were taken using a Leica DFC290 microscope. Scale bar: 200 μm.
