# Supplementary material for: Deletion of Cd44 Inhibits Metastasis Formation of Liver Cancer in Nf2-Mutant Mice
Source: Cells. 2023 Apr 26;12(9):1257. doi: 10.3390/cells12091257 (PMC10177437; doi:10.3390/cells12091257)
Supplement: Supplementary file 1 [file cells-12-01257-s001.zip › Figure S5.pdf]

Figure S5

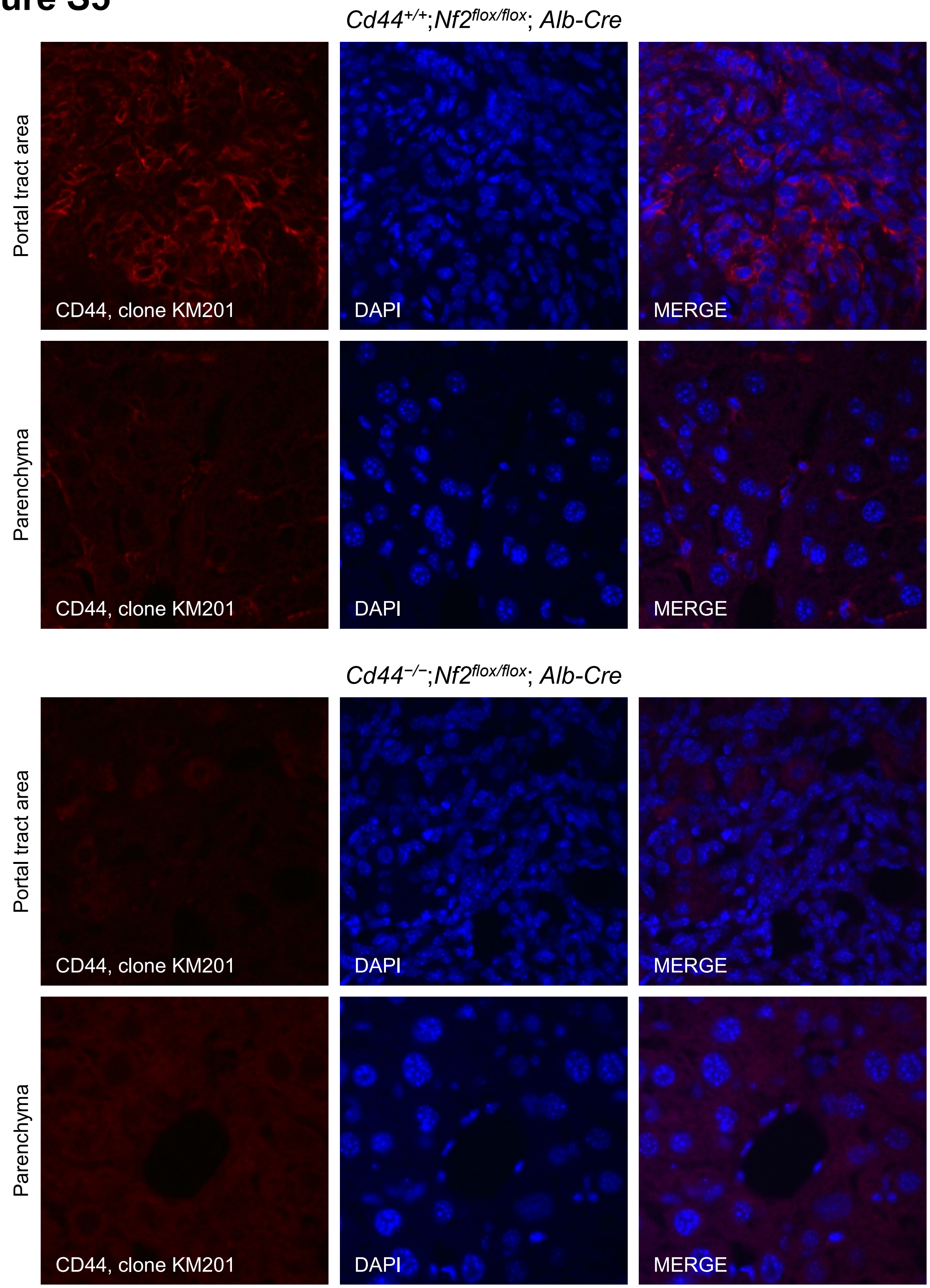

Cd44$^{+/+}$;*Nf2*$^{flox/flox}$; *Alb-Cre*
Portal tract area
Parenchyma
CD44, clone KM201
DAPI
MERGE
CD44, clone KM201
DAPI
MERGE
Cd44$^{-/-}$;*Nf2*$^{flox/flox}$; *Alb-Cre*
Portal tract area
Parenchyma
CD44, clone KM201
DAPI
MERGE
CD44, clone KM201
DAPI
MERGE

Figure S5. Immunofluorescent localization of CD44 in livers isolated from 32-week-old *Cd44*$^{+/+}$;*Nf2*$^{flox/flox}$;*Alb-Cre* and *Cd44*$^{-/-}$;*Nf2*$^{flox/flox}$;*Alb-Cre* mice. CD44 was detected using CD44 primary antibody, clone KM201. A secondary antibody conjugated to Alexa Fluor 555 was used for immunofluorescent detection. Cell nuclei were stained with DAPI. Fluorescent photographs were generated with an ApoTome Axiovert 200 microscope (Carl Zeiss Meditec AG, Jena, Germany) with 40x magnification.
