# Supplementary material for: Deletion of Cd44 Inhibits Metastasis Formation of Liver Cancer in Nf2-Mutant Mice
Source: Cells. 2023 Apr 26;12(9):1257. doi: 10.3390/cells12091257 (PMC10177437; doi:10.3390/cells12091257)
Supplement: Supplementary file 1 [file cells-12-01257-s001.zip › Figure S6.pdf]

Figure S6

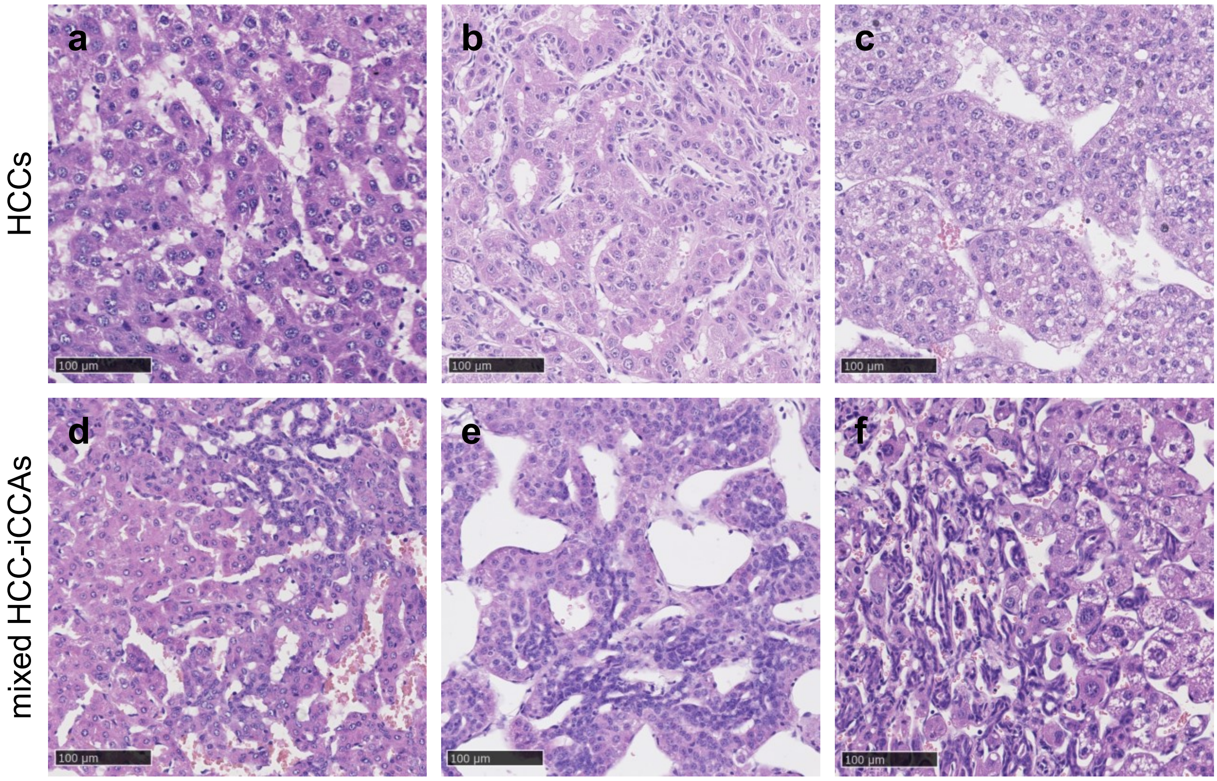

a
b
c
d
e
f
HCCs
mixed HCC-iCCAs
100 μm
100 μm
100 μm
100 μm
100 μm
100 μm

Figure S6. Spectrum of tumors detected in *Nf2$^{flox/flox}$;Alb-Cre* mice. (a) HCC detected in *Cd44$^{+/+}$;Nf2$^{flox/flox}$;Alb-Cre* mice; **(b)** pseudoglandular HCC detected in a *Cd44$^{-/-}$;Nf2$^{flox/flox}$;Alb-Cre* mouse; **(c)** macrotrabecular HCC detected in a *Cd44$^{-/-}$;Nf2$^{flox/flox}$;Alb-Cre* mouse; tumors of mixed HCC-iCCA morphology detected in *Cd44$^{+/+}$;Nf2$^{flox/flox}$;Alb-Cre* **(d)**, *Cd44$^{-/-}$;Nf2$^{flox/flox}$;Alb-Cre* **(e)** and *Cd44$^{flox/flox}$;Nf2$^{flox/flox}$;Alb-Cre* **(f)** mice. Scale bar: 100 μm.
