# Supplementary material for: Deletion of Cd44 Inhibits Metastasis Formation of Liver Cancer in Nf2-Mutant Mice
Source: Cells. 2023 Apr 26;12(9):1257. doi: 10.3390/cells12091257 (PMC10177437; doi:10.3390/cells12091257)
Supplement: Supplementary file 1 [file cells-12-01257-s001.zip › Figure S7.pdf]

Figure S7

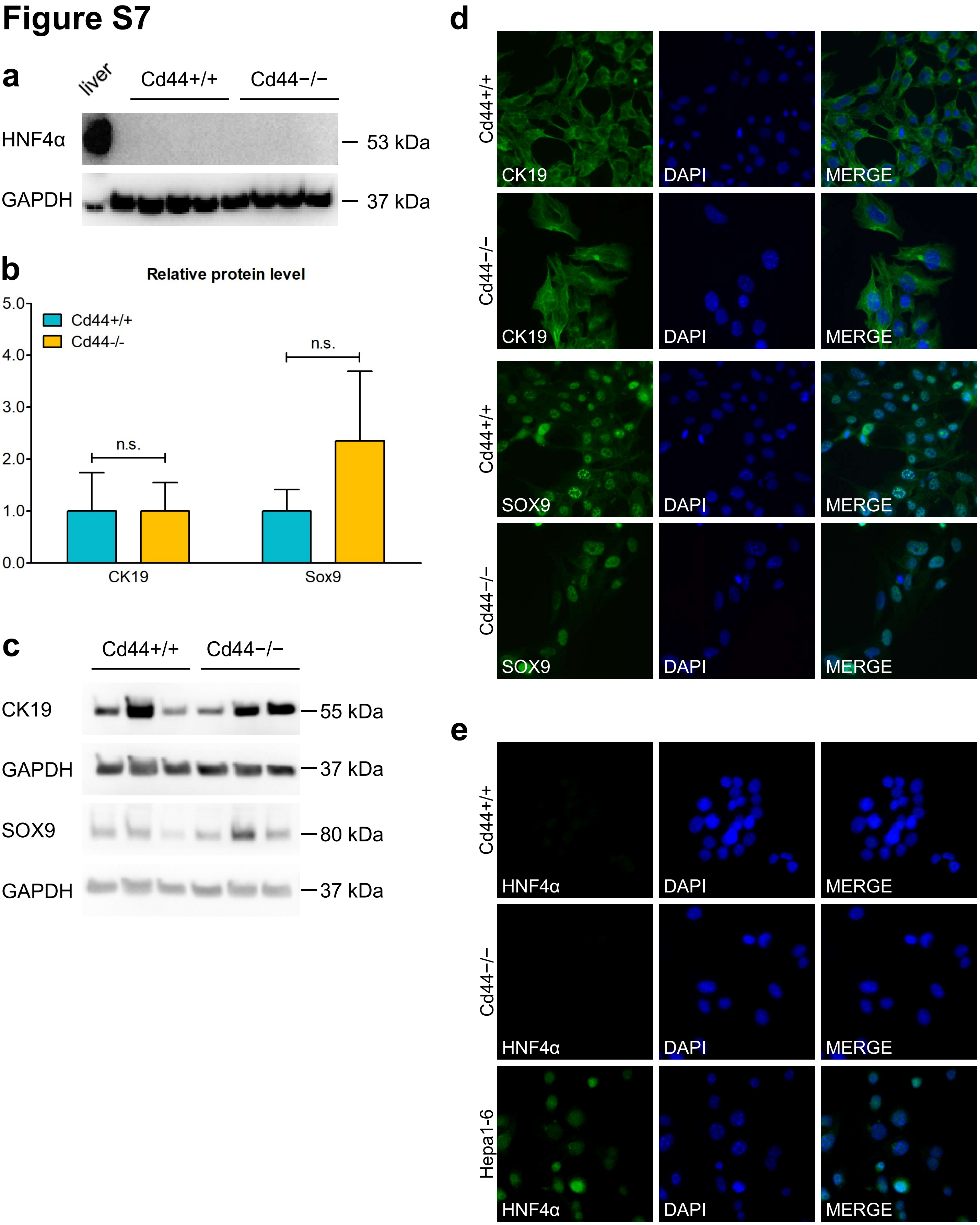

a
liver
Cd44+/+
Cd44−/−
HNF4α
53 kDa
GAPDH
37 kDa
b
Relative protein level
5.0
4.0
3.0
2.0
1.0
0.0
Cd44+/+
Cd44-/-
n.s.
n.s.
CK19
Sox9
c
Cd44+/+
Cd44−/−
CK19
55 kDa
GAPDH
37 kDa
SOX9
80 kDa
GAPDH
37 kDa
d
Cd44+/+
Cd44−/−
CK19
DAPI
MERGE
SOX9
e
HNF4α
Hepa1-6

Figure S7. Detection of liver markers in cell lines. (a - c) Testing HNF4α, CK19 and SOX9 expression in liver cell lines by immunoblot. *Cd44*-positive and *Cd44*-negative liver cell lines were generated from $Cd44^{+/+}$;$Nf2^{flox/flox}$;*Alb-Cre* and $Cd44^{-/-}$;$Nf2^{flox/flox}$;*Alb-Cre* mice. Three to four independent $Cd44^{+/+}$ and $Cd44^{-/-}$ cell lines were tested. Liver tissue lysates were applied as a positive control for detection of HNF4α. The cells were seeded at 50% confluency in DMEM medium supplemented with 10% FBS. The cells were left for overnight to attach and then subjected to immunoblot. HNF4α was detected using anti-HNF4α antibody, clone K9218. CK19 was detected using anti-CK19 antibody, clone B-1, SOX9 was detected using polyclonal antibody. GAPDH was detected to control equal loading of samples. **(b)** The bar chart shows mean CK19 and SOX9 protein levels normalized to GAPDH ±SD from three independent $Cd44^{+/+}$ and $Cd44^{-/-}$ cell lines. **(d, e)** Immunofluorescent localization of CK19, SOX9 and HNF4α in $Cd44^{+/+}$ and $Cd44^{-/-}$ cell lines isolated from $Nf2^{flox/flox}$;*Alb-Cre* mice. Hepa1-6 cell line served as a positive control for detection of HNF4α. *Cd44*-positive and *Cd44*-negative liver cell lines were seeded in 12-well plates at a density of $2x10^5$ cells per well. Indicated liver cell markers were detected according to immunocytochemistry protocol. Secondary antibodies conjugated to Alexa Fluor 488 were used for immunofluorescent detection. Cell nuclei were stained with DAPI. Fluorescent photographs were generated with an ApoTome Axiovert 200 microscope with 40x magnification. Immunostaining confirmed cytoplasmic localization of CK19, which builds intermediate filaments and nuclear localization of transcription factors SOX9 and HNF4α in cultured cells.
