# Supplementary material for: Deletion of Cd44 Inhibits Metastasis Formation of Liver Cancer in Nf2-Mutant Mice
Source: Cells. 2023 Apr 26;12(9):1257. doi: 10.3390/cells12091257 (PMC10177437; doi:10.3390/cells12091257)
Supplement: Supplementary file 1 [file cells-12-01257-s001.zip › Figure S8.pdf]

Figure S8

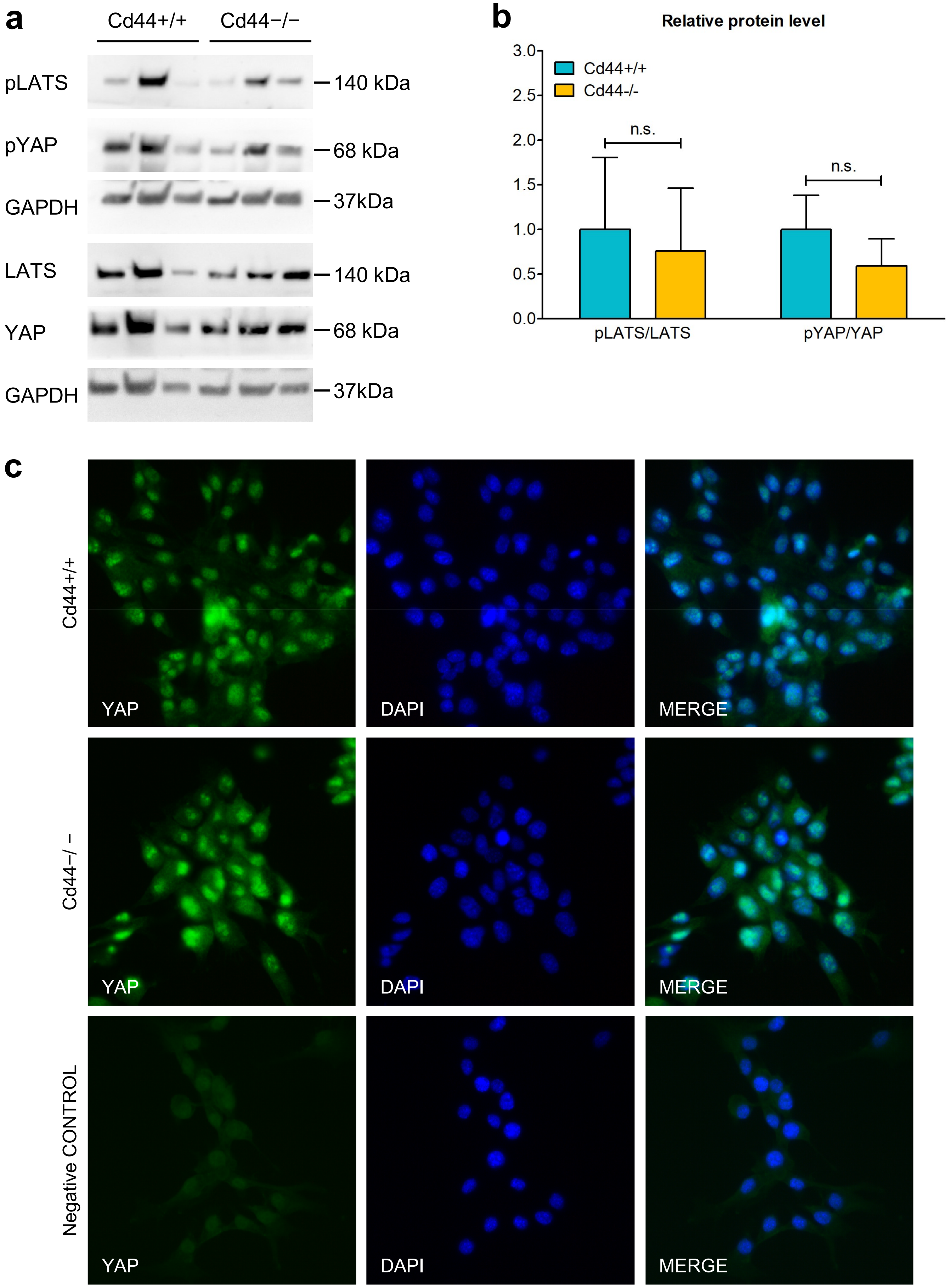
a
Cd44+/+
Cd44−/−
pLATS
140 kDa
pYAP
68 kDa
GAPDH
37kDa
LATS
140 kDa
YAP
68 kDa
GAPDH
37kDa
b
Relative protein level
Cd44+/+
Cd44-/-
n.s.
n.s.
3.0
2.5
2.0
1.5
1.0
0.5
0.0
pLATS/LATS
pYAP/YAP
c
Cd44+/+
Cd44−/ −
Negative CONTROL
YAP
DAPI
MERGE

Figure S8. Influence of CD44 on YAP and LATS expression and activation. (a, b) Detection of total and phosphorylated YAP and LATS in liver cell lines by immunoblot. *Cd44*-positive and *Cd44*-negative liver cell lines were generated from *Cd44*$^{+/+}$;*Nf2*$^{flox/flox}$;*Alb-Cre* and *Cd44*$^{-/-}$;*Nf2*$^{flox/flox}$;*Alb-Cre* mice. The cells were seeded at 50% confluency in DMEM medium supplemented with 10% FBS. The cells were left for overnight to attach and then subjected to immunoblot analysis. LATS1 and phospho-LATS (Thr1079) were detected using anti-LATS antibody, clone C66B5 and anti phospho-LATS1 antibody, clone D57D3. YAP and phospho-YAP (Ser127) were detected using polyclonal antibodies. GAPDH was detected to control equal loading of samples. The bar chart shows mean pLATS/LATS and pYAP/YAP protein levels normalized to GAPDH ±SD from three independent *Cd44*$^{+/+}$ and *Cd44*$^{-/-}$ cell lines. **(c)** Immunofluorescent localization of YAP in liver cells isolated from *Cd44*-positive and *Cd44*-negative *Nf2*$^{flox/flox}$;*Alb-Cre* mice. YAP was detected according to immunocytochemistry protocol. Secondary antibody conjugated to Alexa Fluor 488 was used for immunofluorescent detection. Cell nuclei were stained with DAPI. Fluorescent photographs were generated with an ApoTome Axiovert 200 microscope (Carl Zeiss Meditec AG, Jena, Germany) with 40x magnification.
