# Supplementary material for: Deletion of Cd44 Inhibits Metastasis Formation of Liver Cancer in Nf2-Mutant Mice
Source: Cells. 2023 Apr 26;12(9):1257. doi: 10.3390/cells12091257 (PMC10177437; doi:10.3390/cells12091257)
Supplement: Supplementary file 1 [file cells-12-01257-s001.zip › Table S1.pdf]

Table S1. List of genes that are differentially regulated between *Cd44*-positive and *Cd44*-negative livers. The genes were determined using rlog function of DESeq2 package. All genes with Benjamin Hochberg adjusted p-value **(padj)** <0.05 were included. The data are represented as Wald-statistic (**stat**). Genes with negative Wald-statistic values are down-regulated in $Cd44^{-/-}$ compared to $Cd44^{+/+}$ livers and genes with positive values are upregulated. In total 918 differentially regulated genes were identified.

$$\text{Wald-statistic} = \frac{\text{log2FoldChange}}{\text{standard error of}}$$

Ensembl_id	Symbol	*Cd44*[+/+]_*Cd44*[–/–]_padj	*Cd44*[+/+]_*Cd44*[–/–]_stat
ENSMUSG00000082097	Gm14039	0.0000000355599	-5.51159
ENSMUSG00000060216	Arrb2	0.000000241744	-5.16399
ENSMUSG00000022489	Pde1b	0.00000057402	-4.99976
ENSMUSG00000005087	Cd44	0.00000079405	-4.93682
ENSMUSG00000040809	Chi3l3	0.00000427489	-4.59755
ENSMUSG00000096699	Rps19-ps4	0.00000503825	-4.56319
ENSMUSG00000056399	Prss34	0.0000161678	-4.31215
ENSMUSG00000043740	B430306N03Rik	0.000038231	-4.11792
ENSMUSG00000056054	S100a8	0.0000782245	-3.94978
ENSMUSG00000028644	Ermap	0.000101048	-3.88806
ENSMUSG00000026042	Col5a2	0.000101537	-3.88689
ENSMUSG00000043336	Filip1l	0.000125532	-3.83506
ENSMUSG00000039585	Myo9a	0.000131958	-3.82278
ENSMUSG00000008845	Cd163	0.000144649	-3.80008
ENSMUSG00000022667	Cd200r1	0.000208243	-3.7088
ENSMUSG00000044071	Fam19a2	0.000209682	-3.70706
ENSMUSG00000067575	Rpl35a-ps3	0.00021714	-3.69819
ENSMUSG00000029299	Abcg3	0.000225834	-3.68821
ENSMUSG00000038357	Camp	0.000227662	-3.68616
ENSMUSG00000029925	Tbxas1	0.000235681	-3.67734
ENSMUSG00000056071	S100a9	0.000256543	-3.65564
ENSMUSG00000082633	Gm15779	0.00028583	-3.62782
ENSMUSG00000045551	Fpr1	0.0003013	-3.61418
ENSMUSG00000036411	9530077C05Rik	0.000316878	-3.6011
ENSMUSG00000025877	Hk3	0.000374738	-3.55726
ENSMUSG00000066270	Gm10157	0.000404883	-3.53688
ENSMUSG00000060044	Tmem26	0.000424532	-3.52434
ENSMUSG00000034116	Vav1	0.000459182	-3.5035
ENSMUSG00000003283	Hck	0.000465228	-3.50002
ENSMUSG00000042228	Lyn	0.000468083	-3.49839
ENSMUSG00000050994	Adgb	0.000498822	-3.48139
ENSMUSG00000040957	Cables1	0.000509104	-3.47592
ENSMUSG00000006235	Epor	0.000601914	-3.43075
ENSMUSG00000034041	Lyl1	0.000738351	-3.37493
ENSMUSG00000070323	Mmp27	0.00074794	-3.37137
ENSMUSG00000029866	Kel	0.000864778	-3.33118
ENSMUSG00000084149	Gm12944	0.000894737	-3.32169
ENSMUSG00000029819	Npy	0.000899539	-3.3202
ENSMUSG00000030474	Siglece	0.000920141	-3.31387
ENSMUSG00000025255	Zfhx4	0.0009763	-3.29727
ENSMUSG00000029919	Hpgds	0.000987732	-3.294
ENSMUSG00000059089	Fcgr4	0.00103125	-3.28186
ENSMUSG00000061758	Akr1b10	0.00106178	-3.27362
ENSMUSG00000059108	Ifitm6	0.00108616	-3.2672
ENSMUSG00000031129	Slc9a9	0.00108999	-3.26621
ENSMUSG00000004359	Spic	0.00116257	-3.24791
ENSMUSG00000072844	G530011O06Rik	0.00117118	-3.24581
ENSMUSG00000024789	Jak2	0.00118752	-3.24186
ENSMUSG00000033192	Lpcat2	0.00119816	-3.23932
ENSMUSG00000038725	Pkhd1l1	0.00122068	-3.234
ENSMUSG00000094156	Sult2a7	0.00125339	-3.22644
ENSMUSG00000009292	Trpm2	0.00134902	-3.20534

ENSMUSG00000006574	Slc4a1	0.0013828	-3.19822
ENSMUSG00000026480	Ncf2	0.00141927	-3.1907
ENSMUSG00000032134	Muc16	0.00145791	-3.18293
ENSMUSG00000037697	Ddhd1	0.00147905	-3.17876
ENSMUSG00000038540	Tmc3	0.00149649	-3.17536
ENSMUSG00000057191	AB124611	0.00150496	-3.17373
ENSMUSG00000059326	Csf2ra	0.00158233	-3.15915
ENSMUSG00000085442	Gm3362	0.00159536	-3.15675
ENSMUSG00000042129	Rassf4	0.00164727	-3.1474
ENSMUSG00000028825	Rhd	0.00166166	-3.14486
ENSMUSG00000023995	Tspo2	0.00171362	-3.13584
ENSMUSG00000043832	Clec4a3	0.00171756	-3.13517
ENSMUSG00000059900	Tmem40	0.00187627	-3.10915
ENSMUSG00000042066	Tmcc2	0.001879	-3.10872
ENSMUSG00000078154	Gm12184	0.00198764	-3.09207
ENSMUSG00000016984	Etaa1	0.00210978	-3.07433
ENSMUSG00000026928	Card9	0.00213379	-3.07095
ENSMUSG00000069814	E130309D14Rik	0.00222965	-3.0578
ENSMUSG00000027082	Tfpi	0.002281	-3.05097
ENSMUSG00000039115	Itga9	0.00229012	-3.04978
ENSMUSG00000030041	M1ap	0.00239022	-3.0369
ENSMUSG00000057897	Camk2b	0.00240071	-3.03558
ENSMUSG00000032702	Kank1	0.00242588	-3.03244
ENSMUSG00000052248	Gm13476	0.00255094	-3.01723
ENSMUSG00000054752	Fsd1l	0.00259209	-3.01238
ENSMUSG00000066682	Pilrb2	0.00259593	-3.01193
ENSMUSG00000087107	AI662270	0.00260037	-3.01141
ENSMUSG00000004552	Ctse	0.00263372	-3.00754
ENSMUSG00000022957	Itsn1	0.00270822	-2.99905
ENSMUSG00000014361	Mertk	0.00276457	-2.99277
ENSMUSG00000024621	Csf1r	0.00280235	-2.98863
ENSMUSG00000024663	Rab3il1	0.00280941	-2.98786
ENSMUSG00000089672	Gp49a	0.00289021	-2.97918
ENSMUSG00000026708	Cenpl	0.00302338	-2.96535
ENSMUSG00000024235	Map3k8	0.00305471	-2.96218
ENSMUSG00000038523	1700003F12Rik	0.00307839	-2.9598
ENSMUSG00000030147	Clec4b1	0.0031074	-2.95691
ENSMUSG00000029484	Anxa3	0.0031333	-2.95435
ENSMUSG00000037872	Darc	0.0031927	-2.94855
ENSMUSG00000018909	Arrb1	0.00320417	-2.94744
ENSMUSG00000044313	Mab21l3	0.00322959	-2.945
ENSMUSG00000085394	2210414B05Rik	0.00324011	-2.94399
ENSMUSG00000019088	Dnase1l1	0.00329302	-2.93897
ENSMUSG00000005237	Dnah2	0.00333875	-2.9347
ENSMUSG00000052212	Cd177	0.00340071	-2.92898
ENSMUSG00000058818	Pirb	0.00341275	-2.92789
ENSMUSG00000073412	Lst1	0.00342612	-2.92667
ENSMUSG00000058715	Fcer1g	0.00399824	-2.8783
ENSMUSG00000038058	Nod1	0.00400255	-2.87796
ENSMUSG00000056656	Apol8	0.00408156	-2.87179
ENSMUSG00000020205	Phlda1	0.0040925	-2.87094
ENSMUSG00000032915	Emr4	0.00414821	-2.86667
ENSMUSG00000089929	Bcl2a1b	0.00424031	-2.85971

ENSMUSG00000021597	Ankrd32	0.00429432	-2.85569
ENSMUSG00000046245	Pilra	0.00430348	-2.85501
ENSMUSG00000049225	Pdp1	0.00442821	-2.84593
ENSMUSG00000039294	BC017643	0.0044304	-2.84577
ENSMUSG00000024440	Pcdh12	0.00448839	-2.84163
ENSMUSG00000087594	BC039771	0.00451577	-2.83969
ENSMUSG00000026177	Slc11a1	0.00452694	-2.8389
ENSMUSG00000030247	Kcnj8	0.00464031	-2.831
ENSMUSG00000015355	Cd48	0.00466598	-2.82923
ENSMUSG00000027314	Dll4	0.0047798	-2.82151
ENSMUSG00000030047	Arhgap25	0.00485199	-2.8167
ENSMUSG00000027200	Sema6d	0.00486637	-2.81575
ENSMUSG00000068036	Mllt4	0.00494493	-2.8106
ENSMUSG00000078780	Gm5150	0.00503187	-2.80499
ENSMUSG00000021675	F2rl2	0.0050364	-2.8047
ENSMUSG00000030187	Klra2	0.00509559	-2.80093
ENSMUSG00000064147	Rab44	0.00513545	-2.79841
ENSMUSG00000034947	Tmem106a	0.00517702	-2.79581
ENSMUSG00000031441	Atp11a	0.00518975	-2.79501
ENSMUSG00000023216	Epb4.2	0.00521683	-2.79333
ENSMUSG00000027615	Hps3	0.00531228	-2.78746
ENSMUSG00000060149	BC002059	0.00535728	-2.78473
ENSMUSG00000074006	Omp	0.00536406	-2.78432
ENSMUSG00000046417	Fam211a	0.0055661	-2.7723
ENSMUSG00000023078	Cxcl13	0.00560029	-2.77031
ENSMUSG00000030787	Lyve1	0.00573551	-2.76253
ENSMUSG00000038751	Ptk6	0.00576054	-2.76111
ENSMUSG00000031712	Il15	0.00580972	-2.75833
ENSMUSG00000083748	Gm11662	0.00592051	-2.75215
ENSMUSG00000045917	6330416G13Rik	0.00594278	-2.75092
ENSMUSG00000020057	Dram1	0.00595444	-2.75028
ENSMUSG00000010342	Tex14	0.00598222	-2.74875
ENSMUSG00000035004	Igsf6	0.00598884	-2.74839
ENSMUSG00000004730	Emr1	0.00603962	-2.74562
ENSMUSG00000049037	Clec4a1	0.00604285	-2.74545
ENSMUSG00000025185	Loxl4	0.00608272	-2.74329
ENSMUSG00000034037	Fgd5	0.00621678	-2.73612
ENSMUSG00000085761	4930455G09Rik	0.00625992	-2.73385
ENSMUSG00000020589	Fam49a	0.00628674	-2.73244
ENSMUSG00000024533	Spire1	0.00629476	-2.73202
ENSMUSG00000062157	Ifnlr1	0.00642058	-2.72549
ENSMUSG00000024397	Aif1	0.00643914	-2.72454
ENSMUSG00000044811	AF251705	0.00644869	-2.72405
ENSMUSG00000021322	Aoah	0.00647268	-2.72282
ENSMUSG00000061451	Tmem151a	0.00662705	-2.71503
ENSMUSG00000020290	Xpo1	0.00681103	-2.70595
ENSMUSG00000027210	Meis2	0.00693211	-2.70009
ENSMUSG00000070369	Itgad	0.00695067	-2.6992
ENSMUSG00000052917	Senp7	0.00697159	-2.6982
ENSMUSG00000019813	Cep57l1	0.00698869	-2.69738
ENSMUSG00000000489	Pdgfb	0.0069934	-2.69716
ENSMUSG00000087450	Gm13994	0.007213	-2.68685
ENSMUSG00000024013	Fgd2	0.00724684	-2.68528

ENSMUSG00000022099	Dmtn	0.00730685	-2.68253
ENSMUSG00000052160	Pld4	0.00738468	-2.67898
ENSMUSG00000010307	Tmem86a	0.00743645	-2.67664
ENSMUSG00000038843	Gcnt1	0.00747183	-2.67505
ENSMUSG00000096603	Gm7694	0.00747639	-2.67484
ENSMUSG00000027829	Ccnl1	0.00750671	-2.67349
ENSMUSG00000030148	Clec4a2	0.0075818	-2.67015
ENSMUSG00000020142	Slc1a4	0.00761605	-2.66863
ENSMUSG00000037031	Tspan15	0.00787166	-2.65753
ENSMUSG00000039693	Msantd3	0.00787632	-2.65733
ENSMUSG00000051839	Gypa	0.0079069	-2.65602
ENSMUSG00000026028	Trak2	0.00792939	-2.65506
ENSMUSG00000025058	5430427O19Rik	0.00798039	-2.6529
ENSMUSG00000085099	Gm16288	0.00798429	-2.65273
ENSMUSG00000079442	St6galnac4	0.00809687	-2.648
ENSMUSG00000042408	Zmym6	0.00813486	-2.64642
ENSMUSG00000030793	Pycard	0.00814351	-2.64606
ENSMUSG00000062593	Lilrb4	0.00820963	-2.64332
ENSMUSG00000029007	Agtrap	0.00848334	-2.6322
ENSMUSG00000026737	Pip4k2a	0.00849485	-2.63174
ENSMUSG00000026390	Marco	0.00858509	-2.62815
ENSMUSG00000048498	Cd300e	0.00860776	-2.62725
ENSMUSG00000058794	Nfe2	0.00864488	-2.62579
ENSMUSG00000019878	Hsf2	0.00897405	-2.61304
ENSMUSG00000027562	Car2	0.00932064	-2.60006
ENSMUSG00000033446	Lpar6	0.0094003	-2.59714
ENSMUSG00000040473	A330021E22Rik	0.00948574	-2.59403
ENSMUSG00000022969	Il10rb	0.00955471	-2.59154
ENSMUSG00000027620	Rbm39	0.00962635	-2.58897
ENSMUSG00000066361	Serpina3c	0.0097485	-2.58463
ENSMUSG00000049988	Lrrc25	0.0101308	-2.57133
ENSMUSG00000086436	Gm13690	0.0101902	-2.56931
ENSMUSG00000037902	Sirpa	0.0102639	-2.56681
ENSMUSG00000030557	Mef2a	0.0103138	-2.56513
ENSMUSG00000025154	Arhgap19	0.0103599	-2.56358
ENSMUSG00000040528	Milr1	0.0103837	-2.56278
ENSMUSG00000034641	Cd300ld	0.010597	-2.55571
ENSMUSG00000060962	Dmkn	0.0105997	-2.55563
ENSMUSG00000005131	4930550C14Rik	0.010761	-2.55036
ENSMUSG00000081254	Gm12112	0.0109238	-2.54513
ENSMUSG00000037860	Aim2	0.0109423	-2.54454
ENSMUSG00000071722	Spin4	0.0110308	-2.54172
ENSMUSG00000019139	Isyna1	0.0111339	-2.53847
ENSMUSG00000026483	Fam129a	0.0111792	-2.53705
ENSMUSG00000045404	Kcnk13	0.01134	-2.53204
ENSMUSG00000039531	Zufsp	0.0114382	-2.52902
ENSMUSG00000048621	Gm6377	0.011802	-2.51801
ENSMUSG00000084828	Gm12367	0.0118683	-2.51604
ENSMUSG00000037251	Pomk	0.0119102	-2.51479
ENSMUSG00000005800	Mmp8	0.0119746	-2.51289
ENSMUSG00000047735	Samd9l	0.0121502	-2.50775
ENSMUSG00000022102	Dok2	0.0122323	-2.50537
ENSMUSG00000060336	Zfp937	0.0123187	-2.50288

ENSMUSG00000037306	Man1c1	0.0124308	-2.49967
ENSMUSG00000025287	Acot9	0.0124746	-2.49843
ENSMUSG00000018983	E2f2	0.0124991	-2.49773
ENSMUSG00000020599	Rgs9	0.0125803	-2.49543
ENSMUSG00000090863	A530084C06Rik	0.0126667	-2.49301
ENSMUSG00000030446	Zfp273	0.0128313	-2.48842
ENSMUSG00000034059	Ypel4	0.0128878	-2.48686
ENSMUSG00000026630	Batf3	0.0130648	-2.482
ENSMUSG00000055541	Lair1	0.0131115	-2.48073
ENSMUSG00000040699	Limd2	0.0131898	-2.4786
ENSMUSG00000098495	RP24-113D21.1	0.0132794	-2.47619
ENSMUSG00000031004	Mki67	0.0134482	-2.47167
ENSMUSG00000026360	Rgs2	0.0134947	-2.47044
ENSMUSG00000040093	Bmf	0.0137153	-2.46464
ENSMUSG00000020263	Appl2	0.0139223	-2.45926
ENSMUSG00000030708	Dnajb13	0.0140233	-2.45667
ENSMUSG00000031963	Bmper	0.0141738	-2.45283
ENSMUSG00000029762	Akr1b8	0.0142802	-2.45014
ENSMUSG00000029380	Cxcl1	0.0144084	-2.44692
ENSMUSG00000019876	Pkib	0.0144542	-2.44577
ENSMUSG00000030263	Lrmp	0.0144778	-2.44518
ENSMUSG00000054737	Zfp182	0.0147277	-2.43901
ENSMUSG00000030409	Dmpk	0.0147279	-2.439
ENSMUSG00000020787	P2rx1	0.0148024	-2.43718
ENSMUSG00000055069	Rab39	0.0148099	-2.437
ENSMUSG00000026315	Serpinb8	0.0149312	-2.43404
ENSMUSG00000097440	Gm6277	0.0150024	-2.43232
ENSMUSG00000060380	C030014I23Rik	0.0152361	-2.42672
ENSMUSG00000047250	Ptgs1	0.01524	-2.42662
ENSMUSG00000001767	Crnkl1	0.0152416	-2.42659
ENSMUSG00000030402	Ppm1n	0.0152681	-2.42596
ENSMUSG00000036718	Micall2	0.0154821	-2.4209
ENSMUSG00000019590	Cyb561	0.0155454	-2.41942
ENSMUSG00000073490	AI607873	0.0156242	-2.41758
ENSMUSG00000024451	Arap3	0.0156392	-2.41723
ENSMUSG00000040613	Apobec1	0.0157454	-2.41476
ENSMUSG00000017677	Wsb1	0.0159297	-2.41052
ENSMUSG00000002699	Lcp2	0.0159493	-2.41007
ENSMUSG00000004952	Rasa4	0.0160135	-2.40861
ENSMUSG00000032253	Phip	0.0161076	-2.40647
ENSMUSG00000024736	Tmem132a	0.0162063	-2.40424
ENSMUSG00000001128	Cfp	0.0162301	-2.4037
ENSMUSG00000058099	Nfam1	0.0162481	-2.40329
ENSMUSG00000020642	Rnf144a	0.0162637	-2.40294
ENSMUSG00000030830	Itgal	0.0163333	-2.40138
ENSMUSG00000043873	Chi3l7	0.016399	-2.39991
ENSMUSG00000097134	1110002J07Rik	0.0165961	-2.39553
ENSMUSG00000085058	8030453O22Rik	0.016657	-2.39419
ENSMUSG00000046410	Kcnk6	0.0166595	-2.39414
ENSMUSG00000014543	Klra17	0.0169092	-2.38868
ENSMUSG00000038811	Gngt2	0.016936	-2.38809
ENSMUSG00000021190	Lgmn	0.0169631	-2.38751
ENSMUSG00000020785	Camkk1	0.0173605	-2.37898

ENSMUSG00000028556	Dock7	0.0173711	-2.37876
ENSMUSG00000029084	Cd38	0.0173893	-2.37837
ENSMUSG00000098112	Bin2	0.0174238	-2.37764
ENSMUSG00000040009	Gnaz	0.0176115	-2.37369
ENSMUSG00000042671	Rgs8	0.0176264	-2.37337
ENSMUSG00000038415	Foxq1	0.0176863	-2.37212
ENSMUSG00000056130	Ticam2	0.0177809	-2.37015
ENSMUSG00000015354	Pcolce2	0.0178106	-2.36953
ENSMUSG00000051439	Cd14	0.0178138	-2.36947
ENSMUSG00000032198	Dock6	0.0178297	-2.36913
ENSMUSG00000042349	Ikbke	0.0179197	-2.36727
ENSMUSG00000058897	Col25a1	0.0181187	-2.36318
ENSMUSG00000030761	Myo7a	0.0182512	-2.36048
ENSMUSG00000098893	RP23-292F17.4	0.0183613	-2.35825
ENSMUSG00000036587	Fut7	0.0183699	-2.35808
ENSMUSG00000038600	Atp6v0a4	0.0185779	-2.35389
ENSMUSG00000042659	Arrdc4	0.0185867	-2.35372
ENSMUSG00000030878	Cdr2	0.0185875	-2.3537
ENSMUSG00000055313	Pgbd1	0.0186121	-2.35321
ENSMUSG00000049775	Tmsb4x	0.0186128	-2.3532
ENSMUSG00000040037	Negr1	0.0186138	-2.35318
ENSMUSG00000032572	Col6a4	0.0186231	-2.35299
ENSMUSG00000034764	1700006J14Rik	0.0187407	-2.35065
ENSMUSG00000018008	Cyth4	0.0187715	-2.35004
ENSMUSG00000002885	Cd97	0.0188939	-2.34762
ENSMUSG00000031639	Tlr3	0.0190401	-2.34474
ENSMUSG00000027379	Bub1	0.019215	-2.34133
ENSMUSG00000089917	Uckl1	0.0192625	-2.34041
ENSMUSG00000039109	F13a1	0.0192727	-2.34021
ENSMUSG00000098557	KCTD12	0.0192919	-2.33984
ENSMUSG00000098889	RP23-393F23.11	0.0193819	-2.3381
ENSMUSG00000036894	Rap2b	0.019514	-2.33556
ENSMUSG00000056069	Fam105a	0.0195953	-2.33401
ENSMUSG00000041707	1810011H11Rik	0.0196238	-2.33346
ENSMUSG00000024521	Pmaip1	0.0200302	-2.32578
ENSMUSG00000021596	Mctp1	0.0200449	-2.32551
ENSMUSG00000034652	Cd300a	0.0202208	-2.32223
ENSMUSG00000067916	Gm13139	0.0202621	-2.32146
ENSMUSG00000058571	Gpc6	0.0203461	-2.3199
ENSMUSG00000056268	Dennd1b	0.0203783	-2.31931
ENSMUSG00000031864	Ints10	0.020465	-2.31771
ENSMUSG00000033777	Tlr13	0.0205344	-2.31644
ENSMUSG00000000290	Itgb2	0.0206373	-2.31456
ENSMUSG00000056124	B4galt6	0.0210742	-2.30665
ENSMUSG00000023913	Pla2g7	0.0210833	-2.30649
ENSMUSG00000030173	Klra5	0.0211026	-2.30614
ENSMUSG00000022974	Paxbp1	0.02124	-2.30369
ENSMUSG00000096140	Ankrd66	0.0212577	-2.30338
ENSMUSG00000044702	Palb2	0.0214609	-2.29978
ENSMUSG00000018899	Irf1	0.0221272	-2.28818
ENSMUSG00000051506	Wdfy4	0.0221317	-2.2881
ENSMUSG00000022488	Nckap1l	0.0224606	-2.28249
ENSMUSG00000068134	Zfp120	0.0225126	-2.28161

ENSMUSG00000025083	Afap1l2	0.0225231	-2.28143
ENSMUSG00000006411	Pvrl4	0.0226889	-2.27863
ENSMUSG00000001300	Efnb2	0.0226916	-2.27859
ENSMUSG00000021250	Fos	0.0227428	-2.27773
ENSMUSG00000036918	Ttc7	0.0229234	-2.27471
ENSMUSG00000026987	Baz2b	0.0229948	-2.27352
ENSMUSG00000015627	Gata5	0.0229968	-2.27349
ENSMUSG00000062210	Tnfaip8	0.0230732	-2.27222
ENSMUSG00000083161	Gm11427	0.0231268	-2.27133
ENSMUSG00000074281	Gm12522	0.0231436	-2.27106
ENSMUSG00000033540	Idua	0.023201	-2.27011
ENSMUSG00000030223	Ptpro	0.023214	-2.26989
ENSMUSG00000041842	Fhdc1	0.0232162	-2.26986
ENSMUSG00000044229	Nxpe4	0.0233574	-2.26754
ENSMUSG00000061411	8430427H17Rik	0.0235275	-2.26476
ENSMUSG00000031903	Pla2g15	0.0238132	-2.26013
ENSMUSG00000086109	Gm13391	0.0239581	-2.2578
ENSMUSG00000020490	Btnl10	0.0240963	-2.25559
ENSMUSG00000030579	Tyrobp	0.0242653	-2.2529
ENSMUSG00000066684	Pilrb1	0.0243003	-2.25235
ENSMUSG00000035967	Ddx26b	0.0243541	-2.2515
ENSMUSG00000020227	Irak3	0.0243919	-2.2509
ENSMUSG00000036036	Zfp57	0.0247731	-2.24492
ENSMUSG00000042233	2010015L04Rik	0.0248071	-2.24439
ENSMUSG00000042638	Gucy2c	0.0251114	-2.23969
ENSMUSG00000064210	Ano6	0.0252439	-2.23765
ENSMUSG00000022150	Dab2	0.0253853	-2.23549
ENSMUSG00000027695	Pld1	0.0255874	-2.23242
ENSMUSG00000032812	Arap1	0.0257727	-2.22962
ENSMUSG00000026548	Slamf9	0.0258494	-2.22847
ENSMUSG00000039304	Tnfsf10	0.025941	-2.22709
ENSMUSG00000039831	Arhgap29	0.0259676	-2.2267
ENSMUSG00000097705	Gm26740	0.0260811	-2.225
ENSMUSG00000031543	Ank1	0.0261309	-2.22426
ENSMUSG00000052384	Nrros	0.0261633	-2.22378
ENSMUSG00000047502	Mroh7	0.0263375	-2.2212
ENSMUSG00000063458	1700112E06Rik	0.0263599	-2.22087
ENSMUSG00000024885	Aldh3b1	0.0263694	-2.22073
ENSMUSG00000026482	Rgl1	0.0264113	-2.22011
ENSMUSG00000030000	Add2	0.026431	-2.21982
ENSMUSG00000037685	Atp8a1	0.0264632	-2.21935
ENSMUSG00000068394	Cep152	0.0265194	-2.21852
ENSMUSG00000050989	Sepn1	0.0265812	-2.21761
ENSMUSG00000074676	Foxs1	0.026588	-2.21751
ENSMUSG00000020949	Fkbp3	0.0265928	-2.21744
ENSMUSG00000025348	Itga7	0.0269752	-2.21188
ENSMUSG00000034613	Ppm1h	0.0270415	-2.21092
ENSMUSG00000062082	Cd200r4	0.0270985	-2.2101
ENSMUSG00000078994	Zfp429	0.0271045	-2.21001
ENSMUSG00000036905	C1qb	0.0271776	-2.20896
ENSMUSG00000020388	Pdlim4	0.0273523	-2.20645
ENSMUSG00000024169	Ift140	0.027481	-2.20462
ENSMUSG00000051723	Rpl31-ps13	0.027535	-2.20385

ENSMUSG00000066613	Zfp932	0.0275713	-2.20333
ENSMUSG00000028894	Inpp5b	0.0275986	-2.20294
ENSMUSG00000071042	Rasgrp3	0.0277253	-2.20115
ENSMUSG00000081855	Rpl17-ps5	0.0277577	-2.20069
ENSMUSG00000031925	Maml2	0.0277959	-2.20016
ENSMUSG00000024030	Abcg1	0.0279457	-2.19805
ENSMUSG00000071267	Zfp942	0.0282507	-2.19379
ENSMUSG00000024379	Tslp	0.0286001	-2.18896
ENSMUSG00000045464	2810002D19Rik	0.0287153	-2.18737
ENSMUSG00000082433	Gm9025	0.0287743	-2.18657
ENSMUSG00000036943	Rab8b	0.0287867	-2.1864
ENSMUSG00000020892	Aloxe3	0.0288299	-2.1858
ENSMUSG00000020052	Ascl1	0.0289459	-2.18422
ENSMUSG00000040552	C3ar1	0.0290844	-2.18234
ENSMUSG00000072501	Phf20l1	0.0291644	-2.18126
ENSMUSG00000092384	Gm4189	0.029229	-2.18038
ENSMUSG00000021519	Mterfd1	0.0293122	-2.17926
ENSMUSG00000019960	Dusp6	0.0293753	-2.17841
ENSMUSG00000033705	Stard9	0.0294502	-2.17741
ENSMUSG00000025876	Unc5a	0.0294635	-2.17723
ENSMUSG00000017716	Birc5	0.0294636	-2.17723
ENSMUSG00000044827	Tlr1	0.0296761	-2.17439
ENSMUSG00000027423	Snx5	0.0298734	-2.17176
ENSMUSG00000059498	Fcgr3	0.0300898	-2.16891
ENSMUSG00000036459	Wtip	0.0302327	-2.16703
ENSMUSG00000074677	Sirpb1c	0.0306265	-2.16189
ENSMUSG00000068335	Dok1	0.0308428	-2.15909
ENSMUSG00000030970	Ctbp2	0.0308961	-2.15841
ENSMUSG00000043391	2510009E07Rik	0.0311807	-2.15476
ENSMUSG00000056220	Pla2g4a	0.0313319	-2.15283
ENSMUSG00000059708	Akap17b	0.0314057	-2.15189
ENSMUSG00000021458	2010111I01Rik	0.0314607	-2.1512
ENSMUSG00000097567	Gm26637	0.0314776	-2.15098
ENSMUSG00000027954	Efna1	0.0316602	-2.14867
ENSMUSG00000002458	Rgs19	0.0318335	-2.14649
ENSMUSG00000034786	Gpsm3	0.0318344	-2.14648
ENSMUSG00000083007	Gm13623	0.0318918	-2.14576
ENSMUSG00000032125	Robo4	0.0320112	-2.14427
ENSMUSG00000073400	Trim10	0.0320949	-2.14323
ENSMUSG00000030677	Kif22	0.0321474	-2.14257
ENSMUSG00000041754	Trem3	0.0322185	-2.14169
ENSMUSG00000046182	Gsg1l	0.0324014	-2.13942
ENSMUSG00000084350	Znf41-ps	0.0324936	-2.13829
ENSMUSG00000055430	Nap1l5	0.0325675	-2.13737
ENSMUSG00000071203	Naip5	0.0325892	-2.13711
ENSMUSG00000096950	Gm9530	0.03276	-2.13501
ENSMUSG00000056413	Adap1	0.0328177	-2.13431
ENSMUSG00000060771	Tsga10	0.0330096	-2.13197
ENSMUSG00000000204	Slfn4	0.0332786	-2.12871
ENSMUSG00000038903	Ccdc68	0.0333609	-2.12771
ENSMUSG00000050761	Gp1bb	0.0337433	-2.12313
ENSMUSG00000022584	Ly6c2	0.0337812	-2.12267
ENSMUSG00000040035	Disp2	0.033836	-2.12202

ENSMUSG00000037376	Trmt6	0.0338563	-2.12178
ENSMUSG00000024985	Tcf7l2	0.033895	-2.12132
ENSMUSG00000085457	1110046J04Rik	0.0339111	-2.12113
ENSMUSG00000070390	Nlrp1b	0.033942	-2.12076
ENSMUSG00000039671	Zmynd8	0.0340542	-2.11943
ENSMUSG00000018845	Unc45b	0.0340683	-2.11926
ENSMUSG00000022822	Abcc5	0.0341067	-2.11881
ENSMUSG00000015501	Hivep2	0.0341612	-2.11816
ENSMUSG00000023926	Rhag	0.0341891	-2.11783
ENSMUSG00000051682	Treml4	0.0342882	-2.11667
ENSMUSG00000036622	Atp13a2	0.0344	-2.11535
ENSMUSG00000028717	Tal1	0.0344247	-2.11506
ENSMUSG00000079559	Gm684	0.0344265	-2.11504
ENSMUSG00000037946	Fgd3	0.0344834	-2.11437
ENSMUSG00000020469	Myl7	0.0345002	-2.11418
ENSMUSG00000040016	Ptger3	0.0345112	-2.11405
ENSMUSG00000050966	Lin28a	0.0345841	-2.1132
ENSMUSG00000043065	Spice1	0.0345885	-2.11314
ENSMUSG00000070873	Lilra5	0.0346123	-2.11286
ENSMUSG00000051444	Bbs12	0.034625	-2.11272
ENSMUSG00000083041	Gm9835	0.0348059	-2.11061
ENSMUSG00000014782	Plekhg4	0.0349377	-2.10908
ENSMUSG00000053063	Clec12a	0.0349698	-2.10871
ENSMUSG00000040322	Slc25a24	0.0352336	-2.10566
ENSMUSG00000063652	Slc22a21	0.0352738	-2.1052
ENSMUSG00000035352	Ccl12	0.035417	-2.10356
ENSMUSG00000002103	Acp2	0.0354307	-2.1034
ENSMUSG00000039910	Cited2	0.0354981	-2.10263
ENSMUSG00000022123	Scel	0.0355009	-2.1026
ENSMUSG00000025982	Sf3b1	0.035514	-2.10245
ENSMUSG00000056671	Prelid2	0.0357394	-2.09988
ENSMUSG00000032690	Oas2	0.0358097	-2.09908
ENSMUSG00000024679	Ms4a6d	0.0358751	-2.09834
ENSMUSG00000032000	Birc3	0.0359411	-2.09759
ENSMUSG00000021028	Mbip	0.0359729	-2.09723
ENSMUSG00000037731	Themis2	0.0360374	-2.09651
ENSMUSG00000000320	Alox12	0.0360605	-2.09624
ENSMUSG00000091694	Apol11b	0.036091	-2.0959
ENSMUSG00000047246	Hist1h2be	0.036173	-2.09498
ENSMUSG00000082029	H3f3c	0.0362441	-2.09418
ENSMUSG00000028164	Manba	0.036442	-2.09196
ENSMUSG00000003665	Has1	0.0364493	-2.09188
ENSMUSG00000014850	Msh3	0.0365407	-2.09086
ENSMUSG00000027293	Ehd4	0.0366721	-2.0894
ENSMUSG00000029330	Cds1	0.0368572	-2.08734
ENSMUSG00000032363	Adamts7	0.0371131	-2.08452
ENSMUSG00000023484	Prph	0.0371796	-2.08379
ENSMUSG00000000628	Hk2	0.037305	-2.08241
ENSMUSG00000056579	Tug1	0.0375289	-2.07996
ENSMUSG00000032254	Kif23	0.037594	-2.07925
ENSMUSG00000051517	Arhgef39	0.0378711	-2.07625
ENSMUSG00000037370	Enpp1	0.0378715	-2.07624
ENSMUSG00000020773	Trim47	0.0381404	-2.07334

ENSMUSG00000026395	Ptprc	0.0381494	-2.07325
ENSMUSG00000031736	4933436C20Rik	0.0381927	-2.07278
ENSMUSG00000014158	Trpv4	0.0381939	-2.07277
ENSMUSG00000026319	2310035C23Rik	0.0383485	-2.07111
ENSMUSG00000097790	Gm16399	0.0383526	-2.07107
ENSMUSG00000074874	Ctla2b	0.0384424	-2.07011
ENSMUSG00000019966	Kitl	0.0384762	-2.06975
ENSMUSG00000086495	Gm13778	0.038528	-2.06919
ENSMUSG00000036553	Sh3tc1	0.0387228	-2.06712
ENSMUSG00000000579	Dynlt1c	0.0388392	-2.06589
ENSMUSG00000098679	mmu-mir-7688	0.0388736	-2.06552
ENSMUSG00000022000	Zc3h13	0.0389193	-2.06504
ENSMUSG00000021423	Ly86	0.0389876	-2.06432
ENSMUSG00000060470	Gpr97	0.0390189	-2.06399
ENSMUSG00000053113	Socs3	0.0390464	-2.0637
ENSMUSG00000032409	Atr	0.0390938	-2.0632
ENSMUSG00000071561	BC100530	0.0392036	-2.06204
ENSMUSG00000037336	Mfsd2b	0.0396384	-2.0575
ENSMUSG00000039005	Tlr4	0.0396491	-2.05739
ENSMUSG00000007440	Pcdha2	0.0397534	-2.0563
ENSMUSG00000050473	Slc35d3	0.039924	-2.05453
ENSMUSG00000097572	Gm26797	0.0399905	-2.05385
ENSMUSG00000021639	Gtf2h2	0.0399966	-2.05378
ENSMUSG00000034911	Ushbp1	0.0400418	-2.05332
ENSMUSG00000010311	Optc	0.0402874	-2.05079
ENSMUSG00000062861	Zfp28	0.0404268	-2.04936
ENSMUSG00000038679	Trps1	0.0404322	-2.04931
ENSMUSG00000071713	Csf2rb	0.0405819	-2.04778
ENSMUSG00000020101	4632428N05Rik	0.0408432	-2.04512
ENSMUSG00000022148	Fyb	0.0408846	-2.0447
ENSMUSG00000032586	Traip	0.0411243	-2.04227
ENSMUSG00000090626	Tex9	0.0412816	-2.04069
ENSMUSG00000032089	Il10ra	0.0413389	-2.04012
ENSMUSG00000002602	Axl	0.0413673	-2.03983
ENSMUSG00000046080	Clec9a	0.0414404	-2.0391
ENSMUSG00000021706	Zfyve16	0.0414838	-2.03866
ENSMUSG00000022973	Synj1	0.041556	-2.03794
ENSMUSG00000094344	Gm11942	0.041634	-2.03716
ENSMUSG00000045629	Sh3tc2	0.0416486	-2.03701
ENSMUSG00000019845	Tube1	0.0417844	-2.03566
ENSMUSG00000095028	Sirpb1b	0.0420251	-2.03327
ENSMUSG00000087235	Gm4750	0.0420992	-2.03254
ENSMUSG00000082729	Gm14845	0.0421098	-2.03243
ENSMUSG00000035713	Usp35	0.0422217	-2.03133
ENSMUSG00000021224	Numb	0.0423239	-2.03032
ENSMUSG00000000759	Tubgcp3	0.04239	-2.02967
ENSMUSG00000030472	Ceacam18	0.0424274	-2.0293
ENSMUSG00000025044	Msr1	0.0425207	-2.02839
ENSMUSG00000012123	Aim1l	0.0427478	-2.02617
ENSMUSG00000038022	Fam188b	0.0427654	-2.02599
ENSMUSG00000047565	Acot10	0.0427734	-2.02592
ENSMUSG00000039456	Morc3	0.0428872	-2.02481
ENSMUSG00000051579	Tceal8	0.0430388	-2.02333

ENSMUSG00000016087	Fli1	0.0432605	-2.02119
ENSMUSG00000026872	Zeb2	0.0433416	-2.0204
ENSMUSG00000002111	Spi1	0.0433442	-2.02038
ENSMUSG00000086866	4930512H18Rik	0.0435563	-2.01834
ENSMUSG00000027962	Vcam1	0.0436306	-2.01762
ENSMUSG00000053178	Gm9897	0.0437363	-2.01661
ENSMUSG00000032400	Zwilch	0.0437767	-2.01622
ENSMUSG00000023349	Clec4n	0.043827	-2.01574
ENSMUSG00000040084	Bub1b	0.0438342	-2.01567
ENSMUSG00000034209	Rasl10a	0.0439788	-2.01429
ENSMUSG00000036634	Mag	0.0439923	-2.01416
ENSMUSG00000050195	Scd4	0.0441072	-2.01307
ENSMUSG00000030209	Grin2b	0.0441989	-2.0122
ENSMUSG00000041515	Irf8	0.0442604	-2.01162
ENSMUSG00000026632	Tatdn3	0.0442928	-2.01131
ENSMUSG00000039977	Ccdc67	0.0444532	-2.00979
ENSMUSG00000079808	AC168977.1	0.0447993	-2.00653
ENSMUSG00000000184	Ccnd2	0.044892	-2.00567
ENSMUSG00000007207	Stx1a	0.0449601	-2.00503
ENSMUSG00000030170	Wnt5b	0.0451135	-2.00359
ENSMUSG00000022220	Adcy4	0.0452801	-2.00204
ENSMUSG00000080715	Gm5406	0.0452846	-2.002
ENSMUSG00000006519	Cyba	0.0455686	-1.99937
ENSMUSG00000009418	Nav1	0.04596	-1.99576
ENSMUSG00000078502	Gm13212	0.0459621	-1.99574
ENSMUSG00000022018	Rgcc	0.0459759	-1.99561
ENSMUSG00000030022	Adamts9	0.0461093	-1.99439
ENSMUSG00000055639	Dach1	0.0461884	-1.99367
ENSMUSG00000026627	Tmem206	0.0463387	-1.9923
ENSMUSG00000030020	Prickle2	0.0465369	-1.99049
ENSMUSG00000090936	Gm17705	0.0465566	-1.99031
ENSMUSG00000040964	Arhgef10l	0.0466942	-1.98906
ENSMUSG00000079845	Xlr4a	0.0467349	-1.9887
ENSMUSG00000027313	Chac1	0.0467622	-1.98845
ENSMUSG00000027398	Il1b	0.0469014	-1.98719
ENSMUSG00000030649	Anapc15	0.0469274	-1.98695
ENSMUSG00000000266	Mid2	0.0469903	-1.98639
ENSMUSG00000038147	Cd84	0.0470723	-1.98565
ENSMUSG00000006281	Tep1	0.0471368	-1.98507
ENSMUSG00000071796	6820431F20Rik	0.0473755	-1.98293
ENSMUSG00000018727	Cpsf4l	0.0473821	-1.98287
ENSMUSG00000026657	Frmd4a	0.047418	-1.98255
ENSMUSG00000026196	Bard1	0.0475248	-1.98159
ENSMUSG00000042050	Wdr60	0.0475414	-1.98144
ENSMUSG00000034438	Gbp8	0.0477806	-1.97931
ENSMUSG00000067931	Zfp948	0.0478736	-1.97849
ENSMUSG00000031824	6430548M08Rik	0.0480836	-1.97663
ENSMUSG00000036887	C1qa	0.0483552	-1.97423
ENSMUSG00000085282	Gm15663	0.0484349	-1.97353
ENSMUSG00000070476	Fam217b	0.0484457	-1.97344
ENSMUSG00000073650	Gm216	0.0484777	-1.97316
ENSMUSG00000056553	Ptprn2	0.048552	-1.9725
ENSMUSG00000012640	Zfp715	0.0486311	-1.97181

ENSMUSG00000097327	E030030I06Rik	0.0487618	-1.97067
ENSMUSG00000022469	Rapgef3	0.0489429	-1.96909
ENSMUSG00000032436	Cmtm7	0.0490306	-1.96833
ENSMUSG00000049687	Fam109b	0.0493049	-1.96595
ENSMUSG00000019982	Myb	0.04948	-1.96443
ENSMUSG00000037386	Rims2	0.0496144	-1.96327
ENSMUSG00000030921	Trim30a	0.0496974	-1.96256
ENSMUSG00000086438	2510003D18Rik	0.0497134	-1.96242
ENSMUSG00000000711	Rab5b	0.0499188	1.96066
ENSMUSG00000037151	Lrrc20	0.0496441	1.96302
ENSMUSG00000096232	0610012G03Rik	0.0491877	1.96696
ENSMUSG00000008393	Carhsp1	0.0490732	1.96796
ENSMUSG00000081700	Gm14337	0.0488659	1.96976
ENSMUSG00000057440	Mpp7	0.0486771	1.97141
ENSMUSG00000032418	Me1	0.0484096	1.97375
ENSMUSG00000044378	Slc15a5	0.0483793	1.97402
ENSMUSG00000078570	1110065P20Rik	0.0482555	1.97511
ENSMUSG00000022972	1110004E09Rik	0.0481215	1.97629
ENSMUSG00000020107	Anapc16	0.0477784	1.97933
ENSMUSG00000025781	Atp5c1	0.0476996	1.98003
ENSMUSG00000024516	Sec11c	0.0476726	1.98028
ENSMUSG00000083700	Gm2986	0.0476037	1.98089
ENSMUSG00000006050	Sra1	0.0475878	1.98103
ENSMUSG00000030795	Fus	0.0474863	1.98194
ENSMUSG00000002768	Mea1	0.0474847	1.98195
ENSMUSG00000020657	Dnajc27	0.0473706	1.98297
ENSMUSG00000047187	Rab2a	0.0467935	1.98816
ENSMUSG00000081441	Gm6161	0.0463821	1.9919
ENSMUSG00000059022	Kcp	0.0462185	1.99339
ENSMUSG00000022620	Arsa	0.0461572	1.99395
ENSMUSG00000030521	Mphosph10	0.0461198	1.9943
ENSMUSG00000071470	Ccnb1ip1	0.0460226	1.99519
ENSMUSG00000003429	Rps11	0.0456952	1.9982
ENSMUSG00000046607	Hrk	0.0453817	2.0011
ENSMUSG00000025380	Fscn2	0.0453628	2.00127
ENSMUSG00000004264	Phb2	0.0449967	2.00469
ENSMUSG00000034430	Zxdc	0.0449858	2.00479
ENSMUSG00000061356	Nuggc	0.0449471	2.00515
ENSMUSG00000003299	Mrpl4	0.0449438	2.00518
ENSMUSG00000066771	Gm5614	0.0448853	2.00573
ENSMUSG00000004789	Dlst	0.0448765	2.00581
ENSMUSG00000087512	Gm11985	0.0446625	2.00782
ENSMUSG00000060002	Chpt1	0.0444275	2.01004
ENSMUSG00000006782	Cnp	0.0443531	2.01074
ENSMUSG00000035621	Midn	0.044146	2.0127
ENSMUSG00000033752	Mnd1	0.043852	2.0155
ENSMUSG00000042043	Tbca	0.0436701	2.01724
ENSMUSG00000022132	Cldn10	0.0434798	2.01907
ENSMUSG00000003135	Cnot11	0.0434436	2.01942
ENSMUSG00000097158	Gm26516	0.0431771	2.02199
ENSMUSG00000047459	Dynlrb1	0.0431004	2.02274
ENSMUSG00000057181	5730455P16Rik	0.0430972	2.02277
ENSMUSG00000073492	Gm10521	0.0430495	2.02323

ENSMUSG00000064767	Snord35b	0.0426284	2.02733
ENSMUSG00000076543	Igkv4-74	0.0426131	2.02748
ENSMUSG00000073842	Mup7	0.0425632	2.02797
ENSMUSG00000078787	Cyp2t4	0.0423375	2.03019
ENSMUSG00000020526	Znhit3	0.0422471	2.03108
ENSMUSG00000032593	Amigo3	0.0419974	2.03355
ENSMUSG00000026750	Psmb7	0.0419043	2.03447
ENSMUSG00000009092	Derl3	0.0418205	2.0353
ENSMUSG00000078713	Tomm5	0.0415514	2.03799
ENSMUSG00000023971	Rrp36	0.0413559	2.03994
ENSMUSG00000068184	Ndufaf2	0.0412521	2.04099
ENSMUSG00000030498	Gas2	0.0412193	2.04132
ENSMUSG00000097971	Gm26917	0.0411749	2.04176
ENSMUSG00000047222	Ear11	0.0411412	2.0421
ENSMUSG00000005846	Rsl1d1	0.0410849	2.04267
ENSMUSG00000090171	Ugt1a2	0.0408683	2.04486
ENSMUSG00000046691	Chtf8	0.0408547	2.045
ENSMUSG00000032869	Psmf1	0.0405789	2.04781
ENSMUSG00000005683	Cs	0.0402595	2.05108
ENSMUSG00000080893	Gm15920	0.0401313	2.0524
ENSMUSG00000097737	Gm26530	0.0398623	2.05517
ENSMUSG00000035539	E230008N13Rik	0.0398452	2.05535
ENSMUSG00000041438	Cirh1a	0.0396939	2.05692
ENSMUSG00000073856	Iqck	0.0396576	2.0573
ENSMUSG00000040158	Tax1bp3	0.0395582	2.05833
ENSMUSG00000083482	Gm14706	0.0394235	2.05974
ENSMUSG00000030689	Ino80e	0.0392354	2.06171
ENSMUSG00000038599	Capn8	0.0392089	2.06199
ENSMUSG00000062061	Obp2a	0.0391975	2.06211
ENSMUSG00000019326	Aoc3	0.0391108	2.06302
ENSMUSG00000019179	Mdh2	0.0390083	2.0641
ENSMUSG00000010914	Pdhx	0.038993	2.06426
ENSMUSG00000027012	Dync1i2	0.0388548	2.06572
ENSMUSG00000002015	Bcap31	0.0388055	2.06624
ENSMUSG00000028948	Nol9	0.0387848	2.06646
ENSMUSG00000018446	C1qbp	0.0385803	2.06864
ENSMUSG00000098161	Gm26975	0.0384123	2.07043
ENSMUSG00000022247	Brix1	0.0383187	2.07143
ENSMUSG00000026974	Zmynd19	0.0383103	2.07152
ENSMUSG00000020014	Gm872	0.0380263	2.07457
ENSMUSG00000055900	Tmem69	0.0379999	2.07486
ENSMUSG00000060240	Cend1	0.0379654	2.07523
ENSMUSG00000066357	Wdr6	0.0376784	2.07834
ENSMUSG00000031819	Emc8	0.0375885	2.07931
ENSMUSG00000095478	Gm9824	0.0375723	2.07949
ENSMUSG00000041697	Cox6a1	0.0373255	2.08219
ENSMUSG00000029198	Grpel1	0.0372086	2.08347
ENSMUSG00000083937	Cct3-ps1	0.0370563	2.08514
ENSMUSG00000024732	Ccdc86	0.0370272	2.08546
ENSMUSG00000044441	Olfr1442	0.0368144	2.08782
ENSMUSG00000085834	Gm15622	0.0366103	2.09008
ENSMUSG00000016319	Slc25a5	0.0364657	2.0917
ENSMUSG00000048644	Ctxn1	0.036239	2.09424

ENSMUSG00000005469	Prkaca	0.0362288	2.09435
ENSMUSG00000097322	A530083I20Rik	0.0361739	2.09497
ENSMUSG00000029229	Chic2	0.0360972	2.09583
ENSMUSG00000025432	Avil	0.035988	2.09706
ENSMUSG00000043671	Dpy19l3	0.0358131	2.09904
ENSMUSG00000038150	Ormdl3	0.0356287	2.10114
ENSMUSG00000083621	Gm14586	0.0355935	2.10154
ENSMUSG00000048534	Amica1	0.0355057	2.10254
ENSMUSG00000062464	Gm9705	0.0351192	2.10698
ENSMUSG00000026615	Eprs	0.0350702	2.10755
ENSMUSG00000029634	Rnf6	0.0347706	2.11102
ENSMUSG00000028277	Ube2j1	0.0347396	2.11138
ENSMUSG00000001542	Ell2	0.034719	2.11162
ENSMUSG00000096995	2810029C07Rik	0.0344642	2.1146
ENSMUSG00000032482	Cspg5	0.0343695	2.11571
ENSMUSG00000087346	Gm5699	0.0343224	2.11626
ENSMUSG00000051504	Siglech	0.0342135	2.11755
ENSMUSG00000014177	Tvp23b	0.0340839	2.11908
ENSMUSG00000024645	Timm21	0.0339126	2.12111
ENSMUSG00000083468	Gm9431	0.0338042	2.1224
ENSMUSG00000059409	Ppp2r5d	0.0337279	2.12331
ENSMUSG00000019178	Styxl1	0.0337001	2.12364
ENSMUSG00000076038	Gm22774	0.0335614	2.1253
ENSMUSG00000083832	Gm13771	0.0328219	2.13425
ENSMUSG00000021981	Cab39l	0.0327974	2.13455
ENSMUSG00000054385	Ceacam2	0.0327175	2.13553
ENSMUSG00000083610	Oaz2-ps	0.0325748	2.13729
ENSMUSG00000020826	Nos2	0.0323868	2.1396
ENSMUSG00000028211	Trp53inp1	0.032334	2.14026
ENSMUSG00000030278	Cidec	0.0321479	2.14257
ENSMUSG00000044211	Gm7887	0.032144	2.14262
ENSMUSG00000043207	Zmpste24	0.03209	2.14329
ENSMUSG00000053219	Raet1e	0.0312229	2.15422
ENSMUSG00000090121	Abhd12b	0.0311985	2.15453
ENSMUSG00000030611	Mrps11	0.0311209	2.15552
ENSMUSG00000066880	Zfp617	0.0310426	2.15653
ENSMUSG00000046791	2410016O06Rik	0.0306369	2.16176
ENSMUSG00000025868	Higd2a	0.0303398	2.16563
ENSMUSG00000078894	2210418O10Rik	0.0302108	2.16732
ENSMUSG00000059796	Eif4a1	0.030163	2.16794
ENSMUSG00000034729	Mrps10	0.030118	2.16854
ENSMUSG00000003346	Abhd17a	0.0300552	2.16936
ENSMUSG00000097412	1810014B01Rik	0.0298642	2.17189
ENSMUSG00000031818	Cox4i1	0.0295694	2.17581
ENSMUSG00000019303	Psmc3ip	0.0294145	2.17789
ENSMUSG00000025780	Itih5	0.029359	2.17863
ENSMUSG00000024873	Cnih2	0.029298	2.17945
ENSMUSG00000044566	Cage1	0.0291911	2.1809
ENSMUSG00000086341	Gm15932	0.0290207	2.18321
ENSMUSG00000092458	Gm18737	0.0288556	2.18545
ENSMUSG00000000753	Serpinf1	0.0285632	2.18946
ENSMUSG00000073633	Fbxo36	0.0284882	2.1905
ENSMUSG00000031879	Fam96b	0.0283652	2.1922

ENSMUSG00000086748	Gm13261	0.0281594	2.19506
ENSMUSG00000013822	Elof1	0.028132	2.19544
ENSMUSG00000030364	Clec2h	0.0280903	2.19602
ENSMUSG00000086962	Gm12248	0.0280705	2.1963
ENSMUSG00000045648	Vwc2l	0.0279429	2.19809
ENSMUSG00000087286	Gm9013	0.0275511	2.20362
ENSMUSG00000020935	Dcakd	0.026665	2.21639
ENSMUSG00000082272	Gm11675	0.0264004	2.22027
ENSMUSG00000039096	Rsad1	0.0263421	2.22113
ENSMUSG00000078185	Chml	0.0263051	2.22168
ENSMUSG00000097536	2610037D02Rik	0.0263009	2.22174
ENSMUSG00000025795	Rassf3	0.0262493	2.2225
ENSMUSG00000032551	1110059G10Rik	0.0261864	2.22344
ENSMUSG00000062470	5730577I03Rik	0.0260599	2.22532
ENSMUSG00000086113	4930596I21Rik	0.0258461	2.22852
ENSMUSG00000038335	Tsr1	0.0258292	2.22877
ENSMUSG00000061780	Cfd	0.0250869	2.24006
ENSMUSG00000016520	Lnx2	0.0247801	2.24481
ENSMUSG00000038214	Bend3	0.0247014	2.24604
ENSMUSG00000028224	Nbn	0.0243862	2.25099
ENSMUSG00000098123	Gm4518	0.0240251	2.25673
ENSMUSG00000082954	Gm15504	0.0239602	2.25777
ENSMUSG00000068245	Phf11d	0.0239519	2.2579
ENSMUSG00000090194	Gm16161	0.0236951	2.26204
ENSMUSG00000074622	Mafb	0.0236129	2.26337
ENSMUSG00000091649	Phf11b	0.0235662	2.26413
ENSMUSG00000024870	Rab1b	0.023495	2.26529
ENSMUSG00000027559	Car3	0.0233341	2.26792
ENSMUSG00000032459	Mrps22	0.0231245	2.27137
ENSMUSG00000087502	Gm16091	0.0231212	2.27143
ENSMUSG00000068267	Cenpb	0.023033	2.27289
ENSMUSG00000048647	Exd1	0.0228349	2.27619
ENSMUSG00000028738	Tas1r2	0.0228337	2.27621
ENSMUSG00000032758	Kap	0.0225328	2.28126
ENSMUSG00000049086	Bmyc	0.0224338	2.28294
ENSMUSG00000052496	Pkdrej	0.0222484	2.2861
ENSMUSG00000037296	Lsm1	0.0222396	2.28625
ENSMUSG00000015176	Nolc1	0.0221955	2.287
ENSMUSG00000046994	Mars2	0.021781	2.29417
ENSMUSG00000078291	Gm3940	0.0217717	2.29433
ENSMUSG00000083326	Rpl38-ps1	0.0216862	2.29582
ENSMUSG00000095630	Igkv6-23	0.0216091	2.29717
ENSMUSG00000021590	Spata9	0.0213006	2.30261
ENSMUSG00000061024	Rrs1	0.0206414	2.31448
ENSMUSG00000028539	Artn	0.0206094	2.31506
ENSMUSG00000078683	Mup1	0.0204649	2.31771
ENSMUSG00000028792	Ak2	0.0200709	2.32502
ENSMUSG00000013160	Atp6v0d1	0.0200512	2.32539
ENSMUSG00000035885	Cox8a	0.0199597	2.32711
ENSMUSG00000038576	Susd4	0.0198793	2.32862
ENSMUSG00000042938	Gm14117	0.01974	2.33125
ENSMUSG00000081911	Mrps36-ps2	0.0195705	2.33448
ENSMUSG00000024038	Ndufv3	0.0194855	2.33611

ENSMUSG00000027076	Timm10	0.0192918	2.33984
ENSMUSG00000032026	Rexo2	0.0192293	2.34106
ENSMUSG00000028937	Acot7	0.0192045	2.34154
ENSMUSG00000005949	Ctns	0.0188259	2.34896
ENSMUSG00000032802	Srxn1	0.0182556	2.36039
ENSMUSG00000041733	Coq5	0.0180783	2.36401
ENSMUSG00000081251	Gm12164	0.0176122	2.37367
ENSMUSG00000039474	Wfs1	0.0172832	2.38063
ENSMUSG00000080006	Rps19-ps7	0.0172806	2.38068
ENSMUSG00000087373	Gm15892	0.0170681	2.38524
ENSMUSG00000097930	C330002G04Rik	0.0167827	2.39143
ENSMUSG00000060989	Gm11847	0.0164813	2.39808
ENSMUSG00000070319	Eif3g	0.0163186	2.40171
ENSMUSG00000002831	Plin4	0.0163177	2.40173
ENSMUSG00000031781	Ciapin1	0.0160187	2.40849
ENSMUSG00000040420	Cdh18	0.0156606	2.41673
ENSMUSG00000041202	Pla2g2d	0.0154276	2.42218
ENSMUSG00000087612	A230005M16Rik	0.0153004	2.42519
ENSMUSG00000087541	Gm15830	0.0149627	2.43328
ENSMUSG00000051212	Gpr183	0.0147622	2.43816
ENSMUSG00000002076	Hsf2bp	0.0147163	2.43929
ENSMUSG00000049521	Cdc42ep1	0.0146253	2.44153
ENSMUSG00000023106	Denr	0.0146122	2.44185
ENSMUSG00000073700	Klhl21	0.0145869	2.44248
ENSMUSG00000084622	Gm25545	0.0138525	2.46106
ENSMUSG00000028443	Nudt2	0.0138151	2.46204
ENSMUSG00000050213	Snip1	0.0137891	2.46271
ENSMUSG00000025035	Arl3	0.0137463	2.46382
ENSMUSG00000083852	Gm13007	0.013688	2.46535
ENSMUSG00000028036	Ptgfr	0.0136809	2.46553
ENSMUSG00000045252	Zfp574	0.0136331	2.46679
ENSMUSG00000006641	Slc5a6	0.0135449	2.46911
ENSMUSG00000019338	Zfp687	0.0135411	2.46921
ENSMUSG00000097443	Gm17529	0.0134462	2.47173
ENSMUSG00000071719	Tmem28	0.0134397	2.4719
ENSMUSG00000097891	Gm3650	0.0131758	2.47898
ENSMUSG00000050663	Trhde	0.0127695	2.49013
ENSMUSG00000033809	Alg3	0.012383	2.50104
ENSMUSG00000037573	Tob1	0.0120948	2.50937
ENSMUSG00000037750	Fam222b	0.0119835	2.51263
ENSMUSG00000080896	Gm14567	0.0116555	2.52241
ENSMUSG00000095682	Igkv3-1	0.0116387	2.52291
ENSMUSG00000030256	Bhlhe41	0.011479	2.52777
ENSMUSG00000029152	Ociad1	0.0113377	2.53212
ENSMUSG00000079494	Cml5	0.0112193	2.53579
ENSMUSG00000033326	Kdm4a	0.0111893	2.53673
ENSMUSG00000030124	Lag3	0.0110941	2.53972
ENSMUSG00000054452	Aes	0.0105833	2.55616
ENSMUSG00000030643	Rab30	0.0098828	2.5799
ENSMUSG00000012405	Rpl15	0.00937558	2.59805
ENSMUSG00000070934	Rraga	0.00902481	2.61111
ENSMUSG00000039521	Foxp3	0.00887305	2.61691
ENSMUSG00000044122	Proca1	0.00886547	2.6172

ENSMUSG00000057130	Txnl4a	0.00790017	2.65631
ENSMUSG00000009633	G0s2	0.00767754	2.66593
ENSMUSG00000043439	E130012A19Rik	0.00766167	2.66663
ENSMUSG00000032959	Pebp1	0.00741822	2.67746
ENSMUSG00000020775	Mrpl38	0.00712667	2.69087
ENSMUSG00000022096	Hr	0.00706569	2.69373
ENSMUSG00000078503	Gm13225	0.00676447	2.70822
ENSMUSG00000025066	Sfr1	0.00671586	2.71061
ENSMUSG00000049580	Tsku	0.00659378	2.71669
ENSMUSG00000031490	Eif4ebp1	0.00657158	2.71781
ENSMUSG00000026409	Pfkfb2	0.00651593	2.72062
ENSMUSG00000008999	Bmp7	0.00649121	2.72188
ENSMUSG00000029246	Ppat	0.00634754	2.72927
ENSMUSG00000029201	Ugdh	0.00627605	2.733
ENSMUSG00000047371	Zfp768	0.00615924	2.73918
ENSMUSG00000031548	Sfrp1	0.00597916	2.74892
ENSMUSG00000058600	Rpl30	0.00590633	2.75294
ENSMUSG00000024761	Gm16437	0.00514663	2.79771
ENSMUSG00000081967	Gm14017	0.00482116	2.81875
ENSMUSG00000060803	Gstp1	0.00481651	2.81906
ENSMUSG00000031158	Timm17b	0.00459484	2.83415
ENSMUSG00000084974	Gm15567	0.00451913	2.83945
ENSMUSG00000075268	Gm10819	0.00451369	2.83983
ENSMUSG00000022385	Gtse1	0.00439521	2.84831
ENSMUSG00000025825	Iscu	0.00424536	2.85933
ENSMUSG00000083885	Gm13213	0.00396396	2.88102
ENSMUSG00000097099	Gm9917	0.00394331	2.88266
ENSMUSG00000051361	6030498E09Rik	0.0038186	2.89277
ENSMUSG00000046721	Rpl14-ps1	0.00364713	2.90717
ENSMUSG00000055322	Tns1	0.00351457	2.91873
ENSMUSG00000084797	Gm14321	0.00321326	2.94656
ENSMUSG00000028909	Ptpru	0.00305347	2.9623
ENSMUSG00000019080	Mfsd3	0.00286215	2.98217
ENSMUSG00000094664	Rpl35a-ps6	0.00265031	3.00563
ENSMUSG00000036251	Trpm8	0.00259712	3.01179
ENSMUSG00000080866	Gm5687	0.00254169	3.01833
ENSMUSG00000098753	PYURF	0.00251209	3.02188
ENSMUSG00000029530	Ccr9	0.00245909	3.02833
ENSMUSG00000022237	Ankrd33b	0.00236704	3.03984
ENSMUSG00000027505	Fam209	0.0022037	3.06131
ENSMUSG00000096715	Igkv3-4	0.00215836	3.06753
ENSMUSG00000078937	Cpt1b	0.00215708	3.06771
ENSMUSG00000048546	Tob2	0.00204759	3.08324
ENSMUSG00000095007	Igkv12-41	0.00204471	3.08366
ENSMUSG00000073771	Btbd19	0.00200262	3.08984
ENSMUSG00000031157	Pqbp1	0.00168863	3.14015
ENSMUSG00000020738	Sumo2	0.00156783	3.16183
ENSMUSG00000096188	Cmtm4	0.00142272	3.19
ENSMUSG00000024544	Ldlrad4	0.00141808	3.19095
ENSMUSG00000038550	Gm129	0.00126786	3.22316
ENSMUSG00000087213	2810408I11Rik	0.00125753	3.2255
ENSMUSG00000067608	Pcna-ps2	0.00104067	3.27929
ENSMUSG00000041453	Rpl21	0.000828061	3.34324

ENSMUSG00000087327	Gm15884	0.00079008	3.35625
ENSMUSG00000038060	Dlec1	0.000787458	3.35717
ENSMUSG00000091021	Gm17300	0.000617877	3.42364
ENSMUSG00000006218	Fam131c	0.000608872	3.42763
ENSMUSG00000038155	Gstp2	0.000468113	3.49837
ENSMUSG00000051675	Trim32	0.000463302	3.50112
ENSMUSG00000020673	Tpo	0.000337442	3.58472
ENSMUSG00000039220	Ppp1r10	0.000287898	3.62595
ENSMUSG00000053469	Tg	0.00027817	3.63482
ENSMUSG00000036160	Surf6	0.000269554	3.64293
ENSMUSG00000081562	Gm11575	0.000177303	3.74934
ENSMUSG00000043424	Eif3j2	0.000167244	3.76396
ENSMUSG00000067567	Hdac8	0.000131186	3.82422
ENSMUSG00000094793	Mup12	6.58085E-05	3.99095
ENSMUSG00000032220	Myo1e	6.05513E-05	4.01065
ENSMUSG00000020776	Fbf1	4.56617E-05	4.07679
ENSMUSG00000022039	Adam2	8.76457E-06	4.4456
ENSMUSG00000062588	Gm6104	4.35199E-07	5.05288
